# Proposal of names for 329 higher rank taxa defined in the Genome Taxonomy Database under two prokaryotic codes

**DOI:** 10.1093/femsle/fnad071

**Published:** 2023-07-21

**Authors:** Maria Chuvochina, Aaron J Mussig, Pierre-Alain Chaumeil, Adam Skarshewski, Christian Rinke, Donovan H Parks, Philip Hugenholtz

**Affiliations:** The University of Queensland, School of Chemistry and Molecular Biosciences, Australian Centre for Ecogenomics,, St Lucia QLD 4072, Brisbane, Australia; The University of Queensland, School of Chemistry and Molecular Biosciences, Australian Centre for Ecogenomics,, St Lucia QLD 4072, Brisbane, Australia; The University of Queensland, School of Chemistry and Molecular Biosciences, Australian Centre for Ecogenomics,, St Lucia QLD 4072, Brisbane, Australia; The University of Queensland, School of Chemistry and Molecular Biosciences, Australian Centre for Ecogenomics,, St Lucia QLD 4072, Brisbane, Australia; The University of Queensland, School of Chemistry and Molecular Biosciences, Australian Centre for Ecogenomics,, St Lucia QLD 4072, Brisbane, Australia; The University of Queensland, School of Chemistry and Molecular Biosciences, Australian Centre for Ecogenomics,, St Lucia QLD 4072, Brisbane, Australia; The University of Queensland, School of Chemistry and Molecular Biosciences, Australian Centre for Ecogenomics,, St Lucia QLD 4072, Brisbane, Australia

**Keywords:** Genome Taxonomy Database, ICNP, SeqCode, *Candidatus*

## Abstract

The Genome Taxonomy Database (GTDB) is a taxonomic framework that defines prokaryotic taxa as monophyletic groups in concatenated protein reference trees according to systematic criteria. This has resulted in a substantial number of changes to existing classifications (https://gtdb.ecogenomic.org). In the case of union of taxa, GTDB names were applied based on the priority of publication. The division of taxa or change in rank led to the formation of new Latin names above the rank of genus that were only made publicly available via the GTDB website without associated published taxonomic descriptions. This has sometimes led to confusion in the literature and databases. A number of the provisional GTDB names were later published in other studies, while many still lack authorships. To reduce further confusion, here we propose names and descriptions for 329 GTDB-defined prokaryotic taxa, 223 of which are suitable for validation under the International Code of Nomenclature of Prokaryotes (ICNP) and 49 under the Code of Nomenclature of Prokaryotes described from Sequence Data (SeqCode). For the latter, we designated 23 genomes as type material. An additional 57 taxa that do not currently satisfy the validation criteria of either code are proposed as *Candidatus*.

## Teaser

Provisional names earlier given to 329 GTDB-defined prokaryotic taxa are proposed according to the validation criteria of the International Code of Nomenclature of Prokaryotes and the SeqCode.

## Introduction

**Figure 1.**
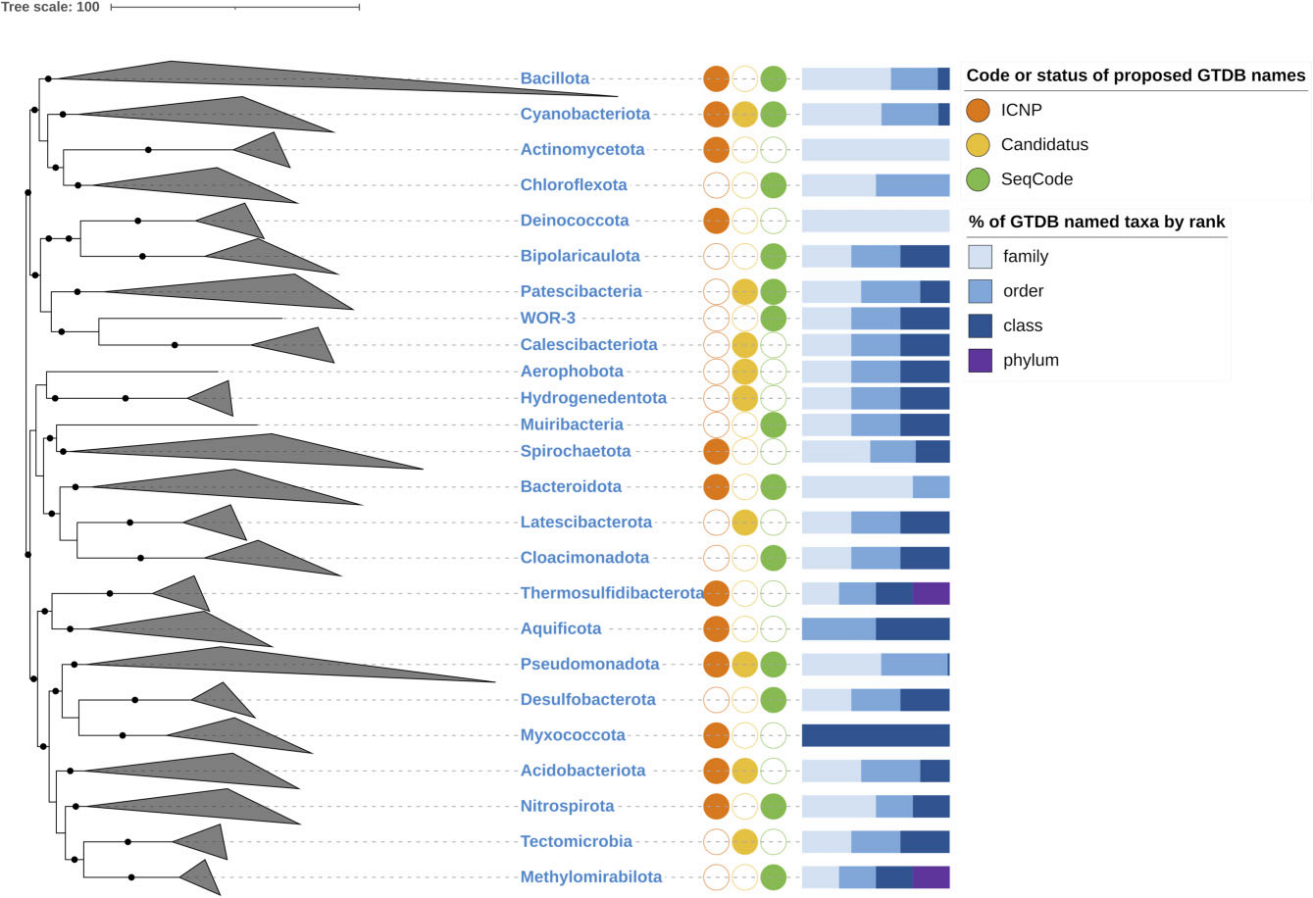
Phylogenetic position and phylum affiliation of GTDB-defined taxa with provisional Latin names proposed under the Prokaryotic code (ICNP), SeqCode and *Candidatus* status for the domain Bacteria. The relationships among the genera representing the types of the proposed names have been collapsed. The tree is inferred from the concatenated alignment of 120 ubiquitous, single-copy proteins using FastTree under the WAG model.

In November 2017, we introduced the Genome Taxonomy Database (GTDB)—a freely available web-based resource (https://gtdb.ecogenomic.org) providing classifications for bacterial and archaeal genomes (Parks et al. 2018; Rinke et al. 2021). The main objective of the GTDB is to provide a complete taxonomy (species to domain) for both cultured and uncultured taxa using a systematic genome-based approach. This includes an operational definition of species using average nucleotide identity (≥95% ANI; Parks et al. 2020) and normalization of taxonomic ranks above species using relative evolutionary divergence applied to a concatenated marker gene tree (Parks et al. 2018). The application of the GTDB taxonomic framework resulted in changes to the classification of 58% and 93% of bacterial and archaeal genomes, respectively (Parks et al. 2018; Rinke et al. 2021), that were typically accompanied by changes in nomenclature. These changes can be categorized according to the reclassification event—namely union, division, change in rank, or transfer of a species to a new genus (new combination). When named taxa of the same rank are united, one of the names is selected for the united taxon based on their priority. By contrast, a new name is formed when a taxon is divided, the rank of a taxon is changed, or a species is transferred to a new genus. Originally, such new names were only introduced via the GTDB website without associated taxonomic descriptions necessary for valid publication under the International Code of Nomenclature of Prokaryotes (ICNP). We have since discontinued this practice (Parks et al. 2022) as it has led to confusion arising from the subsequent publication of provisional GTDB Latin names by third parties (Sanford et al. 2021). For example, the effectively published family name ‘*Erysipelatoclostridiaceae*’ is listed in LPSN (List of Prokaryotic names with Standing in Nomenclature; Parte et al. 2020) under the authorship of Zakham et al. (2019), who neither defined nor described this taxon. By contrast, some of the provisional GTDB names were later proposed in effective publications by others. For example, the family *Desulfuribacillaceae*, order *Desulfuribacillales* and class *Desulfuribacillia* originally defined in GTDB as part of the phylum Bacillota/‘Firmicutes' (Parks et al. 2018) were proposed by Sorokin et al. ( 2020a,b,c) and their names validly published in the IJSEM Validation Lists. To prevent further confusion, here we have prepared an effective publication for most provisional GTDB Latin names above the rank of genus still in use in the GTDB taxonomy. In some cases, this entailed the proposal of genome sequences as type material under the SeqCode (see below). Note that due to continuous growth of the database and accompanying taxonomic revisions, some of the names proposed here may lack named parent taxa in later releases. Also note that the archaeal taxon names have already been proposed in a previous publication (Rinke et al. 2021), however, the proposals were in the supplementary materials, which typically does not qualify as an effective publication in the eyes of the International Journal of Systematic and Evolutionary Microbiology and the ICNP is currently ambiguous on this matter (Oren et al. 2023). Therefore, here we reproduce the archaeal taxa descriptions in the main body of the text for names based on ICNP validly published names of genera (Table 1) and for names based on SeqCode proposed type material as defined in Rinke et al. ( 2021). The phylogenetic evidence for the taxa bearing these names is provided in Fig. 1 and associated supplementary trees (Figures S2–S18, Supporting Information) based on GTDB R07-RS207.

**Table 1.** Description of GTDB-defined new higher taxa based on nomenclature types with names validly published under the ICNP.

Taxon name	Type^1^	Etymology	Properties and membership^2^
**Rank of Phylum (all proposed as phyl. nov.)**			
*Halobacteriota* (corrig. of ‘*Halobacterota*’)	Genus *Halobacterium* Elazari-Volcani 1957 (Approved Lists 1980)	Ha.lo.bac.te.ri.o'ta. N.L. neut. n. *Halobacterium* type genus of the phylum; -*ota* ending to denote a phylum; N.L. neut. pl. n. *Halobacteriota* the *Halobacterium* phylum	The phylum constitutes a monophyletic lineage as described and defined in Parks et al. (2018). The phylum contains the following classes: *Halobacteria, Archaeoglobi*, Methanocellia,^3^ ‘Methanomicrobia’, *Methanonatronarchaeia*, Methanosarcinia,^3^*Candidatus* Methanoliparia, *Candidatus* Syntropharchaeia^3^
*Thermoplasmatota*	Genus *Thermoplasma* Darland et al. 1970 (Approved lists 1980)	Ther.mo.plas.ma.to'ta. N.L. neut. n. *Thermoplasma* type genus of the phylum; -*ota* ending to denote a phylum; N.L. neut. pl. n. *Thermoplasmatota* the *Thermoplasma* phylum	The phylum constitutes a monophyletic lineage as described and defined in Parks et al. (2018). The phylum contains the following classes: *Thermoplasmata, Candidatus* Poseidoniia
*Thermosulfidibacterota*	Genus *Thermosulfidibacter* Nunoura et al. 2008	Ther.mo.sul.fi.di.bac.ter.o'ta. N.L. masc. n. *Thermosulfidibacter* type genus of the phylum; -*ota* ending to denote a phylum; N.L. neut. pl. n. *Thermosulfidibacterota* the *Thermosulfidibacter* phylum	The phylum constitutes a monophyletic lineage as described and defined in Parks et al. (2018). The phylum contains the class *Thermosulfidibacteria*^3^
**Rank of Class (all proposed as class. nov.)**			
*Alicyclobacillia*	Order *Alicyclobacillales*^3^	A.li.cy.clo.ba.cil'li.a. N.L. masc. n. *Alicyclobacillus* type genus of the type order of the class; -*ia* ending to denote a class; N.L. neut. pl. n. *Alicyclobacillia* the class of the order *Alicyclobacillales*	The class constitutes a monophyletic lineage as described and defined in Parks et al. (2018). The class contains the following orders: *Alicyclobacillales*,^3^*Kyrpidiales*,^3^*Tumebacillales*^3^
*Brachyspiria* (corrig. of ‘*Brachyspirae*’)	Order *Brachyspirales* Gupta et al. 2014	Bra.chy.spi'ri.a. N.L. fem. n. *Brachyspira* type genus of the type order of the class; -*ia* ending to denote a class; N.L. neut. pl. n. *Brachyspiria* the class of the order *Brachyspirales*	The class constitutes a monophyletic lineage as described and defined in Parks et al. (2018). The class contains the order *Brachyspirales*
*Bradymonadia* (corrig. of ‘*Bradimonadia*’)	Order *Bradymonadales* Wang et al. 2015	Bra.dy.mo.na'di.a. N.L. fem. n. *Bradymonas* type genus of the type order of the class; -*ia* ending to denote a class; N.L. neut. pl. n. *Bradymonadia* the class of the order *Bradymonadales*	The class constitutes a monophyletic lineage as described and defined in Parks et al. (2018). The class contains the order *Bradymonadales*
*Brevinematia*	Order *Brevinematales* Gupta et al. 2014	Bre.vi.ne.ma'ti.a. N.L. neut. n. *Brevinema* type genus of the type order of the class; -*ia* ending to denote a class; N.L. neut. pl. n. *Brevinematia* the class of the order *Brevinematales*	The class constitutes a monophyletic lineage as described and defined in Parks et al. (2018). The class contains the order *Brevinematales*
*Desulfitobacteriia*	Order *Desulfitobacteriales*^3^	De.sul.fi.to.bac.te.ri'i.a. N.L. neut. n. *Desulfitobacterium* type genus of the type order of the class; -*ia* ending to denote a class; N.L. neut. pl. n. *Desulfitobacteriia* the class of the order *Desulfitobacteriales*	The class constitutes a monophyletic lineage as described and defined in Parks et al. (2018). The class contains the following orders: *Desulfitobacteriales*,^3^ ‘Heliobacteriales’
*Desulfurobacteriia*	Order *Desulfurobacteriales* Gupta and Lali 2014	De.sul.fu.ro.bac.te.ri'i.a. N.L. neut. n. *Desulfurobacterium* type genus of the type order of the class; -*ia* ending to denote a class; N.L. neut. pl. n. *Desulfurobacteriia* the class of the order *Desulfurobacteriales*	The class constitutes a monophyletic lineage as described and defined in Parks et al. (2018). The class contains the order *Desulfurobacteriales*
*Desulfotomaculia*	Order *Desulfotomaculales*^3^	De.sul.fo.to.ma.cu'li.a. N.L. neut. n. *Desulfotomaculum* type genus of the type order of the class; -*ia* ending to denote a class; N.L. neut. pl. n. *Desulfotomaculia* the class of the order *Desulfotomaculales*	The class constitutes a monophyletic lineage as described and defined in Parks et al. (2018). The class contains the following orders: *Desulfotomaculales*,^3^*Ammonificales*,^3^*Carboxydothermales*^3^
*Leptospiria* (corrig. of ‘*Leptospirae*’)	Order *Leptospirales* Gupta et al. 2014	Lep.to.spi'ri.a. N.L. fem. n. *Leptospira* type genus of the type order of the class; -*ia* ending to denote a class; N.L. neut. pl. n. *Leptospiria* the class of the order *Leptospirales*	The class constitutes a monophyletic lineage as described and defined in Parks et al. (2018). The class contains the following orders: *Leptospirales, Turneriellales*^3^
*Leptospirillia*	Order *Leptospirillales*^3^	Lep.to.spi.ril'li.a. N.L. neut. n. *Leptospirillum* type genus of the type order of the class; -*ia* ending to denote a class; N.L. neut. pl. n. *Leptospirillia* the class of the order *Leptospirillales*	The class constitutes a monophyletic lineage as described and defined in Parks et al. (2018). The class contains the order *Leptospirillales*^3^
*Magnetococcia*	Order *Magnetococcales* Bazylinski et al. 2013	Mag.ne.to.coc'ci.a. N.L. masc. n. *Magnetococcus* type genus of the type order of the class; -*ia* ending to denote a class; N.L. neut. pl. n. *Magnetococcia* the class of the order *Magnetococcales*	The class constitutes a monophyletic lineage as described and defined in Parks et al. (2018). The class contains the order *Magnetococcales*
*Methanocellia*	Order *Methanocellales* Sakai et al. 2008	Me.tha.no.cel'li.a. N.L. fem. n. *Methanocella* type genus of the type order of the class; -*ia* ending to denote a class; N.L. neut. pl. n. *Methanocellia* the class of the order *Methanocellales*	The class constitutes a monophyletic lineage as described and defined in Parks et al. (2018). The class contains the order *Methanocellales*
*Methanosarcinia*	Order *Methanosarcinales* Boone et al. 2002	Me.tha.no.sar.ci'ni.a. N.L. fem. n. *Methanosarcina* type genus of the type order of the class; -*ia* ending to denote a class; N.L. neut. pl. n. *Methanosarcinia* the class of the order *Methanosarcinales*	The class constitutes a monophyletic lineage as described and defined in Parks et al. (2018). The class contains the following orders: *Methanosarcinales, Methanotrichales*^3^
*Natranaerobiia*	Order *Natranaerobiales* Mesbah et al. 2007	Natr.an.a.e.ro.bi’.i.a. N.L. masc. n. *Natranaerobius* type genus of the type order of the class; -*ia* ending to denote a class; N.L. neut. pl. n. *Natranaerobiia* the class of the order *Natranaerobiales*	The class constitutes a monophyletic lineage as described and defined in Parks et al. (2018). The class contains the order *Natranaerobiales*
*Peptococcia*	Order *Peptococcales*^3^	Pep.to.coc'ci.a. N.L. masc. n. *Peptococcus* type genus of the type order of the class; -*ia* ending to denote a class; N.L. neut. pl. n. *Peptococcia* the class of the order *Peptococcales*	The class constitutes a monophyletic lineage as described and defined in Parks et al. (2018). The class contains the order *Peptococcales*^3^
*Sulfobacillia*	Order *Sulfobacillales*^3^	Sul.fo.ba.cil'li.a. N.L. masc. n. *Sulfobacillus* type genus of the type order of the class; -*ia* ending to denote a class; N.L. neut. pl. n. *Sulfobacillia* the class of the order *Sulfobacillales*	The class constitutes a monophyletic lineage as described and defined in Parks et al. (2018). The class contains the order *Sulfobacillales*^3^
*Symbiobacteriia*	Order *Symbiobacteriales*^3^	Sym.bi.o.bac.te.ri'i.a. N.L. neut. n. *Symbiobacterium* type genus of the type order of the class; -*ia* ending to denote a class; N.L. neut. pl. n. *Symbiobacteriia* the class of the order *Symbiobacteriales*	The class constitutes a monophyletic lineage as described and defined in Parks et al. (2018). The class contains the order *Symbiobacteriales*^3^
*Syntrophomonadia*	Order *Syntrophomonadales*^3^	Syn.tro.pho.mo.na'di.a. N.L. fem. n. *Syntrophomonas* type genus of the type order of the class; -*ia* ending to denote a class; N.L. neut. pl. n. *Syntrophomonadia* the class of the order *Syntrophomonadales*	The class constitutes a monophyletic lineage as described and defined in Parks et al. (2018). The class contains the following orders: *Syntrophomonadales*,^3^*Thermacetogeniales*^3^
*Thermaerobacteria*	Order *Thermaerobacterales*^3^	Therm.a.e.ro.bac.te'ri.a. N.L. masc. n. *Thermaerobacter* type genus of the type order of the class; -*ia* ending to denote a class; N.L. neut. pl. n. *Thermaerobacteria* the class of the order *Thermaerobacterales*	The class constitutes a monophyletic lineage as described and defined in Parks et al. (2018). The class contains the order *Thermaerobacterales*^3^
*Thermincolia*	Order *Thermincolales*^3^	Therm.in.co'li.a. N.L. fem. n. *Thermincola* type genus of the type order of the class; -*ia* ending to denote a class; N.L. neut. pl. n. *Thermincolia* the class of the order *Thermincolales*	The class constitutes a monophyletic lineage as described and defined in Parks et al. (2018). The class contains the following orders: *Thermincolales*,^3^*Carboxydocellales*^3^
*Thermosediminibacteria*	Order *Thermosediminibacterales* Zhang et al. 2019	Ther.mo.se.di.mi.ni.bac.te'ri.a. N.L. masc. n. *Thermosediminibacter* type genus of the type order of the class; -*ia* ending to denote a class; N.L. neut. pl. n. *Thermosediminibacteria* the class of the order *Thermosediminibacterales*	The class constitutes a monophyletic lineage as described and defined in Parks et al. (2018). The class contains the order *Thermosediminibacterales*
*Thermosulfidibacteria*	Order *Thermosulfidibacterales*^3^	Ther.mo.sulfi.di.bac.te'ri.a. N.L. masc. n. *Thermosulfidibacter* type genus of the type order of the class; -*ia* ending to denote a class; N.L. neut. pl. n. *Thermosulfidibacteria* the class of the order *Thermosulfidibacterales*	The class constitutes a monophyletic lineage as described and defined in Parks et al. (2018). The class contains the order *Thermosulfidibacterales*^3^
*Vampirovibrionia*	Order *Vampirovibrionales*^3^	Vam.pi.ro.vi.bri.o'ni.a. N.L. masc. n. *Vampirovibrio* type genus of the type order of the class; -*ia* ending to denote a class; N.L. neut. pl. n. *Vampirovibrionia* the class of the order *Vampirovibrionales*	The class constitutes a monophyletic lineage as described and defined in Parks et al. (2018). The class contains the following orders: *Vampirovibrionales*,^3^*Candidatus* Caenarcaniphilales, *Candidatus* Gastranaerophilales, *Candidatus* Obscuribacterales peptococcia
**Rank of Order (all proposed as ord. nov.)**			
*Acetivibrionales*	Genus *Acetivibrio* Patel et al. 1980	A.ce.ti.vib.ri.o.na'les. N.L. masc. n. *Acetivibrio* type genus of the order; -*ales* ending to denote an order; N.L. fem. pl. n. *Acetivibrionales* the *Acetivibrio* order	The order constitutes a monophyletic lineage as described and defined in Parks et al. (2018). The order contains the family *Acetivibrionaceae*^3^
*Acetobacterales*	Genus *Acetobacter* Beijerinck 1898 (Approved Lists 1980)	A.ce.to.bac.te.ra'les. N.L. masc. n. *Acetobacter* type genus of the order; -*ales* ending to denote an order; N.L. fem. pl. n. *Acetobacterales* the *Acetobacter* order	The order constitutes a monophyletic lineage as described and defined in Parks et al. (2018). The order contains the family *Acetobacteraceae*
*Alicyclobacillales*	Genus *Alicyclobacillus* Wisotzkey et al. 1992	A.li.cy.clo.ba.cil.la'les.N.L. masc. n. *Alicyclobacillus* type genus of the order; -*ales* ending to denote an order; N.L. fem. pl. n. *Alicyclobacillales* the *Alicyclobacillus* order	The order constitutes a monophyletic lineage as described and defined in Parks et al. (2018). The order contains the family *Alicyclobacillaceae*
*Ammonificales* (corrig. of ‘*Ammonifexales*’)	Genus *Ammonifex* Huber and Stetter 1996	*Am.mo.ni.fi.ca'les*. N.L. masc. n. *Ammonifex* type genus of the order; -*ales* ending to denote an order; N.L. fem. pl. n. *Ammonificales* the *Ammonifex* order	The order constitutes a monophyletic lineage as described and defined in Parks et al. (2018). The order contains the following families: *Ammonificaceae*,^3^*Candidatus* Desulforudaceae^3^
*Anaeromusales*	Genus *Anaeromusa* Baena et al. 1999	*An.a.e.ro.mu.sa'les*. N.L. fem. n. *Anaeromusa* type genus of the order; -*ales* ending to denote an order; N.L. fem. pl. n. *Anaeromusales* the *Anaeromusa* order	The order constitutes a monophyletic lineage as described and defined in Parks et al. (2018). The order contains the family *Anaeromusaceae*^3^
*Aneurinibacillales*	Genus *Aneurinibacillus* Shida et al. 1996	A.neu.ri.ni.ba.cil.la'les. N.L. masc. n. *Aneurinibacillus* type genus of the order; -*ales* ending to denote an order; N.L. fem. pl. n. *Aneurinibacillales* the *Aneurinibacillus* order	The order constitutes a monophyletic lineage as described and defined in Parks et al. (2018). The order contains the family *Aneurinibacillaceae*^3^
*Azospirillales*	Genus *Azospirillum* Tarrand et al. 1979 (Approved Lists 1980)	Azo.spi.ril.la'les. N.L. neut. n. *Azospirillum* type genus of the order; -*ales* ending to denote an order; N.L. fem. pl. n. *Azospirillales* the *Azospirillum* order	The order constitutes a monophyletic lineage as described and defined in Parks et al. (2018). The order contains the family *Azospirillaceae*
*Borreliales*	Genus *Borrelia* Swellengrebel 1907 (Approved Lists 1980)	Bor.re.li.a'les. N.L. fem. n. *Borrelia* type genus of the order; -*ales* ending to denote an order; N.L. fem. pl. n. *Borreliales* the *Borrelia* order	The order constitutes a monophyletic lineage as described and defined in Parks et al. (2018). The order contains the family *Borreliaceae*
*Brevibacillales*	Genus *Brevibacillus* Shida et al. 1996	Bre.vi.ba.cil.la'les. N.L. masc. n. *Brevibacillus* type genus of the order; -*ales* ending to denote an order; N.L. fem. pl. n. *Brevibacillales* the *Brevibacillus* order	The order constitutes a monophyletic lineage as described and defined in Parks et al. (2018). The order contains the family *Brevibacillaceae*^3^
*Caldalkalibacillales*	Genus *Caldalkalibacillus* Xue et al. 2006	Cald.al.ka.li.ba.cil.la'les. N.L. masc. n. *Caldalkalibacillus* type genus of the order; -*ales* ending to denote an order; N.L. fem. pl. n. *Caldalkalibacillales* the *Caldalkalibacillus* order	The order constitutes a monophyletic lineage as described and defined in Parks et al. (2018). The order contains the family *Caldalkalibacillaceae*^3^
*Calderihabitantales*	Genus *Calderihabitans* Yoneda et al. 2013	Cal.de.ri.ha.bi.tan.ta'les. N.L. masc. n. *Calderihabitans* type genus of the order; -*ales* ending to denote an order; N.L. fem. pl. n. *Calderihabitantales* the *Calderihabitans* order	The order constitutes a monophyletic lineage as described and defined in Parks et al. (2018). The order contains the family *Calderihabitantaceae*
*Caldicellulosiruptorales*	Genus *Caldicellulosiruptor* Rainey et al. 1995	*Cal.di.cel.lu.lo.si.rup.to.ra'les*. N.L. masc. n. *Caldicellulosiruptor* type genus of the order; -*ales* ending to denote an order; N.L. fem. pl. n. *Caldicellulosiruptorales* the *Caldicellulosiruptor* order	The order constitutes a monophyletic lineage as described and defined in Parks et al. (2018). The order contains the family *Caldicellulosiruptoraceae*^3^
*Caldicoprobacterales*	Genus *Caldicoprobacter* Yokoyama et al. 2010	*Cal.di.co.pro.bac.te.ra'les*. N.L. masc. n. *Caldicoprobacter* type genus of the order; -*ales* ending to denote an order; N.L. fem. pl. n. *Caldicoprobacterales* the *Caldicoprobacter* order	The order constitutes a monophyletic lineage as described and defined in Parks et al. (2018). The order contains the following families: *Caldicoprobacteraceae, Xylanivirgaceae*
*Calditerricolales*	Genus *Calditerricola* Moriya et al. 2011	Cal.di.ter.ri.co.la'les. N.L. masc. n. *Calditerricola* type genus of the order; -*ales* ending to denote an order; N.L. fem. pl. n. *Calditerricolales* the *Calditerricola* order	The order constitutes a monophyletic lineage as described and defined in Parks et al. (2018). The order contains the family *Calditerricolaceae*^3^
*Carboxydocellales*	Genus *Carboxydocella* Sokolova et al. 2002	Car.bo.xy.do.cel.la'les. N.L. fem. n. *Carboxydocella* type genus of the order; -*ales* ending to denote an order; N.L. fem. pl. n. *Carboxydocellales* the *Carboxydocella* order	The order constitutes a monophyletic lineage as described and defined in Parks et al. (2018). The order contains the family *Carboxydocellaceae*^3^
*Carboxydothermales*	Genus *Carboxydothermus* Svetlichny et al. 1991	*Car.bo.xy.do.ther.ma'les*. N.L. masc. n. *Carboxydothermus* type genus of the order; -*ales* ending to denote an order; N.L. fem. pl. n. *Carboxydothermales* the *Carboxydothermus* order	The order constitutes a monophyletic lineage as described and defined in Parks et al. (2018). The order contains the family *Carboxydothermaceae*^3^
*Christensenellales*	Genus *Christensenella* Morotomi et al. 2012	Chris.ten.se.nel.la'les. N.L. fem. n. *Christensenella* type genus of the order; -*ales* ending to denote an order; N.L. fem. pl. n. *Christensenellales* the *Christensenella* order	The order constitutes a monophyletic lineage as described and defined in Parks et al. (2018). The order contains the following families: *Christensenellaceae, Candidatus* Borkfalkiaceae
*Coxiellales*	Genus *Coxiella* Philip 1943 1948) (Approved Lists 1980)	*Co.xi.el.la'les*. N.L. fem. n. *Coxiella* type genus of the order; -*ales* ending to denote an order; N.L. fem. pl. n. *Coxiellales* the *Coxiella* order	The order constitutes a monophyletic lineage as described and defined in Parks et al. (2018). The order contains the family *Coxiellaceae*
*Desulfitibacterales*	Genus *Desulfitibacter* Nielsen et al. 2006	De.sul.fi.ti.bac.te.ra'les. N.L. masc. n. *Desulfitibacter* type genus of the order; -*ales* ending to denote an order; N.L. fem. pl. n. *Desulfitibacterales* the *Desulfitibacter* order	The order constitutes a monophyletic lineage as described and defined in Parks et al. (2018). The order contains the family *Desulfitibacteraceae*
*Desulfitobacteriales*	Genus *Desulfitobacterium* Utkin et al. 1994	De.sul.fi.to.bac.te.ri.a'les. N.L. neut. n. *Desulfitobacterium* type genus of the order; -*ales* ending to denote an order; N.L. fem. pl. n. *Desulfitobacteriales* the *Desulfitobacterium* order	The order constitutes a monophyletic lineage as described and defined in Parks et al. (2018). The order contains the family *Desulfitobacteriaceae*
*Desulfotomaculales*	Genus *Desulfotomaculum* Campbell and Postgate 1965 (Approved Lists 1980)	*De.sul.fo.to.ma.cu.la'les*. N.L. neut. n. *Desulfotomaculum* type genus of the order; -*ales* ending to denote an order; N.L. fem. pl. n. *Desulfotomaculales* the *Desulfotomaculum* order	The order constitutes a monophyletic lineage as described and defined in Parks et al. (2018). The order contains the following families: *Desulfotomaculaceae, Desulfallaceae, Desulfocucumaceae, ‘Desulfofarciminaceae’, Desulfovirgulaceae*,^3^*Desulfurisporaceae, Pelotomaculaceae*^3^
*Diplorickettsiales*	Genus *Diplorickettsia* Mediannikov et al. 2011	Di.plo.ric.kett.si.a'les. N.L. fem. n. *Diplorickettsia* type genus of the order; -*ales* ending to denote an order; N.L. fem. pl. n. *Diplorickettsiales* the *Diplorickettsia* order	The order constitutes a monophyletic lineage as described and defined in Parks et al. (2018). The order contains the family *Diplorickettsiaceae*^3^
*Dongiales*	Genus *Dongia* Liu et al. 2010	Dong.i.a'les. N.L. fem. n. *Dongia* type genus of the order; -*ales* ending to denote an order; N.L. fem. pl. n. *Dongiales* the *Dongia* order	The order constitutes a monophyletic lineage as described and defined in Parks et al. (2018). The order contains the family *Dongiaceae*
*Ectothiorhodospirales*	Genus *Ectothiorhodospira* Pelsh 1936 (Approved Lists 1980)	Ec.to.thi.o.rho.do.spi.ra'les. N.L. fem. n. *Ectothiorhodospira* type genus of the order; -*ales* ending to denote an order; N.L. fem. pl. n. *Ectothiorhodospirales* the *Ectothiorhodospira* order	The order constitutes a monophyletic lineage as described and defined in Parks et al. (2018). The order contains the following families: *Ectothiorhodospiraceae, Acidihalobacteraceae*,^3^*Thioalkalivibrionaceae*^3^
*Elainellales*	Genus *Elainella* Jahodářová et al. 2018	E.lai.nel.la'les. N.L. fem. n. *Elainella* type genus of the order; -*ales* ending to denote an order; N.L. fem. pl. n. *Elainellales* the *Elainella* order	The order constitutes a monophyletic lineage as described and defined in Parks et al. (2018). The order contains the family *Elainellalaceae*^3^
*Elsterales*	Genus *Elstera* Rahalkar et al. 2012	El.ste.ra'les. N.L. fem. n. *Elstera* type genus of the order; -*ales* ending to denote an order; N.L. fem. pl. n. *Elsterales* the *Elstera* order	The order constitutes a monophyletic lineage as described and defined in Parks et al. (2018). The order contains the family *Elsteraceae*^3^
*Exiguobacteriales* (corrig. of ‘*Exiguobacterales*’)	Genus *Exiguobacterium* Collins et al. 1984	Ex.i.gu.o.bac.te.ri.a'les. N.L. neut. n. *Exiguobacterium* type genus of the order; -*ales* ending to denote an order; N.L. fem. pl. n. *Exiguobacteriales* the *Exiguobacterium* order	The order constitutes a monophyletic lineage as described and defined in Parks et al. (2018). The order contains the family *Exiguobacteriaceae*^3^
*Ferrovibrionales*	Genus *Ferrovibrio* Sorokina et al. 2013	Fer.ro.vib.ri.o.na'les. N.L. masc. n. *Ferrovibrio* type genus of the order; -*ales* ending to denote an order; N.L. fem. pl. n. *Ferrovibrionales* the *Ferrovibrio* order	The order constitutes a monophyletic lineage as described and defined in Parks et al. (2018). The order contains the family *Ferrovibrionaceae*^3^
*Francisellales*	Genus *Francisella* Dorofeev 1947 (Approved Lists 1980)	Fran.ci.sel.la'les. N.L. fem. n. *Francisella* type genus of the order; -*ales* ending to denote an order; N.L. fem. pl. n. *Francisellales* the *Francisella* order	The order constitutes a monophyletic lineage as described and defined in Parks et al. (2018). The order contains the family *Francisellaceae*
*Geminicoccales*	Genus *Geminicoccus* Foesel et al. 2008	Ge.mi.ni.coc.ca'les. N.L. masc. n. *Geminicoccus* type genus of the order; -*ales* ending to denote an order; N.L. fem. pl. n. *Geminicoccales* the *Geminicoccus* order	The order constitutes a monophyletic lineage as described and defined in Parks et al. (2018). The order contains the family *Geminicoccaceae*
*Granulosicoccales*	Genus *Granulosicoccus* Lee et al. 2008	Gra.nu.lo.si.coc.ca'les. N.L. masc. n. *Granulosicoccus* type genus of the order; -*ales* ending to denote an order; N.L. fem. pl. n. *Granulosicoccales* the *Granulosicoccus* order	The order constitutes a monophyletic lineage as described and defined in Parks et al. (2018). The order contains the family *Granulosicoccaceae*
*Halobacteroidales*	Genus *Halobacteroides* Oren et al. 1984	*Ha.lo.bac.te.ro.i.da'les*. N.L. masc. n. *Halobacteroides* type genus of the order; -*ales* ending to denote an order; N.L. fem. pl. n. *Halobacteroidales* the *Halobacteroides* order	The order constitutes a monophyletic lineage as described and defined in Parks et al. (2018). The order contains the following families: *Halobacteroidaceae, Acetohalobiaceae*^3^
*Halothiobacillales*	Genus *Halothiobacillus* Kelly and Wood 2000	Ha.lo.thi.o.ba.cil.la'les. N.L. masc. n. *Halothiobacillus* type genus of the order; -*ales* ending to denote an order; N.L. fem. pl. n. *Halothiobacillales* the *Halothiobacillus* order	The order constitutes a monophyletic lineage as described and defined in Parks et al. (2018). The order contains the family *Halothiobacillaceae*
*Hydrogenothermales*	Genus *Hydrogenothermus* Stöhr et al. 2001	Hyd.ro.ge.no.ther.ma'les. N.L. masc. n. *Hydrogenothermus* type genus of the order; -*ales* ending to denote an order; N.L. fem. pl. n. *Hydrogenothermales* the *Hydrogenothermus* order	The order constitutes a monophyletic lineage as described and defined in Parks et al. (2018). The order contains the family *Hydrogenothermaceae*
*Kyrpidiales*	Genus *Kyrpidia* Klenk et al. 2012	Kyr.pi.di.a'les. N.L. fem. n. *Kyrpidia* type genus of the order; -*ales* ending to denote an order; N.L. fem. pl. n. *Kyrpidiales* the *Kyrpidia* order	The order constitutes a monophyletic lineage as described and defined in Parks et al. (2018). The order contains the family *Kyrpidiaceae*^3^
*Lachnospirales*	Genus *Lachnospira* Bryant and Small 1956 (Approved Lists 1980)	Lach.no.spi.ra'les. N.L. fem. n. *Lachnospira* type genus of the order; -*ales* ending to denote an order; N.L. fem. pl. n. *Lachnospirales* the *Lachnospira* order	The order constitutes a monophyletic lineage as described and defined in Parks et al. (2018). The order contains the following families: *Lachnospiraceae, Cellulosilyticaceae*,^3^*Defluviitaleaceae, Anaerotignaceae*,^3^*Vallitaleaceae*
*Leptolyngbyales*	Genus *Leptolyngbya* Anagnostidis and Komárek 1988	Lep.to.lyng.by.a'les. N.L. fem. n. *Leptolyngbya* type genus of the order; -*ales* ending to denote an order; N.L. fem. pl. n. *Neosynechococcales* the *Leptolyngbya* order	The order constitutes a monophyletic lineage as described and defined in Parks et al. (2018). The order contains the family *Leptolyngbyaceae*
*Leptospirillales*	Genus *Leptospirillum* (*ex* Markosyan 1972) Hippe 2000	Lep.to.spi.ril.la'les. N.L. neut. n. *Leptospirillum* type genus of the order; -*ales* ending to denote an order; N.L. fem. pl. n. *Leptospirillales* the *Leptospirillum* order	The order constitutes a monophyletic lineage as described and defined in Parks et al. (2018). The order contains the family *Leptospirillaceae*^3^
*Lutisporales*	Genus *Lutispora* Shiratori et al. 2008	Lu.ti.spo.ra'les. N.L. fem. n. *Lutispora* type genus of the order; -*ales* ending to denote an order; N.L. fem. pl. n. *Lutisporales* the *Lutispora* order	The order constitutes a monophyletic lineage as described and defined in Parks et al. (2018). The order contains the family *Lutisporaceae*^3^
*Mahellales*	Genus *Mahella* Bonilla Salinas et al. 2004	Ma.hel.la'les. N.L. fem. n. *Mahella* type genus of the order; -*ales* ending to denote an order; N.L. fem. pl. n. *Mahellales* the *Mahella* order	The order constitutes a monophyletic lineage as described and defined in Parks et al. (2018). The order contains the family *Mahellaceae*^3^
*Monoglobales*	Genus *Monoglobus* Kim et al. 2017	Mo.no.glo.ba'les. N.L. masc. n. *Monoglobus* type genus of the order; -*ales* ending to denote an order; N.L. fem. pl. n. *Monoglobales* the *Monoglobus* order	The order constitutes a monophyletic lineage as described and defined in Parks et al. (2018). The order contains the family *Monoglobaceae*^3^
*Neosynechococcales*	Genus *Neosynechococcus* Dvořák et al. 2014	Ne.o.syn.e.cho.coc.ca'les. N.L. masc. n. *Neosynechococcus* type genus of the order; -*ales* ending to denote an order; N.L. fem. pl. n. *Neosynechococcales* the *Neosynechococcus* order	The order constitutes a monophyletic lineage as described and defined in Parks et al. (2018). The order contains the family *Neosynechococcaceae*
*Nitrococcales*	Genus *Nitrococcus* Watson and Waterbury 1971 (Approved Lists 1980)	Ni.tro.coc.ca'les. N.L. masc. n. *Nitrococcus* type genus of the order; -*ales* ending to denote an order; N.L. fem. pl. n. *Nitrococcales* the *Nitrococcus* order	The order constitutes a monophyletic lineage as described and defined in Parks et al. (2018). The order contains the following families: *Nitrococcaceae*,^3^*Halorhodospiraceae*,^3^*Aquisalimonadaceae*^3^
*Nitrosococcales*	Genus *Nitrosococcus* Winogradsky 1892 (Approved Lists 1980)	Ni.tro.so.coc.ca'les. N.L. masc. n. *Nitrosococcus* type genus of the order; -*ales* ending to denote an order; N.L. fem. pl. n. *Nitrosococcales* the *Nitrosococcus* order	The order constitutes a monophyletic lineage as described and defined in Parks et al. (2018). The order contains the following families: *Nitrosococcaceae*,^3^*Methylophagaceae*^3^
*Oceanibaculales*	Genus *Oceanibaculum* Lai et al. 2009	O.ce.a.ni.ba.cu.la'les. N.L. neut. n. *Oceanibaculum* type genus of the order; -*ales* ending to denote an order; N.L. fem. pl. n. *Oceanibaculales* the *Oceanibaculum* order	The order constitutes a monophyletic lineage as described and defined in Parks et al. (2018). The order contains the family *Oceanibaculaceae*
*Paenibacillales*	Genus *Paenibacillus* Ash et al. 1994	*Pae.ni.ba.cil.la'les*. N.L. masc. n. *Paenibacillus* type genus of the order; -*ales* ending to denote an order; N.L. fem. pl. n. *Paenibacillales* the *Paenibacillus* order	The order constitutes a monophyletic lineage as described and defined in Parks et al. (2018). The order contains the family *Paenibacillaceae*
*Parvibaculales*	Genus *Parvibaculum* Schleheck et al. 2004	Par.vi.ba.cu.la'les. N.L. neut. n. *Parvibaculum* type genus of the order; -*ales* ending to denote an order; N.L. fem. pl. n. *Parvibaculales* the *Parvibaculum* order	The order constitutes a monophyletic lineage as described and defined in Parks et al. (2018). The order contains the following families: *Parvibaculaceae, Candidatus* Phaeomarinobacteraceae
*Peptococcales*	Genus *Peptococcus* Kluyver and van Niel 1936 (Approved Lists 1980)	Pep.to.coc.ca'les. N.L. masc. n. *Peptococcus* type genus of the order; -*ales* ending to denote an order; N.L. fem. pl. n. *Peptococcales* the *Peptococcus* order	The order constitutes a monophyletic lineage as described and defined in Parks et al. (2018). The order contains the following families: *Peptococcaceae, Desulfonisporaceae*^3^
*Peptostreptococcales*	Genus *Peptostreptococcus* Kluyver and van Niel 1936 (Approved Lists 1980)	Pep.to.strep.to.coc.ca'les. N.L. masc. n. *Peptostreptococcus* type genus of the order; -*ales* ending to denote an order; N.L. fem. pl. n. *Peptostreptococcales* the *Peptostreptococcus* order	The order constitutes a monophyletic lineage as described and defined in Parks et al. (2018). The order contains the following families: *Peptostreptococcaceae, Acidaminobacteraceae*,^3^*Anaerovoracaceae*,^3^*Caminicellaceae*,^3^*Filifactoraceae*,^3^*Natronincolaceae*,^3^*Thermotaleaceae*,^3^*Tindalliaceae*^3^
*Phormidesmales* (corrig. of ‘*Phormidesmiales’*)	Genus *Phormidesmis* Turicchia et al. 2009	Phor.mi.des.ma'les. N.L. fem. n. *Phormidesmis* type genus of the order; -*ales* ending to denote an order; N.L. fem. pl. n. *Phormidesmales* the *Phormidesmis* order	The order constitutes a monophyletic lineage as described and defined in Parks et al. (2018). The order contains the family *Phormidesmaceae*^3^
*Piscirickettsiales*	Genus *Piscirickettsia* Fryer et al. 1992	Pis.ci.ric.kett.si.a'les. N.L. fem. n. *Piscirickettsia* type genus of the order; -*ales* ending to denote an order; N.L. fem. pl. n. *Piscirickettsiales* the *Piscirickettsia* order	The order constitutes a monophyletic lineage as described and defined in Parks et al. (2018). The order contains the family *Piscirickettsiaceae*
*Propionisporales*	Genus *Propionispora* Biebl et al. 2001	Pro.pi.o.ni.spo.ra'les. N.L. fem. n. *Propionispora* type genus of the order; -*ales* ending to denote an order; N.L. fem. pl. n. *Propionisporales* the *Propionispora* order	The order constitutes a monophyletic lineage as described and defined in Parks et al. (2018). The order contains the family *Propionisporaceae*^3^
*Pyrinomonadales*	Genus *Pyrinomonas* Crowe et al. 2014	Py.ri.no.mo.na.da'les. N.L. fem. n. *Pyrinomonas* type genus of the order; -*ales* ending to denote an order; N.L. fem. pl. n. *Pyrinomonadales* the *Pyrinomonas* order	The order constitutes a monophyletic lineage as described and defined in Parks et al. (2018). The order contains the family *Pyrinomonadaceae*
*Reyranellales*	Genus *Reyranella* Pagnier et al. 2011	Rey.ra.nel.la'les. N.L. fem. n. *Reyranella* type genus of the order; -*ales* ending to denote an order; N.L. fem. pl. n. *Reyranellales* the *Reyranella* order	The order constitutes a monophyletic lineage as described and defined in Parks et al. (2018). The order contains the family *Reyranellaceae*
*Sphaerochaetales*	Genus *Sphaerochaeta* Ritalahti et al. 2012	Sphae.ro.chae.ta'les. N.L. fem. n. *Sphaerochaeta* type genus of the order; -*ales* ending to denote an order; N.L. fem. pl. n. *Sphaerochaetales* the *Sphaerochaeta* order	The order constitutes a monophyletic lineage as described and defined in Parks et al. (2018). The order contains the family *Sphaerochaetaceae*
*Staphylococcales*	Genus *Staphylococcus* Rosenbach 1884 (Approved Lists 1980)	Sta.phy.lo.coc.ca'les. N.L. masc. n. *Staphylococcus* type genus of the order; -*ales* ending to denote an order; N.L. fem. pl. n. *Staphylococcales* the *Staphylococcus* order	The order constitutes a monophyletic lineage as described and defined in Parks et al. (2018). The order contains the following families: *Staphylococcaceae, Gemellaceae*,^3^*Salinicoccaceae*^3^
*Steroidobacterales*	Genus *Steroidobacter* Fahrbach et al. 2008	Ste.ro.i.do.bac.te.ra'les. N.L. masc. n. *Steroidobacter* type genus of the order; -*ales* ending to denote an order; N.L. fem. pl. n. *Steroidobacterales* the *Steroidobacter* order	The order constitutes a monophyletic lineage as described and defined in Parks et al. (2018). The order contains the family *Steroidobacteraceae*
*Sulfobacillales*	Genus *Sulfobacillus* Golovacheva and Karavaiko 1991	Sul.fo.ba.cil.la'les. N.L. masc. n. *Sulfobacillus* type genus of the order; -*ales* ending to denote an order; N.L. fem. pl. n. *Sulfobacillales* the *Sulfobacillus* order	The order constitutes a monophyletic lineage as described and defined in Parks et al. (2018). The order contains the family *Sulfobacillaceae*^3^
*Symbiobacteriales*	Genus *Symbiobacterium* Ohno et al. 2000	Sym.bi.o.bac.te.ri.a'les. N.L. neut. n. *Symbiobacterium* type genus of the order; -*ales* ending to denote an order; N.L. fem. pl. n. *Symbiobacteriales* the *Symbiobacterium* order	The order constitutes a monophyletic lineage as described and defined in Parks et al. (2018). The order contains the family *Symbiobacteriaceae*
*Syntrophomonadales*	Genus *Syntrophomonas* McInerney et al. 1982	Syn.tro.pho.mo.na.da'les. N.L. fem. n. *Syntrophomonas* type genus of the order; -*ales* ending to denote an order; N.L. fem. pl. n. *Syntrophomonadales* the *Syntrophomonas* order	The order constitutes a monophyletic lineage as described and defined in Parks et al. (2018). The order contains the following families: *Syntrophomonadaceae, Syntrophothermaceae*^3^
*Tepidibacillales*	Genus *Tepidibacillus* Slobodkina et al. 2014	Te.pi.di.ba.cil.la'les. N.L. masc. n. *Tepidibacillus* type genus of the order; -*ales* ending to denote an order; N.L. fem. pl. n. *Tepidibacillales* the *Tepidibacillus* order	The order constitutes a monophyletic lineage as described and defined in Parks et al. (2018). The order contains the family *Tepidibacillaceae*^3^
*Thalassobaculales*	Genus *Thalassobaculum* Zhang et al. 2008	Tha.las.so.ba.cu.la'les. N.L. neut. n. *Thalassobaculum* type genus of the order; -*ales* ending to denote an order; N.L. fem. pl. n. *Thalassobaculales* the *Thalassobaculum* order	The order constitutes a monophyletic lineage as described and defined in Parks et al. (2018). The order contains the following families: *Thalassobaculaceae, Oceanibaculaceae*,^3^*Nisaeaceae*^3^
*Thermacetogeniales*	Genus *Thermacetogenium* Hattori et al. 2000	Therm.a.ce.to.ge.ni.a'les. N.L. neut. n. *Thermacetogenium* type genus of the order; -*ales* ending to denote an order; N.L. fem. pl. n. *Thermacetogeniales* the *Thermacetogenium* order	The order constitutes a monophyletic lineage as described and defined in Parks et al. (2018). The order contains the family *Thermacetogeniaceae*^3^
*Thermaerobacterales*	Genus *Thermaerobacter* Takai et al. 1999	Therm.a.e.ro.bac.te.ra'les. N.L. masc. n. *Thermaerobacter* type genus of the order; -*ales* ending to denote an order; N.L. fem. pl. n. *Thermaerobacterales* the *Thermaerobacter* order	The order constitutes a monophyletic lineage as described and defined in Parks et al. (2018). The order contains the family *Thermaerobacteraceae*^3^
*Thermicanales*	Genus *Thermicanus* Gößner et al. 2000	Ther.mi.ca.na'les. N.L. masc. n. *Thermicanus* type genus of the order; -*ales* ending to denote an order; N.L. fem. pl. n. *Thermicanales* the *Thermicanus* order	The order constitutes a monophyletic lineage as described and defined in Parks et al. (2018). The order contains the family *Thermicanaceae*^3^
*Thermincolales*	Genus *Thermincola* Sokolova et al. 2005	Therm.in.co.la'les. N.L. fem. n. *Thermincola* type genus of the order; -*ales* ending to denote an order; N.L. fem. pl. n. *Thermincolales* the *Thermincola* order	The order constitutes a monophyletic lineage as described and defined in Parks et al. (2018). The order contains the family *Thermincolaceae*
*Thermoactinomycetales*	Genus *Thermoactinomyces* Tsiklinsky 1899 (Approved Lists 1980)	Ther.mo.ac.ti.no.my.ce.ta'les. N.L. masc. n. *Thermoactinomyces* type genus of the order; -*ales* ending to denote an order; N.L. fem. pl. n *Thermoactinomycetales* the *Thermoactinomyces* order	The order constitutes a monophyletic lineage as described and defined in Parks et al. (2018). The order contains the following families: *Thermoactinomycetaceae, Novibacillaceae*
*Thermosulfidibacterales*	Genus *Thermosulfidibacter* Nunoura et al. 2008	Ther.mo.sulfi.di.bac.te.ra'les. N.L. masc. n. *Thermosulfidibacter* type genus of the order; -*ales* ending to denote an order; N.L. fem. pl. n. *Thermosulfidibacterales* the *Thermosulfidibacter* order	The order constitutes a monophyletic lineage as described and defined in Parks et al. (2018). The order contains the family *Thermosulfidibacteraceae*^3^
*Thiomicrospirales*	Genus *Thiomicrospira* Kuenen and Veldkamp 1972 (Approved Lists 1980)	Thi.o.mic.ro.spi.ra'les. N.L. fem. n. *Thiomicrospira* type genus of the order; -*ales* ending to denote an order; N.L. fem. pl. n. *Thiomicrospirales* the *Thiomicrospira* order	The order constitutes a monophyletic lineage as described and defined in Parks et al. (2018). The order contains the family ‘*Thiomicrospiraceae*’
*Tistrellales*	Genus *Tistrella* Shi et al. 2003	Tis.trel.la'les. N.L. fem. n. *Tistrella* type genus of the order; -*ales* ending to denote an order; N.L. fem. pl. n. *Tistrellales* the *Tistrella* order	The order constitutes a monophyletic lineage as described and defined in Parks et al. (2018). The order contains the family *Tistrellaceae*^3^
*Treponematales*	Genus *Treponema* Schaudinn 1905 (Approved Lists 1980)	Tre.po.ne.ma.ta'les. N.L. neut. n. *Treponema* type genus of the order; -*ales* ending to denote an order; N.L. fem. pl. n. *Treponematales* the *Treponema* order	The order constitutes a monophyletic lineage as described and defined in Parks et al. (2018). The order contains the family *Treponemataceae*
*Tumebacillales*	Genus *Tumebacillus* Steven et al. 2008	Tu.me.ba.cil.la'les. N.L. masc. n. *Tumebacillus* type genus of the order; -*ales* ending to denote an order; N.L. fem. pl. n. *Tumebacillales* the *Tumebacillus* order	The order constitutes a monophyletic lineage as described and defined in Parks et al. (2018). The order contains the following families: *Tumebacillaceae*,^3^*Effusibacillaceae*^3^
*Turneriellales*	Genus *Turneriella* Levett et al. 2005	Tur.ne.ri.el.la'les. N.L. fem. n. *Turneriella* type genus of the order; -*ales* ending to denote an order; N.L. fem. pl. n. *Turneriellales* the *Turneriella* order	The order constitutes a monophyletic lineage as described and defined in Parks et al. (2018). The order contains the family *Turneriellaceae*^3^
*Vampirovibrionales* (type and taxon description are missing from Soo et al. 2015 where a taxon was originally proposed)	Genus *Vampirovibrio* Gromov and Mamkayeva 1980	Vam.pi.ro.vi.bri.o.na'les. N.L. masc. n. *Vampirovibrio* type genus of the order; -*ales* ending to denote an order; N.L. fem. pl. n. *Vampirovibrionales* the *Vampirovibrio* order	The order constitutes a monophyletic lineage as described and defined in Parks et al. (2018). The order contains the family *Vampirovibrionaceae*^3^
*Woeseiales*	Genus *Woeseia* Du et al. 2016	*Woe.se.i.a'les*. N.L. fem. n. *Woeseia* type genus of the order; -*ales* ending to denote an order; N.L. fem. pl. n. *Woeseiales* the *Woeseia* order	The order constitutes a monophyletic lineage as described and defined in Parks et al. (2018). The order contains the family *Woeseiaceae*
*Zavarziniales*	Genus *Zavarzinia* Meyer et al. 1994	Za.var.zi.ni.a'les. N.L. fem. n. *Zavarzinia* type genus of the order; -*ales* ending to denote an order; N.L. fem. pl. n. *Zavarziniales* the *Zavarzinia* order	The order constitutes a monophyletic lineage as described and defined in Parks et al. (2018). The order contains the family *Zavarziniaceae*
**Rank of Family (all proposed as fam. nov.)**			
*Acetivibrionaceae* (replacement name for illegitimate *Hungateiclostridiaceae* Zhang et al. 2018)	Genus *Acetivibrio* Patel et al. 1980	A.ce.ti.vib.ri.o.na.ce'ae. N.L. masc. n. *Acetivibrio* type genus of the family; -*aceae* ending to denote a family; N.L. fem. pl. n. *Acetivibrionaceae* the *Acetivibrio* family	The family constitutes a monophyletic lineage as described and defined in Parks et al. (2018). The family contains the following genera: *Acetivibrio, Anaerobacterium, Herbivorax, Hungateiclostridium, Pseudobacteroides, ‘*Pseudoclostridium*’, Ruminiclostridium*
*Acetohalobiaceae*	Genus *Acetohalobium* Zhilina and Zavarzin 1990	A.ce.to.ha.lo.bi.a.ce'ae. N.L. neut. n. *Acetohalobium* type genus of the family; -*aceae* ending to denote a family; N.L. fem. pl. n. *Acetohalobiaceae* the *Acetohalobium* family	The family constitutes a monophyletic lineage as described and defined in Parks et al. (2018). The family contains the following genera: *Acetohalobium, Candidatus* Frackibacter, ‘*Selenihalanaerobacter*’
*Acetonemataceae* (corrig. of ‘*Acetonemaceae*’)	Genus *Acetonema* Kane and Breznak 1992	A.ce.to.ne.ma.ta.ce'ae. N.L. neut. n. *Acetonema* type genus of the family; -*aceae* ending to denote a family; N.L. fem. pl. n. *Acetonemataceae* the *Acetonema* family	The family constitutes a monophyletic lineage as described and defined in Parks et al. (2018). The family contains the following genera: *Acetonema, Anaerosporomusa*
*Acidaminobacteraceae*	Genus *Acidaminobacter* Stams and Hansen 1985	A.cid.a.mi.no.bac.te.ra.ce'ae. N.L. masc. n. *Acidaminobacter* type genus of the family; -*aceae* ending to denote a family; N.L. fem. pl. n. *Acidaminobacteraceae* the *Acidaminobacter* family	The family constitutes a monophyletic lineage as described and defined in Parks et al. (2018). The family contains the genus *Acidaminobacter*
*Acidihalobacteraceae*	Genus *Acidihalobacter* Cárdenas et al. 2015	A.ci.di.ha.lo.bac.te.ra.ce'ae. N.L. masc. n. *Acidihalobacter* type genus of the family; -*aceae* ending to denote a family; N.L. fem. pl. n. *Acidihalobacteraceae* the *Acidihalobacter* family	The family constitutes a monophyletic lineage as described and defined in Parks et al. (2018). The family contains the genus *Acidihalobacter*
*Acutalibacteraceae*	Genus *Acutalibacter* Lagkouvardos et al. 2016	A.cu.ta.li.bac.te.ra.ce'ae. N.L. masc. n. *Acutalibacter* type genus of the family; -*aceae* ending to denote a family; N.L. fem. pl. n. *Acutalibacteraceae* the *Acutalibacter* family	The family constitutes a monophyletic lineage as described and defined in Parks et al. (2018). The family contains the following genera: *Acutalibacter*, ‘*Anaeromassilibacillus*’, *Caproicibacter, Caproiciproducens, Hydrogeniiclostidium*, ‘*Pseudoruminococcus*’
*Aeribacillaceae*	Genus *Aeribacillus* Miñana-Galbis et al. 2010	A.e.ri.ba.cil.la.ce'ae. N.L. masc. n. *Aeribacillus* type genus of the family; -*aceae* ending to denote a family; N.L. fem. pl. n. *Aeribacillaceae* the *Aeribacillus* family	The family constitutes a monophyletic lineage as described and defined in Parks et al. (2018). The family contains the genus *Aeribacillus*
*Ahniellaceae*	Genus *Ahniella* Hwang et al. 2018	Ahn.i.el.la.ce'ae. N.L. fem. n. *Ahniella* type genus of the family; -*aceae* ending to denote a family; N.L. fem. pl. n. *Ahniellaceae* the *Ahniella* family	The family constitutes a monophyletic lineage as described and defined in Parks et al. (2018). The family contains the genus *Ahniella*
*Alkalibacillaceae*	Genus *Alkalibacillus* Jeon et al. 2005	Al.ka.li.ba.cil.la.ce'ae. N.L. masc. n. *Alkalibacillus* type genus of the family; -*aceae* ending to denote a family; N.L. fem. pl. n. *Alkalibacillaceae* the *Alkalibacillus* family	The family constitutes a monophyletic lineage as described and defined in Parks et al. (2018). The family contains the following genera: *Alkalibacillus, Aquisalibacillus, Filobacillus, Halalkalibacillus, Melghiribacillus, Piscibacillus, Salinibacillus, Salirhabdus, Tenuibacillus*
*Alkalibacteraceae*	Genus *Alkalibacter* Garnova et al. 2005	Al.ka.li.bac.te.ra.ce'ae. N.L. masc. n. *Alkalibacter* type genus of the family; -*aceae* ending to denote a family; N.L. fem. pl. n. *Alkalibacteraceae* the *Alkalibacter* family	The family constitutes a monophyletic lineage as described and defined in Parks et al. (2018). The family contains the following genera: *Alkalibacter, Alkalibaculum*
*Alkalispirochaetaceae*	Genus *Alkalispirochaeta* Sravanthi et al. 2016	Al.ka.li.spi.ro.chae.ta.ce'ae. N.L. fem. n. *Alkalispirochaeta* type genus of the family; -*aceae* ending to denote a family; N.L. fem. pl. n. *Alkalispirochaetaceae* the *Alkalispirochaeta* family	The family constitutes a monophyletic lineage as described and defined in Parks et al. (2018). The family contains the genus *Alkalispirochaeta*
*Ammonificaceae*	Genus *Ammonifex* Huber and Stetter 1996	Am.mo.ni.fi.ca.ce'ae. N.L. masc. n. *Ammonifex* type genus of the family; -*aceae* ending to denote a family; N.L. fem. pl. n. *Ammonificaceae* the *Ammonifex* family	The family constitutes a monophyletic lineage as described and defined in Parks et al. (2018). The family contains the following genera: *Ammonifex, Thermodesulfitimonas*
*Amphibacillaceae*	Genus *Amphibacillus* Niimura et al. 1990	Am.phi.ba.cil.la.ce'ae. N.L. masc. n. *Amphibacillus* type genus of the family; -*aceae* ending to denote a family; N.L. fem. pl. n. *Amphibacillaceae* the *Amphibacillus* family	The family constitutes a monophyletic lineage as described and defined in Parks et al. (2018). The family contains the following genera: *Amphibacillus, Aquibacillus, Cerasibacillus, Gracilibacillus, Halolactibacillus, Lentibacillus, Oceanobacillus, Ornithinibacillus, Paraliobacillus, Pelagirhabdus, Pseudogracilibacillus, Saliterribacillus, Sediminibacillus, Streptohalobacillus, Terribacillus, Virgibacillus*
*Anaerobacillaceae*	Genus *Anaerobacillus* Zavarzina et al. 2010	An.a.e.ro.ba.cil.la.ce'ae. N.L. masc. n. *Anaerobacillus* type genus of the family; -*aceae* ending to denote a family; N.L. fem. pl. n. *Anaerobacillaceae* the *Anaerobacillus* family	The family constitutes a monophyletic lineage as described and defined in Parks et al. (2018). The family contains the genus *Anaerobacillus*
*Anaerofustaceae*	Genus *Anaerofustis* Finegold et al. 2004	An.a.e.ro.fus.ta.ce'ae. N.L. masc. n. *Anaerofustis* type genus of the family; -*aceae* ending to denote a family; N.L. fem. pl. n. *Anaerofustaceae* the *Anaerofustis* family	The family constitutes a monophyletic lineage as described and defined in Parks et al. (2018). The family contains the genus *Anaerofustis*
*Anaeromusaceae*	Genus *Anaeromusa* Baena et al. 1999	An.a.e.ro.mu.sa.ce'ae. N.L. fem. n. *Anaeromusa* type genus of the order; -*aceae* ending to denote a family; N.L. fem. pl. n. *Anaeromusaceae* the *Anaeromusa* family	The family constitutes a monophyletic lineage as described and defined in Parks et al. (2018). The family contains the genus *Anaeromusa*
*Anaerotignaceae*	Genus *Anaerotignum* Ueki et al. 2017	An.a.e.ro.tig.na.ce'ae. N.L. neut. n. *Anaerotignum* type genus of the family; -*aceae* ending to denote a family; N.L. fem. pl. n. *Anaerotignaceae* the *Anaerotignum* family	The family constitutes a monophyletic lineage as described and defined in Parks et al. (2018). The family contains the genus *Anaerotignum*
*Anaerovoracaceae*	Genus *Anaerovorax* Matthies et al. 2000	An.a.e.ro.vo.ra.ca.ce'ae. N.L. masc. n. *Anaerovorax* type genus of the family; -*aceae* ending to denote a family; N.L. fem. pl. n. *Anaerovoracaceae* the *Anaerovorax* family	The family constitutes a monophyletic lineage as described and defined in Parks et al. (2018). The family contains the following genera: *Anaerovorax, Aminicella, Aminipila, Mogibacterium, ‘Alterileibacterium’, ‘Bacilliculturomica’,‘Emergencia’, ‘Mobilibacterium’*
*Aneurinibacillaceae*	Genus *Aneurinibacillus* Shida et al. 1996	A.neu.ri.ni.ba.cil.lac'ea. N.L. masc. n. *Aneurinibacillus* type genus of the family; -*aceae* ending to denote a family; N.L. fem. pl. n. *Aneurinibacillacea* the *Aneurinibacillus* family	The family constitutes a monophyletic lineage as described and defined in Parks et al. (2018). The family contains the genus *Aneurinibacillus*
*Anoxybacillaceae*	Genus *Anoxybacillus* Pikuta et al. 2000	An.o.xy.ba.cil.la.ce'ae. N.L. masc. n. *Anoxybacillus* type genus of the family; -*aceae* ending to denote a family; N.L. fem. pl. n. *Anoxybacillaceae* the *Anoxybacillus* family	The family constitutes a monophyletic lineage as described and defined in Parks et al. (2018). The family contains the following genera: *Anoxybacillus, Geobacillus, Parageobacillus, Saccharococcus, Thermolongibacillus*
*Aquisalimonadaceae*	Genus *Aquisalimonas* Márquez et al. 2007	A.qui.sa.li.mo.na.da.ce'ae. N.L. fem. n. *Aquisalimonas* type genus of the family; -*aceae* ending to denote a family; N.L. fem. pl. n. *Aquisalimonadaceae* the *Aquisalimonas* family	The family constitutes a monophyletic lineage as described and defined in Parks et al. (2018). The family contains the genus *Aquisalimonas*
*Brevibacillaceae*	Genus *Brevibacillus* Shida et al. 1996	Bre.vi.ba.cil.la.ce'ae. N.L. masc. n. *Brevibacillus* type genus of the family; -*aceae* ending to denote a family; N.L. fem. pl. n. *Brevibacillaceae* the *Brevibacillus* family	The family constitutes a monophyletic lineage as described and defined in Parks et al. (2018). The family contains the genus *Brevibacillus*
*Butyricicoccaceae*	Genus *Butyricicoccus* Eeckhaut et al. 2008	Bu.ty.ri.ci.coc.ca.ce'ae. N.L. masc. n. *Butyricicoccus* type genus of the family; -*aceae* ending to denote a family; N.L. fem. pl. n. *Butyricicoccaceae* the *Butyricicoccus* family	The family constitutes a monophyletic lineage as described and defined in Parks et al. (2018). The family contains the following genera: *Butyricicoccus, Agathobaculum*, ‘*Intestinibacillus*’
*Caldalkalibacillaceae*	Genus *Caldalkalibacillus* Xue et al. 2006	Cald.al.ka.li.ba.cil.la.ce'ae. N.L. masc. n. *Caldalkalibacillus* type genus of the family; -*aceae* ending to denote a family; N.L. fem. pl. n. *Caldalkalibacillaceae* the *Caldalkalibacillus* family	The family constitutes a monophyletic lineage as described and defined in Parks et al. (2018). The family contains the genus *Caldalkalibacillus*
*Caldanaerobiaceae*	Genus *Caldanaerobius* Lee et al. 2008	Cald.an.a.e.ro.bi.a.ce'ae. N.L. masc. n. *Caldanaerobius* type genus of the family; -*aceae* ending to denote a family; N.L. fem. pl. n. *Caldanaerobiaceae* the *Caldanaerobius* family	The family constitutes a monophyletic lineage as described and defined in Parks et al. (2018). The family contains the genus *Caldanaerobius*
*Caldibacillaceae*	Genus *Caldibacillus* Coorevits et al. 2012	Cal.di.ba.cil.la.ce'ae. N.L. masc. n. *Caldibacillus* type genus of the family; -*aceae* ending to denote a family; N.L. fem. pl. n. *Caldibacillaceae* the *Caldibacillus* family	The family constitutes a monophyletic lineage as described and defined in Parks et al. (2018). The family contains the genus *Caldibacillus*
*Caldicellulosiruptoraceae*	Genus *Caldicellulosiruptor* Rainey et al. 1995	Cal.di.cel.lu.lo.si.rup.to.ra.ce'ae. N.L. masc. n. *Caldicellulosiruptor* type genus of the family; -*aceae* ending to denote a family; N.L. fem. pl. n. *Caldicellulosiruptoraceae* the *Caldicellulosiruptor* family	The family constitutes a monophyletic lineage as described and defined in Parks et al. (2018). The family contains the genus *Caldicellulosiruptor*
*Caldisalinibacteraceae*	Genus *Caldisalinibacter* Ben Hania et al. 2015	Cal.di.sa.li.ni.bac.te.ra.ce'ae. N.L. masc. n. *Caldisalinibacter* type genus of the family; -*aceae* ending to denote a family; N.L. fem. pl. n. *Caldisalinibacteraceae* the *Caldisalinibacter* family	The family constitutes a monophyletic lineage as described and defined in Parks et al. (2018). The family contains the genus *Caldisalinibacter*
*Calditerricolaceae*	Genus *Calditerricola* Moriya et al. 2011	Cal.di.ter.ri.co.la.ce'ae. N.L. masc. n. *Calditerricola* type genus of the family; -*aceae* ending to denote a family; N.L. fem. pl. n. *Calditerricolaceae* the *Calditerricola* family	The family constitutes a monophyletic lineage as described and defined in Parks et al. (2018). The family contains the genus *Calditerricola*
*Caloramatoraceae*	Genus *Caloramator* Collins et al. 1994	Ca.lo.ra.ma.to.ra.ce'ae. N.L. masc. n. *Caloramator* type genus of the family; -*aceae* ending to denote a family; N.L. fem. pl. n. *Caloramatoraceae* the *Caloramator* family	The family constitutes a monophyletic lineage as described and defined in Parks et al. (2018). The family contains the following genera: *Caloramator, Fervidicella, Fonticella, Thermobrachium*
*Caminicellaceae*	Genus *Caminicella* Alain et al. 2002	Ca.mi.ni.cel.la.ce'ae. N.L. fem. n. *Caminicella* type genus of the family; -*aceae* ending to denote a family; N.L. fem. pl. n. *Caminicellaceae* the *Caminicella* family	The family constitutes a monophyletic lineage as described and defined in Parks et al. (2018). The family contains the following genera: *Caminicella, Maledivibacter, Paramaledivibacter, Candidatus* Petromonas
*Carboxydocellaceae*	Genus *Carboxydocella* Sokolova et al. 2002	Car.bo.xy.do.cel.la.ce'ae. N.L. fem. n. *Carboxydocella* type genus of the family; -*aceae* ending to denote a family; N.L. fem. pl. n. *Carboxydocellaceae* the *Carboxydocella* family	The family constitutes a monophyletic lineage as described and defined in Parks et al. (2018). The family contains the genus *Carboxydocella*
*Carboxydothermaceae*	Genus *Carboxydothermus* Svetlichny et al. 1991	Car.bo.xy.do.ther.ma.ce'ae. N.L. masc. n. *Carboxydothermus* type genus of the family; -*aceae* ending to denote a family; N.L. fem. pl. n. *Carboxydothermaceae* the *Carboxydothermus* family	The family constitutes a monophyletic lineage as described and defined in Parks et al. (2018). The family contains the genus *Carboxydothermus*
*Catellicoccaceae*	Genus *Catellicoccus* Lawson et al. 2006	Ca.tel.li.coc.ca.ce'ae. N.L. masc. n. *Catellicoccus* type genus of the family; -*aceae* ending to denote a family; N.L. fem. pl. n. *Catellicoccaceae* the *Catellicoccus* family	The family constitutes a monophyletic lineage as described and defined in Parks et al. (2018). The family contains the genus *Catellicoccus*
*Chitinimonadaceae*	Genus *Chitinimonas* Chang et al. 2004	Chi.ti.ni.mo.na.da.ce'ae. N.L. fem. n. *Chitinimonas* type genus of the family; -*aceae* ending to denote a family; N.L. fem. pl. n. *Chitinimonadaceae* the *Chitinimonas* family	The family constitutes a monophyletic lineage as described and defined in Parks et al. (2018). The family contains the genus *Chitinimonas*
*Clostridiisalibacteraceae*	Genus *Clostridiisalibacter* Liebgott et al. 2008	Clos.tri.di.i.sa.li.bac.te.ra.ce'ae. N.L. masc. n. *Clostridiisalibacter* type genus of the family; -*aceae* ending to denote a family; N.L. fem. pl. n. *Clostridiisalibacteraceae* the *Clostridiisalibacter* family	The family constitutes a monophyletic lineage as described and defined in Parks et al. (2018). The family contains the genus *Clostridiisalibacter*
*Coprobacteraceae*	Genus *Coprobacter* Shkoporov et al. 2013	Cop.ro.bac.te.ra.ce'ae. N.L. masc. n. *Coprobacter* type genus of the family; -*aceae* ending to denote a family; N.L. fem. pl. n. *Coprobacteraceae* the *Coprobacter* family	The family constitutes a monophyletic lineage as described and defined in Parks et al. (2018). The family contains the genus *Coprobacter*
*Cycloclasticaceae*	Genus *Cycloclasticus* Cyksterhouse et al. 1995	Cyc.lo.cla.sti.ca.ce'ae. N.L. masc. n. *Cycloclasticus* type genus of the family; -*aceae* ending to denote a family; N.L. fem. pl. n. *Cycloclasticaceae* the *Cycloclasticus* family	The family constitutes a monophyletic lineage as described and defined in Parks et al. (2018). The family contains the genus *Cycloclasticus*
*Dendrosporobacteraceae*	Genus *Dendrosporobacter* Strömpl et al. 2000	Den.dro.spo.ro.bac.te.ra.ce'ae. N.L. masc. n. *Dendrosporobacter* type genus of the family; -*aceae* ending to denote a family; N.L. fem. pl. n. *Dendrosporobacteraceae* the *Dendrosporobacter* family	The family constitutes a monophyletic lineage as described and defined in Parks et al. (2018). The family contains the genus *Dendrosporobacter*
*Desulfonisporaceae*	Genus *Desulfonispora* Denger et al. 1999	De.sul.fo.ni.spo.ra.ce'ae. N.L. fem. n. *Desulfonispora* type genus of the family; -*aceae* ending to denote a family; N.L. fem. pl. n. *Desulfonisporaceae* the *Desulfonispora* family	The family constitutes a monophyletic lineage as described and defined in Parks et al. (2018). The family contains the genus *Desulfonispora*
*Desulfovirgulaceae*	Genus *Desulfovirgula* Kaksonen et al. 2007	De.sul.fo.vir.gu.la.ce'ae. N.L. fem. n. *Desulfovirgula* type genus of the family; -*aceae* ending to denote a family; N.L. fem. pl. n. *Desulfovirgulaceae* the *Desulfovirgula* family	The family constitutes a monophyletic lineage as described and defined in Parks et al. (2018). The family contains the following genera: *Desulfovirgula, Desulfofundulus*
*Dethiosulfatibacteraceae*	Genus *Dethiosulfatibacter* Takii et al. 2007	De.thi.o.sul.fa.ti.bac.te.ra.ce'ae. N.L. masc. n. *Dethiosulfatibacter* type genus of the family; -*aceae* ending to denote a family; N.L. fem. pl. n. *Dethiosulfatibacteraceae* the *Dethiosulfatibacter* family	The family constitutes a monophyletic lineage as described and defined in Parks et al. (2018). The family contains the genus *Dethiosulfatibacter*
*Dialisteraceae*	Genus *Dialister* (ex Bergey et al. 1923) Moore and Moore 1994	Di.a.li.ste.ra.ce'ae. N.L. masc. n. *Dialister* type genus of the family; -*aceae* ending to denote a family; N.L. fem. pl. n. *Dialisteraceae* the *Dialister* family	The family constitutes a monophyletic lineage as described and defined in Parks et al. (2018). The family contains the following genera: *Dialister, Allisonella*
*Diplorickettsiaceae*	Genus *Diplorickettsia* Mediannikov et al. 2011	Di.plo.ric.kett.si.a.ce'ae. N.L. fem. n. *Diplorickettsia* type genus of the family; -*aceae* ending to denote a family; N.L. fem. pl. n. *Diplorickettsiaceae* the *Diplorickettsia* family	The family constitutes a monophyletic lineage as described and defined in Parks et al. (2018). The family contains the genus *Diplorickettsia*
*Effusibacillaceae*	Genus *Effusibacillus* Watanabe et al. 2014	Ef.fu.si.ba.cil.la.ce'ae. N.L. masc. n. *Effusibacillus* type genus of the family; -*aceae* ending to denote a family; N.L. fem. pl. n. *Effusibacillaceae* the *Effusibacillus* family	The family constitutes a monophyletic lineage as described and defined in Parks et al. (2018). The family contains the genus *Effusibacillus*
*Elainellaceae*	Genus *Elainella* Jahodářová et al. 2018	E.lai.nel.la.ce'ae. N.L. fem. n. *Elainella* type genus of the family; -*aceae* ending to denote a family; N.L. fem. pl. n. *Elainellaceae* the *Elainella* family	The family constitutes a monophyletic lineage as described and defined in Parks et al. (2018). The family contains the genus *Elainella*
*Elsteraceae*	Genus *Elstera* Rahalkar et al. 2012	El.ste.ra.ce'ae. N.L. fem. n. *Elstera* type genus of the family; -*aceae* ending to denote a family; N.L. fem. pl. n. *Elsteraceae* the *Elstera* family	The family constitutes a monophyletic lineage as described and defined in Parks et al. (2018). The family contains the genus *Elstera*
*Ethanoligenentaceae* (corrig. of *Ethanoligenenaceae*)	Genus *Ethanoligenens* Xing et al. 2006	E.tha.no.li.ge.ne.n.ta.ce'ae. N.L. neut. n. *Ethanoligenens* type genus of the family; -*aceae* ending to denote a family; N.L. fem. pl. n. *Ethanoligenentaceae* the *Ethanoligenens* family	The family constitutes a monophyletic lineage as described and defined in Parks et al. (2018). The family contains the genus *Ethanoligenens*
*Exiguobacteriaceae* (corrig. of ‘*Exiguobacteraceae’*)	Genus *Exiguobacterium* Collins et al. 1984	Ex.i.gu.o.bac.te.ri.a.ce'ae. N.L. neut. n. *Exiguobacterium* type genus of the family; -*aceae* ending to denote a family; N.L. fem. pl. n. *Exiguobacteriaceae* the *Exiguobacterium* family	The family constitutes a monophyletic lineage as described and defined in Parks et al. (2018). The family contains the genus *Exiguobacterium*
*Fastidiosipilaceae*	Genus *Fastidiosipila* Falsen et al. 2005	Fas.ti.di.o.si.pi.la.ce'ae. N.L. fem. n. *Fastidiosipila* type genus of the family; -*aceae* ending to denote a family; N.L. fem. pl. n. *Fastidiosipilaceae* the *Fastidiosipila* family	The family constitutes a monophyletic lineage as described and defined in Parks et al. (2018). The family contains the following genera: *Fastidiosipila, Mageeibacillus*
*Ferrovibrionaceae*	Genus *Ferrovibrio* Sorokina et al. 2013	Fer.ro.vib.ri.o.na.ce'ae. N.L. masc. n. *Ferrovibrio* type genus of the family; -*aceae* ending to denote a family; N.L. fem. pl. n. *Ferrovibrionaceae* the *Ferrovibrio* family	The family constitutes a monophyletic lineage as described and defined in Parks et al. (2018). The family contains the genus *Ferrovibrio*
*Fictibacillaceae*	Genus *Fictibacillus* Glaeser et al. 2013	Fic.ti.ba.cil.la.ce'ae. N.L. masc. n. *Fictibacillus* type genus of the family; -*aceae* ending to denote a family; N.L. fem. pl. n. *Fictibacillaceae* the *Fictibacillus* family	The family constitutes a monophyletic lineage as described and defined in Parks et al. (2018). The family contains the genus *Fictibacillus*
*Filifactoraceae*	Genus *Filifactor* Collins et al. 1994	Fi.li.fac.to.ra.ce'ae. N.L. masc. n. *Filifactor* type genus of the family; -*aceae* ending to denote a family; N.L. fem. pl. n. *Filifactoraceae* the *Filifactor* family	The family constitutes a monophyletic lineage as described and defined in Parks et al. (2018). The family contains the following genera: *Filifactor, Acetoanaerobium, Criibacterium, Peptoanaerobacter, Proteocatella*
*Gallaecimonadaceae*	Genus *Gallaecimonas* Rodríguez-Blanco et al. 2010	Gal.lae.ci.mo.na.da.ce'ae. N.L. fem. n. *Gallaecimonas* type genus of the family; -*aceae* ending to denote a family; N.L. fem. pl. n. *Gallaecimonadaceae* the *Gallaecimonas* family	The family constitutes a monophyletic lineage as described and defined in Parks et al. (2018). The family contains the genus *Gallaecimonas*
*Garciellaceae*	Genus *Garciella* Miranda-Tello et al. 2003	Gar.ci.el.la.ce'ae. N.L. fem. n. *Garciella* type genus of the family; -*aceae* ending to denote a family; N.L. fem. pl. n. *Garciellaceae* the *Garciella* family	The family constitutes a monophyletic lineage as described and defined in Parks et al. (2018). The family contains the genus *Garciella*
*Gemellaceae*	Genus *Gemella* Berger 1960 (Approved Lists 1980)	Ge.mel.la.ce'ae. N.L. fem. n. *Gemella* type genus of the family; -*aceae* ending to denote a family; N.L. fem. pl. n. *Gemellaceae* the *Gemella* family	The family constitutes a monophyletic lineage as described and defined in Parks et al. (2018). The family contains the genus *Gemella*
*Haladaptataceae*	Genus *Haladaptatus* Savage et al. 2007 emend. (Cui et al. 2010)	Hal.a.dap.ta.ta.ce'ae. N.L. masc. n. *Haladaptatus* type genus of the family; -*aceae* ending to denote a family; N.L. fem. pl. n. *Haladaptataceae* the *Haladaptatus* family	The family constitutes a monophyletic lineage as described and defined in Parks et al. (2018). The family contains the genus *Haladaptatus*
*Halobacillaceae*	Genus *Halobacillus* Spring et al. 1996	Ha.lo.ba.cil.la.ce'ae. N.L. masc. n. *Halobacillus* type genus of the family; -*aceae* ending to denote a family; N.L. fem. pl. n. *Halobacillaceae* the *Halobacillus* family	The family constitutes a monophyletic lineage as described and defined in Parks et al. (2018). The family contains the following genera: *Halobacillus, Pontibacillus, Salimicrobium, Thalassobacillus*
*Halofilaceae*	Genus *Halofilum* Xia et al. 2017	Ha.lo.fi.la.ce'ae. N.L. neut. n. *Halofilum* type genus of the family; -*aceae* ending to denote a family; N.L. fem. pl. n. *Halofilaceae* the *Halofilum* family	The family constitutes a monophyletic lineage as described and defined in Parks et al. (2018). The family contains the genus *Halofilum*
*Ignicoccaceae*	Genus *Ignicoccus* Huber et al. 2000	Ig.ni.coc.ca.ce'ae. N.L. masc. n. *Ignicoccus* type genus of the family; -*aceae* ending to denote a family; N.L. fem. pl. n. *Ignicoccaceae* the *Ignicoccus* family	The family constitutes a monophyletic lineage as described and defined in Parks et al. (2018). The family contains the genus *Ignicoccus*
*Ignisphaeraceae*	Genus *Ignisphaera* Niederberger et al. 2006	Ig.ni.sphae.ra.ce'ae. N.L. fem. n. *Ignisphaera* type genus of the family; -*aceae* ending to denote a family; N.L. fem. pl. n. *Ignisphaeraceae* the *Ignisphaera* family	The family constitutes a monophyletic lineage as described and defined in Parks et al. (2018). The family contains the genus *Ignisphaera*
*Inquilinaceae*	Genus *Inquilinus* Coenye et al. 2002	In.qui.li.na.ce'ae. N.L. masc. n. *Inquilinus* type genus of the family; -*aceae* ending to denote a family; N.L. fem. pl. n. *Inquilinaceae* the *Inquilinus* family	The family constitutes a monophyletic lineage as described and defined in Parks et al. (2018). The family contains the genus *Inquilinus*
*Jeotgalibacillaceae*	Genus *Jeotgalibacillus* Yoon et al. 2001	Jeot.ga.li.ba.cil.la.ce'ae. N.L. masc. n. *Jeotgalibacillus* type genus of the family; -*aceae* ending to denote a family; N.L. fem. pl. n. *Jeotgalibacillaceae* the *Jeotgalibacillus* family	The family constitutes a monophyletic lineage as described and defined in Parks et al. (2018). The family contains the genus *Jeotgalibacillus*
*Ketobacteraceae*	Genus *Ketobacter* Kim et al. 2018	Ke.to.bac.te.ra.ce'ae. N.L. masc. n. *Ketobacter* type genus of the family; -*aceae* ending to denote a family; N.L. fem. pl. n. *Ketobacteraceae* the *Ketobacter* family	The family constitutes a monophyletic lineage as described and defined in Parks et al. (2018). The family contains the genus *Ketobacter*
*Kyrpidiaceae*	Genus *Kyrpidia* Klenk et al. 2012	Kyr.pi.di.a.ce'ae. N.L. fem. n. *Kyrpidia* type genus of the family; -*aceae* ending to denote a family; N.L. fem. pl. n. *Kyrpidiaceae* the *Kyrpidia* family	The family constitutes a monophyletic lineage as described and defined in Parks et al. (2018). The family contains the genus *Kyrpidia*
*Leptonemataceae*	Genus *Leptonema* Hovind-Hougen 1983	Lep.to.ne.ma.ta.ce'ae. N.L. neut. n. *Leptonema* type genus of the family; -*aceae* ending to denote a family; N.L. fem. pl. n. *Leptonemataceae* the *Leptonema* family	The family constitutes a monophyletic lineage as described and defined in Parks et al. (2018). The family contains the genus *Leptonema*
*Leptospirillaceae*	Genus *Leptospirillum* (*ex* Markosyan 1972) Hippe 2000	Lep.to.spi.ril.la.ce'ae. N.L. neut. n. *Leptospirillum* type genus of the family; -*aceae* ending to denote a family; N.L. fem. pl. n. *Leptospirillaceae* the *Leptospirillum* family	The family constitutes a monophyletic lineage as described and defined in Parks et al. (2018). The family contains the genus *Leptospirillum*
*Lutisporaceae*	Genus *Lutispora* Shiratori et al. 2008	Lu.ti.spo.ra.ce'ae. N.L. fem. n. *Lutispora* type genus of the family; -*aceae* ending to denote a family; N.L. fem. pl. n. *Lutispora*ceae the *Lutispora* family	The family constitutes a monophyletic lineage as described and defined in Parks et al. (2018). The family contains the genus *Lutispora*
*Mahellaceae*	Genus *Mahella* Bonilla Salinas et al. 2004	Ma.hel.la.ce'ae. N.L. fem. n. *Mahella* type genus of the family; -*aceae* ending to denote a family; N.L. fem. pl. n. *Mahellaceae* the *Mahella* family	The family constitutes a monophyletic lineage as described and defined in Parks et al. (2018). The family contains the genus *Mahella*
*Marinicellaceae*	Genus *Marinicella* Romanenko et al. 2010	Ma.ri.ni.cel.la.ce'ae. N.L. fem. n. *Marinicella* type genus of the family; -*aceae* ending to denote a family; N.L. fem. pl. n. *Marinicellaceae* the *Marinicella* family	The family constitutes a monophyletic lineage as described and defined in Parks et al. (2018). The family contains the genus *Marinicella*
*Marinithermaceae*	Genus *Marinithermus* Sako et al. 2003	Ma.ri.ni.ther.ma.ce'ae. N.L. masc. n. *Marinithermus* type genus of the family; -*aceae* ending to denote a family; N.L. fem. pl. n. *Marinithermaceae* the *Marinithermus* family	The family constitutes a monophyletic lineage as described and defined in Parks et al. (2018). The family contains the following genera: *Marinithermus, Oceanithermus*
*Marinococcaceae*	Genus *Marinococcus* Hao et al. 1985	Ma.ri.no.coc.ca.ce'ae. N.L. masc. n. *Marinococcus* type genus of the family; -*aceae* ending to denote a family; N.L. fem. pl. n. *Marinococcaceae* the *Marinococcus* family	The family constitutes a monophyletic lineage as described and defined in Parks et al. (2018). The family contains the following genera: *Marinococcus, Alteribacillus, Geomicrobium, Natribacillus, Salibacterium, Salicibibacter, Salsuginibacillus, Sinobaca*
*Marinomonadaceae*	Genus *Marinomonas* van Landschoot and De Ley 1984	Ma.ri.no.mo.na.da.ce'ae. N.L. fem. n. *Marinomonas* type genus of the family; -*aceae* ending to denote a family; N.L. fem. pl. n. *Marinomonadaceae* the *Marinomonas* family	The family constitutes a monophyletic lineage as described and defined in Parks et al. (2018). The family contains the genus *Marinomonas*
*Marispirochaetaceae*	Genus *Marispirochaeta* Shivani et al. 2017	Ma.ri.spi.ro.chae.ta.ce'ae. N.L. fem. n. *Marispirochaeta* type genus of the family; -*aceae* ending to denote a family; N.L. fem. pl. n. *Marispirochaetaceae* the *Marispirochaeta* family	The family constitutes a monophyletic lineage as described and defined in Parks et al. (2018). The family contains the genus *Marispirochaeta*
*Megasphaeraceae*	Genus *Megasphaera* Rogosa 1971 (Approved Lists 1980)	Me.ga.sphae.ra.ce'ae. N.L. fem. n. *Megasphaera* type genus of the family; -*aceae* ending to denote a family; N.L. fem. pl. n. *Megasphaeraceae* the *Megasphaera* family	The family constitutes a monophyletic lineage as described and defined in Parks et al. (2018). The family contains the following genera: *Megasphaera*, ‘*Caecibacter*’, *Anaeroglobus*
*Methanoculleaceae*	Genus *Methanoculleus* Maestrojuán et al. 1990	Me.tha.no.cul.le.a.ce'ae. N.L. masc. n. *Methanoculleus* type genus of the family; -*aceae* ending to denote a family; N.L. fem. pl. n. *Methanoculleaceae* the *Methanoculleus* family	The family constitutes a monophyletic lineage as described and defined in Parks et al. (2018). The family contains the genus *Methanoculleus*
*Methanosphaerulaceae*	Genus *Methanosphaerula* Cadillo-Quiroz et al. 2009	Me.tha.no.sphae.ru.la.ce'ae. N.L. fem. n. *Methanosphaerula* type genus of the family; -*aceae* ending to denote a family; N.L. fem. pl. n. *Methanosphaerulaceae* the *Methanosphaerula* family	The family constitutes a monophyletic lineage as described and defined in Parks et al. (2018). The family contains the genus *Methanosphaerula*
*Methanothermobacteraceae*	Genus *Methanothermobacter* Wasserfallen et al. 2000	Me.tha.no.ther.mo.bac.te.ra.ce'ae. N.L. masc. n. *Methanothermobacter* type genus of the family; -*aceae* ending to denote a family; N.L. fem. pl. n. *Methanothermobacteraceae* the *Methanothermobacter* family	The family constitutes a monophyletic lineage as described and defined in Parks et al. (2018). The family contains the genus *Methanothermobacter*
*Methanofollaceae*	Genus *Methanofollis* Zellner et al. 1999	Me.tha.no.fol.la.ce'ae. N.L. masc. n. *Methanofollis* type genus of the family; -*aceae* ending to denote a family; N.L. fem. pl. n. *Methanofollaceae* the *Methanofollis* family	The family constitutes a monophyletic lineage as described and defined in Parks et al. (2018). The family contains the genus *Methanofollis*
*Methyloligellaceae*	Genus *Methyloligella* Doronina et al. 2014	Me.thyl.o.li.gel.la.ce'ae. N.L. fem. n. *Methyloligella* type genus of the family; -*aceae* ending to denote a family; N.L. fem. pl. n. *Methyloligellaceae* the *Methyloligella* family	The family constitutes a monophyletic lineage as described and defined in Parks et al. (2018). The family contains the following genera: *Methyloligella, Methyloceanibacter*
*Methylophagaceae*	Genus *Methylophaga* Janvier et al. 1985	Me.thy.lo.pha.ga.ce'ae. N.L. fem. n. *Methylophaga* type genus of the family; -*aceae* ending to denote a family; N.L. fem. pl. n. *Methylophagaceae* the *Methylophaga* family	The family constitutes a monophyletic lineage as described and defined in Parks et al. (2018). The family contains the genus *Methylophaga*
*Monoglobaceae*	Genus *Monoglobus* Kim et al. 2017	Mo.no.glo.ba.ce'ae. N.L. masc. n. *Monoglobus* type genus of the family; -*aceae* ending to denote a family; N.L. fem. pl. n. *Monoglobaceae* the *Monoglobus* family	The family constitutes a monophyletic lineage as described and defined in Parks et al. (2018). The family contains the genus *Monoglobus*
*Natronincolaceae*	Genus *Natronincola* corrig. Zhilina et al. 1999	Nat.ron.in.co.la.ce'ae. N.L. masc. n. *Natronincola* type genus of the family; -*aceae* ending to denote a family; N.L. fem. pl. n. *Natronincolaceae* the *Natronincola* family	The family constitutes a monophyletic lineage as described and defined in Parks et al. (2018). The family contains the following genera: *Natronincola, Alkaliphilus, Anaerovirgula, Serpentinicella*
*Negativicoccaceae*	Genus *Negativicoccus* Marchandin et al. 2010	Ne.ga.ti.vi.coc.ca.ce'ae. N.L. masc. n. *Negativicoccus* type genus of the family; -*aceae* ending to denote a family; N.L. fem. pl. n. *Negativicoccaceae* the *Negativicoccus* family	The family constitutes a monophyletic lineage as described and defined in Parks et al. (2018). The family contains the genus *Negativicoccus*
*Neiellaceae*	Genus *Neiella* Du et al. 2013	Nei.el.la.ce'ae. N.L. fem. n. *Neiella* type genus of the family; -*aceae* ending to denote a family; N.L. fem. pl. n. *Neiellaceae* the *Neiella* family	The family constitutes a monophyletic lineage as described and defined in Parks et al. (2018). The family contains the following genera: *Neiella, Corallincola*
*Nisaeaceae*	Genus *Nisaea* Urios et al. 2008	Ni.sae.a.ce'ae. N.L. fem. n. *Nisaea* type genus of the family; -*aceae* ending to denote a family; N.L. fem. pl. n. *Nisaeaceae* the *Nisaea* family	The family constitutes a monophyletic lineage as described and defined in Parks et al. (2018). The family contains the genus *Nisaea*
*Nitrococcaceae*	Genus *Nitrococcus* Watson and Waterbury 1971 (Approved Lists 1980)	Ni.tro.coc.ca.ce'ae. N.L. masc. n. *Nitrococcus* type genus of the family; -*aceae* ending to denote a family; N.L. fem. pl. n. *Nitrococcaceae* the *Nitrococcus* family	The family constitutes a monophyletic lineage as described and defined in Parks et al. (2018). The family contains the following genera: *Nitrococcus, Spiribacter, Arhodomonas*
*Nitrosococcaceae*	Genus *Nitrosococcus* Winogradsky 1892 (Approved Lists 1980)	Ni.tro.so.coc.ca.ce'ae. N.L. masc. n. *Nitrosococcus* type genus of the family; -*aceae* ending to denote a family; N.L. fem. pl. n. *Nitrosococcaceae* the *Nitrosococcus* family	The family constitutes a monophyletic lineage as described and defined in Parks et al. (2018). The family contains the following genera: *Nitrosococcus, Candidatus* Nitrosoglobus
*Oceanococcaceae*	Genus *Oceanococcus* Li et al. 2014	O.ce.a.no.coc.ca.ce'ae. N.L. masc. n. *Oceanococcus* type genus of the family; -*aceae* ending to denote a family; N.L. fem. pl. n. *Oceanococcaceae* the *Oceanococcus* family	The family constitutes a monophyletic lineage as described and defined in Parks et al. (2018). The family contains the following genera: *Oceanococcus, Abyssibacter*
*Oxobacteraceae*	Genus *Oxobacter* Collins et al. 1994	O.xo.bac.te.ra.ce'ae. N.L. masc. n. *Oxobacter* type genus of the family; -*aceae* ending to denote a family; N.L. fem. pl. n. *Oxobacteraceae* the *Oxobacter* family	The family constitutes a monophyletic lineage as described and defined in Parks et al. (2018). The family contains the genus *Oxobacter*
*Pelotomaculaceae*	Genus *Pelotomaculum* Imachi et al. 2002	Pe.lo.to.ma.cu.la.ce'ae. N.L. fem. n. *Pelotomaculum* type genus of the family; -*aceae* ending to denote a family; N.L. fem. pl. n. *Pelotomaculaceae* the *Pelotomaculum* family	The family constitutes a monophyletic lineage as described and defined in Parks et al. (2018). The family contains the genus *Pelotomaculum*
*Phormidesmaceae* (corrig. of ‘*Phormidesmiaceae’*)	Genus *Phormidesmis* Turicchia et al. 2009	Phor.mi.des.ma.ce'ae. N.L. fem. n. *Phormidesmis* type genus of the family; -*aceae* ending to denote a family; N.L. fem. pl. n. *Phormidesmaceae* the *Phormidesmis* family	The family constitutes a monophyletic lineage as described and defined in Parks et al. (2018). The family contains the following genera: *Phormidesmis, Nodosilinea, Halomicronema*
*Propionisporaceae*	Genus *Propionispora* Biebl et al. 2001	Pro.pi.o.ni.spo.ra.ce'ae. N.L. fem. n. *Propionispora* type genus of the family; -*aceae* ending to denote a family; N.L. fem. pl. n. *Propionisporaceae* the *Propionispora* family	The family constitutes a monophyletic lineage as described and defined in Parks et al. (2018). The family contains the following genera: *Propionispora, Pelosinus*
*Proteiniboraceae*	Genus *Proteiniborus* Niu et al. 2008	Pro.te.i.ni.bo.ra.ce'ae. N.L. masc. n. *Proteiniborus* type genus of the family; -*aceae* ending to denote a family; N.L. fem. pl. n. *Proteiniboraceae* the *Proteiniborus* family	The family constitutes a monophyletic lineage as described and defined in Parks et al. (2018). The family contains the genus *Proteiniborus*
*Pseudohongiellaceae*	Genus *Pseudohongiella* Wang et al. 2015	Pseu.do.hong.i.el.la.ce'ae. N.L. fem. n. *Pseudohongiella* type genus of the family; -*aceae* ending to denote a family; N.L. fem. pl. n. *Pseudohongiellaceae* the *Pseudohongiella* family	The family constitutes a monophyletic lineage as described and defined in Parks et al. (2018). The family contains the genus *Pseudohongiella*
*Quadrisphaeraceae*	Genus *Quadrisphaera* Maszenan et al. 2005	Qua.dri.sphae.ra.ce'ae. N.L. fem. n. *Quadrisphaera* type genus of the family; -*aceae* ending to denote a family; N.L. fem. pl. n. *Quadrisphaeraceae* the *Quadrisphaera* family	The family constitutes a monophyletic lineage as described and defined in Parks et al. (2018). The family contains the following genera: *Quadrisphaera, Pseudokineococcus*
*Rhodomicrobiaceae*	Genus *Rhodomicrobium* Duchow and Douglas 1949 (Approved Lists 1980)	Rho.do.mi.cro.bi.a.ce'ae. N.L. neut. n. *Rhodomicrobium* type genus of the family; -*aceae* ending to denote a family; N.L. fem. pl. n. *Rhodomicrobiaceae* the *Rhodomicrobium* family	The family constitutes a monophyletic lineage as described and defined in Parks et al. (2018). The family contains the following genera: *Rhodomicrobium, Dichotomicrobium*
*Rubidibacteraceae*	Genus *Rubidibacter* Choi et al. 2008	Ru.bi.di.bac.te.ra.ce'ae. N.L. masc. n. *Rubidibacter* type genus of the family; -*aceae* ending to denote a family; N.L. fem. pl. n. *Rubidibacteraceae* the *Rubidibacter* family	The family constitutes a monophyletic lineage as described and defined in Parks et al. (2018). The family contains the following genera: *Rubidibacter, Halothece*
*Salinicoccaceae*	Genus *Salinicoccus* Ventosa et al. 1990	Sa.li.ni.coc.ca.ce'ae. N.L. masc. n. *Salinicoccus* type genus of the family; -*aceae* ending to denote a family; N.L. fem. pl. n. *Salinicoccaceae* the *Salinicoccus* family	The family constitutes a monophyletic lineage as described and defined in Parks et al. (2018). The family contains the following genera: *Salinicoccus, Aliicoccus, Nosocomiicoccus, Jeotgalicoccus*
*Salinispiraceae*	Genus *Salinispira* (Ben Hania et al. 2015)	Sa.li.ni.spi.ra.ce'ae. N.L. fem. n. *Salinispira* type genus of the family; -*aceae* ending to denote a family; N.L. fem. pl. n. *Salinispiraceae* the *Salinispira* family	The family constitutes a monophyletic lineage as described and defined in Parks et al. (2018). The family contains the genus *Salinispira*
*Salisediminibacteriaceae*	Genus *Salisediminibacterium* Jiang et al. 2012	Sa.li.se.di.mi.ni.bac.te.ri.a.ce'ae. N.L. masc. n. *Salisediminibacterium* type genus of the family; -*aceae* ending to denote a family; N.L. fem. pl. n. *Salisediminibacteriaceae* the *Salisediminibacterium* family	The family constitutes a monophyletic lineage as described and defined in Parks et al. (2018). The family contains the following genera: *Salisediminibacterium, Alkalicoccus, Salipaludibacillus, Texcoconibacillus*
*Sedimentibacteraceae*	Genus *Sedimentibacter* Breitenstein et al. 2002	Se.di.men.ti.bac.te.ra.ce'ae. N.L. masc. n. *Sedimentibacter* type genus of the family; -*aceae* ending to denote a family; N.L. fem. pl. n. *Sedimentibacteraceae* the *Sedimentibacter* family	The family constitutes a monophyletic lineage as described and defined in Parks et al. (2018). The family contains the genus *Sedimentibacter*
*Sediminispirochaetaceae*	Genus *Sediminispirochaeta* Shivani et al. 2016	Se.di.mi.ni.spi.ro.chae.ta.ce'ae. N.L. fem. n. *Sediminispirochaeta* type genus of the family; -*aceae* ending to denote a family; N.L. fem. pl. n. *Sediminispirochaetaceae* the *Sediminispirochaeta* family	The family constitutes a monophyletic lineage as described and defined in Parks et al. (2018). The family contains the genus *Sediminispirochaeta*
*Succinispiraceae*	Genus *Succinispira* Janssen and O’Farrell 1999	Suc.ci.ni.spi.ra.ce'ae. N.L. fem. n. *Succinispira* type genus of the family; -*aceae* ending to denote a family; N.L. fem. pl. n. *Succinispiraceae* the *Succinispira* family	The family constitutes a monophyletic lineage as described and defined in Parks et al. (2018). The family contains the genus *Succinispira*
*Sulfuriferulaceae*	Genus *Sulfuriferula* Watanabe et al. 2015	Sul.fu.ri.fe.ru.la.ce'ae. N.L. fem. n. *Sulfuriferula* type genus of the family; -*aceae* ending to denote a family; N.L. fem. pl. n. *Sulfuriferulaceae* the *Sulfuriferula* family	The family constitutes a monophyletic lineage as described and defined in Parks et al. (2018). The family contains the genus *Sulfuriferula*
*Sulfurifustaceae*	Genus *Sulfurifustis* Kojima et al. 2015	Sul.fu.ri.fus.ta.ce'ae. N.L. masc. n. *Sulfurifustis* type genus of the family; -*aceae* ending to denote a family; N.L. fem. pl. n. *Sulfurifustaceae* the *Sulfurifustis* family	The family constitutes a monophyletic lineage as described and defined in Parks et al. (2018). The family contains the following genera: *Sulfurifustis, Sulfuricaulis*
*Syntrophothermaceae*	Genus *Syntrophothermus* Sekiguchi et al. 2000	Syn.tro.pho.ther.ma.ce'ae. N.L. masc. n. *Syntrophothermus* type genus of the family; -*aceae* ending to denote a family; N.L. fem. pl. n. *Syntrophothermaceae* the *Syntrophothermus* family	The family constitutes a monophyletic lineage as described and defined in Parks et al. (2018). The family contains the genus *Syntrophothermus*
*Tepidibacillaceae*	Genus *Tepidibacillus* Slobodkina et al. 2014	Te.pi.di.ba.cil.la.ce'ae. N.L. masc. n. *Tepidibacillus* type genus of the family; -*aceae* ending to denote a family; N.L. fem. pl. n. *Tepidibacillaceae* the *Tepidibacillus* family	The family constitutes a monophyletic lineage as described and defined in Parks et al. (2018). The family contains the following genera: *Tepidibacillus, Vulcanibacillus*
*Thermacetogeniaceae*	Genus *Thermacetogenium* Hattori et al. 2000	Therm.a.ce.to.ge.ni.a.ce'ae. N.L. neut. n. *Thermacetogenium* type genus of the family; -aceae ending to denote a family; N.L. fem. pl. n. *Thermacetogeniaceae* the *Thermacetogenium* family	The family constitutes a monophyletic lineage as described and defined in Parks et al. (2018). The family contains the following genera: *Thermacetogenium, Syntrophaceticus*
*Thermaerobacteraceae*	Genus *Thermaerobacter* Takai et al. 1999	Therm.a.e.ro.bac.te.ra.ce'ae. N.L. masc. n. *Thermaerobacter* type genus of the family; -*aceae* ending to denote a family; N.L. fem. pl. n. *Thermaerobacteraceae* the *Thermaerobacter* family	The family constitutes a monophyletic lineage as described and defined in Parks et al. (2018). The family contains the genus *Thermaerobacter*
*Thermicanaceae*	Genus *Thermicanus* Gößner et al. 2000	Ther.mi.ca.na.ce'ae. N.L. masc. n. *Thermicanus* type genus of the family; -*aceae* ending to denote a family; N.L. fem. pl. n. *Thermicanaceae* the *Thermicanus* family	The family constitutes a monophyletic lineage as described and defined in Parks et al. (2018). The family contains the genus *Thermicanus*
*Thermocladiaceae*	Genus *Thermocladium* Itoh et al. 1998	Ther.mo.cla.di.a.ce'ae. N.L. neut. n. *Thermocladium* type genus of the family; -*aceae* ending to denote a family; N.L. fem. pl. n. *Thermocladiaceae* the *Thermocladium* family	The family constitutes a monophyletic lineage as described and defined in Parks et al. (2018). The family contains the following genera: *Thermocladium, Caldivirga, Vulcanisaeta*
*Thermosinaceae*	Genus *Thermosinus* Sokolova et al. 2004	Ther.mo.si.na.ce'ae. N.L. masc. n. *Thermosinus* type genus of the family; -*aceae* ending to denote a family; N.L. fem. pl. n. *Thermosinaceae* the *Thermosinus* family	The family constitutes a monophyletic lineage as described and defined in Parks et al. (2018). The family contains the following genera: *Thermosinus, Sporolituus, Anaerospora*
*Thermosulfidibacteraceae*	Genus *Thermosulfidibacter* Nunoura et al. 2008	Ther.mo.sul.fi.di.bac.te.ra.ce'ae. N.L. masc. n. *Thermosulfidibacter* type genus of the family; -*aceae* ending to denote a family; N.L. fem. pl. n. *Thermosulfidibacteraceae* the *Thermosulfidibacter* family	The family constitutes a monophyletic lineage as described and defined in Parks et al. (2018). The family contains the genus *Thermosulfidibacter*
*Thermotaleaceae*	Genus *Thermotalea* Ogg and Patel 2009	Ther.mo.ta.le.a.ce'ae. N.L. fem. n. *Thermotalea* type genus of the family; -*aceae* ending to denote a family; N.L. fem. pl. n. *Thermotaleaceae* the *Thermotalea* family	The family constitutes a monophyletic lineage as described and defined in Parks et al. (2018). The family contains the following genera: *Thermotalea, Anaeromicrobium, Anaerosolibacter, Geosporobacter, “Inediibacterium”, Marinisporobacter*
*Thioalkalivibrionaceae*	Genus *Thioalkalivibrio* corrig. Sorokin et al. 2001	Thi.o.al.ka.li.vib.rio.na.ce'ae. N.L. masc. n. *Thioalkalivibrio* type genus of the family; -*aceae* ending to denote a family; N.L. fem. pl. n. *Thioalkalivibrionaceae* the *Thioalkalivibrio* family	The family constitutes a monophyletic lineage as described and defined in Parks et al. (2018). The family contains the genus *Thioalkalivibrio*
*Tindalliaceae*	Genus *Tindallia* Kevbrin et al. 1999	Tin.dal.li.a.ce'ae. N.L. fem. n. *Tindallia* type genus of the family; -*aceae* ending to denote a family; N.L. fem. pl. n. *Tindalliaceae* the *Tindallia* family	The family constitutes a monophyletic lineage as described and defined in Parks et al. (2018). The family contains the genus *Tindallia*
*Tistrellaceae*	Genus *Tistrella* Shi et al. 2003	Tis.trel.la.ce'ae. N.L. fem. n. *Tistrella* type genus of the order; -*aceae* ending to denote a family; N.L. fem. pl. n. *Tistrellaceae* the *Tistrella* family	The family constitutes a monophyletic lineage as described and defined in Parks et al. (2018). The family contains the genus *Tistrella*
*Tumebacillaceae*	Genus *Tumebacillus* Steven et al. 2008	Tu.me.ba.cil.la.ce'ae. N.L. masc. n. *Tumebacillus* type genus of the family; -*aceae* ending to denote a family; N.L. fem. pl. n. *Tumebacillaceae* the *Tumebacillus* family	The family constitutes a monophyletic lineage as described and defined in Parks et al. (2018). The family contains the genus *Tumebacillus*
*Turneriellaceae*	Genus *Turneriella* Levett et al. 2005	Tur.ne.ri.el.la.ce'ae. N.L. fem. n. *Turneriella* type genus of the family; -*aceae* ending to denote a family; N.L. fem. pl. n. *Turneriellaceae* the *Turneriella* family	The family constitutes a monophyletic lineage as described and defined in Parks et al. (2018). The family contains the genus *Turneriella*
*Vagococcaceae*	Genus *Vagococcus* Collins et al. 1990	Va.go.coc.ca.ce'ae. N.L. masc. n. *Vagococcus* type genus of the family; -*aceae* ending to denote a family; N.L. fem. pl. n. *Vagococcaceae* the *Vagococcus* family	The family constitutes a monophyletic lineage as described and defined in Parks et al. (2018). The family contains the genus *Vagococcus*
*Vampirovibrionaceae*	Genus *Vampirovibrio* Gromov and Mamkayeva 1980	Vam.pi.ro.vi.bri.o.na.ce'ae. N.L. masc. n. *Vampirovibrio* type genus of the family; -*aceae* ending to denote a family; N.L. fem. pl. n. *Vampirovibrionaceae* the *Vampirovibrio* family	The family constitutes a monophyletic lineage as described and defined in Parks et al. (2018). The family contains the genus *Vampirovibrio*
*Wohlfahrtiimonadaceae*	Genus *Wohlfahrtiimonas* Tóth et al. 2008	Wohl.fahr.ti.i.mo.na.da.ce'ae. N.L. fem. n. *Wohlfahrtiimonas* type genus of the family; -*aceae* ending to denote a family; N.L. fem. pl. n. *Wohlfahrtiimonadaceae* the *Wohlfahrtiimonas* family	The family constitutes a monophyletic lineage as described and defined in Parks et al. (2018). The family contains the following genera: *Wohlfahrtiimonas, Ignatzschineria*

1 Type denotes nomenclature type as defined under ICNP.

2 Membership is based on release R07-RS207 of GTDB and only named members are listed.

3 Novel taxon proposed as part of this manuscript.

## Materials and methods

### Identification of names lacking published taxonomic descriptions

To identify GTDB provisional names that are missing published taxonomic descriptions, we first screened the widely used taxonomic and nomenclature databases—NCBI and LPSN. A set of taxonomic names that stably occurred between release R01-RS80 and R07-RS207 were matched to the list of names obtained from NCBI and LPSN using a custom python script. Names applied to the rank of species (thus, including new combinations) were excluded. Names of higher taxa that appear only in GTDB, were treated as GTDB provisional names and were subjected to additional manual checks to ensure that they have not been published elsewhere by performing searches in Google Scholar and Web of Science. Additionally, GTDB names that were present in LPSN but missing in NCBI were manually checked for publication since LPSN has started to list names that appear in the literature without author citation (i.e. with unknown authorship such as Zakham et al. (2019), as described above). A final set of GTDB provisional higher taxa names was categorized by rank and nomenclature status of the genera that were selected earlier as provisional types for naming taxa.

### Selection of type genome sequences

To select taxonomic names that can be proposed for validation under the SeqCode, names with the status *Candidatus* were screened for the presence of high-quality genomes that could serve as type material for the type species of the selected genera. For this, metadata containing assembly statistics, completeness and contamination, and genomic properties were compiled from the GTDB repository of release R07-RS207, for each selected *Candidatus* species. High quality genome sequences (estimated completeness > 90% and contamination < 5%) were checked for additional criteria recommended for a genome sequence to serve as type under the SeqCode, e.g. presence of 16S rRNA genes (Hedlund et al. 2022). Genomes meeting these minimal quality standards were then examined for agreement between genome and 16S rRNA-based taxonomic assignments via phylogenetic analyses.

### 16S rRNA gene phylogeny of type genome sequences

16S rRNA gene sequences were identified and extracted from the selected type genomes using nhmmer v3.1b2 with the 16S rRNA model (RF00177) from the RFAM database. The dataset was supplemented with additional sequences obtained from closely related species. Sequences were aligned using ssu-align v0.1.1, and trimmed using trimal v1.4.rev15 followed by a custom trimming script to remove low parsimony columns. Phylogenetic trees were constructed using IQ-TREE v.2.2.0.3 with the following parameters: iqtree -s -m MFP -T 10 -alrt 1000. Trees were rooted with the closest outgroup and visualized in Dendroscope v3.8.8. A detailed workflow is provided at https://doi.org/10.5281/zenodo.7992507.

### Concatenated protein phylogeny

Although taxa described in this manuscript were delineated earlier as described in Parks et al. (2018) and phylogenetic trees are provided in the GTDB repository (https://gtdb.ecogenomic.org/downloads), additional genome-based subtrees were inferred to support taxonomic descriptions. For this, bacterial genomes corresponding to GTDB representative genomes of named species (type strains or selected type genome sequences) of each proposed taxon were obtained from RefSeq/GenBank release 207. Genome trees were constructed from a multiple sequence alignment of concatenated protein markers as described in Parks et al. (2018) using FastTree 2.1.11 (Price et al. 2010) with the WAG model as implemented in GTDB-Tk v2.1.0 (Chaumeil et al. 2022). These trees were then used as starting trees to infer final trees with IQ-TREE v.2.1.2 (Nguyen et al. 2015) using the PMSF approximation. Trees inferred with IQ-TREE were executed with the following parameters: -m LG + C10 + F + G -ft < starting_tree> -alrt 1000. Trees were rooted by the closest outgroup, visualized in ARB v.6.0.6 and beautified in iTOL (Letunic and Bork 2019).

### Comparison of taxonomic assignments of type genomes

To verify the taxonomic assignment of type genome sequences, 16S rRNA gene phylogenies were compared to the concatenated protein phylogenies (inferred as described above) of the same taxa using the Tanglegram algorithm in Dendroscope v3.8.8, then annotated using Adobe Illustrator.

## Results and discussion

### Nomenclatural consequences of the GTDB taxonomic framework

After searching the literature for effective publications of provisional GTDB higher taxon names, 284 bacterial and 45 archaeal taxa still lack formal nomenclatural proposals. Of these, 223 (68%) have the potential to be validated according to the ICNP because they are underpinned by type strains that are serving as type material for species of genera proposed here as nomenclature types of higher taxa (Oren et al. 2023; Table 1; Figure S1, Supporting Information). The remaining 106 (32%) provisional GTDB names are based on *Candidatus* taxa that lack cultured representatives and therefore are not covered by the rules of the ICNP (Murray and Schleifer 1994, Murray and Stackebrandt 1995). However, there is the potential to propose at least some of these names under the newly developed Code of Nomenclature of Prokaryotes described from Sequence Data, or SeqCode for short. SeqCode is an alternative code for prokaryotic taxa based on high quality sequence data rather than axenic cultures as type material providing the ability to validly name uncultured taxa (Hedlund et al. 2022, Whitman et al. 2022; https://seqco.de). Here, we designate type genome sequences, which satisfy the SeqCode data quality recommendations, for 23 published *Candidatus* species names to validate both genus and species names under the SeqCode, at which time the *Candidatus* prefix can be removed (Whitman et al. 2022; Table 2; Table S1 and Figure S1 and S19, Supporting Information). These provide the basis for 49 of the 106 (46%) provisional GTDB names based on *Candidatus* taxa (Table 3; Figure S1, Supporting Information). An additional 57 higher taxon names based on *Candidatus* species that do not satisfy the typification and other validation criteria of either code (e.g. no axenic cultures deposited in two culture collections in different countries, low quality genome sequence) are described as new *Candidatus* taxa (Table 4; Figure S1, Supporting Information). Note that we have chosen to propose higher taxon names under the ICNP where possible resulting in a much higher number of names proposed under this code than the SeqCode (223 vs. 49). This is because the SeqCode recognizes names validly published under the ICNP (Whitman et al. 2022), but not *vice versa*, thereby minimizing the possibility of future synonyms if SeqCode names are subsequently proposed under the ICNP.

**Table 2.** List of genera and their type species based on genome sequences as nomenclature types proposed according to the SeqCode.

Taxon name	Rank	Type^1^	Etymology	Description
*Azobacteroides*	gen. nov	Species *Azobacteroides pseudotrichonymphae* Hongoh et al. 2008	A.zo.bac.ter.oi'des. N.L. neut. n. *azotum*, nitrogen; from French masc. n. *azote*, nitrogen; from Gr. pref. *a-*, not; from Gr. fem. n. *zôê*, life; from N.Gr. fem. n. *azôê*, not sustaining life; N.L. masc. n. *Bacteroides*, a bacterial genus; N.L. masc. n. *Azobacteroides*, a nitrogen (fixing) *Bacteroides*	The description is the same as given by Hongoh et al. (2008)
*Azobacteroides pseudotrichonymphae*	sp. nov	Genomic assembly: GCA_000010645.1	pseu.do.tri.cho.nym'phae. N.L. gen. fem. n. *pseudotrichonymphae*, of the flagellate protist genus *Pseudotrichonympha*	The description is the same as given by Hongoh et al. (2008)
*Binatus*	gen. nov	Species *Binatus soli* Chuvochina et al. 2019	Bi.na'tus. L. adv. *bis*, twice; L. masc. adj. *natus*, born; N.L. masc. n. *Binatus*, born twice	The description is the same as given by Chuvochina et al. (2019)
*Binatus soli*	sp. nov	Genomic assembly: GCA_002479255.1	so'li. L. gen. neut. n. *soli*, of soil	The description is the same as given by Chuvochina et al. (2019)
*Bipolaricaulis*	gen. nov	Species *Bipolaricaulis anaerobius* Hao et al. 2018	Bi.po.la.ri.cau'lis. L. adv. *bis*, twice; M.L. masc./fem. adj. *polaris*, polar, pertaining to the poles of the rod-, shaped cell; L. masc. n. *caulis*, a stalk; N.L. masc. n. *Bipolaricaulis*, stalks at both poles	The description is the same as given by Hao et al. (2018)
*Bipolaricaulis anaerobius*	sp. nov	Genomic assembly: GCA_900465355.1	an.a.e.ro'bi.us. Gr. pref. *an-*, not; Gr. masc. n. *aêr (gen. aeros)*, air; Gr. masc. n. *bios*, life;; N.L. masc. adj. *anaerobius*, not living in air, anaerobic	The description is the same as given by Hao et al. (2018)
*Cloacimonas*	gen. nov	Species *Cloacimonas acidaminivorans*^2^*corrig*. Pelletier et al. 2008	Clo.a.ci.mo'nas. L. fem. n. *cloaca* sewer; L. fem. n. *monas* unit, monad; N.L. fem. n. *Cloacimonas* a monad from a sewer	The description is the same as given by Pelletier et al. (2008)
*Cloacimonas acidaminivorans*	sp. nov	Genomic assembly: GCA_000146065.1	a.cid.a.mi.ni.vo'rans. N.L. neut. n. *acidum aminum* amino acid; L. pres. part. *vorans* devouring; N.L. part. adj. *acidaminivorans* amino acid- devouring	The description is the same as given by Pelletier et al. (2008)
*Desulforudis*	gen. nov	Species *Desulforudis audaxviator* Chivian et al. 2008	De.sul.fo.ru'dis. L. prep. *de*, from; N.L. pref. *sulfo-*, pertaining to sulfur; from N.L. masc. n. *sulfas (gen. sulfatis)*, sulfate; L. masc./fem. adj. *rudis*, a slender rod; N.L. fem. n. *Desulforudis*, a sulfate-reducing slender rod	The description is the same as given by Chivian et al. (2008)
*Desulforudis audaxviator*	sp. nov	Genomic assembly: GCA_000018425.1	au.dax.vi.a'tor. L. adj. *audax*, daring, courageous; L. masc. n. *viator*, traveler; N.L. masc. n. *audaxviator*, arbitrary name, courageous traveler	The description is the same as given by Chivian et al. (2008)
*Hadarchaeum*	gen. nov	Species *Hadarchaeum yellowstonense* Chuvochina et al. 2019	Had.ar.chae'um. Gr. masc. n. *Haidês*, Hades, the underworld; N.L. neut. n. *archaeum*, archaeon; N.L. neut. n. *Hadarchaeum*, archaeon from the subsurface	The description is the same as given by Chuvochina et al. (2019)
*Hadarchaeum yellowstonense*	sp. nov	Genomic assembly: GCA_001515205.2	yel.low.ston.en'se. N.L. neut. adj. *yellowstonense*, pertaining to Yellowstone	The description is the same as given by Chuvochina et al. (2019)
*Hepatobacter*	gen. nov	Species *Hepatobacter penaei* Nunan et al. 2013	He.pa.to.bac'ter. Gr. neut. n. *hepar, hepatos* liver; N.L. masc. n. *bacter* a rod; N.L. masc. n. *Hepatobacter* a rod from the liver	The description is the same as given by Nunan et al. (2013)
*Hepatobacter penaei*	sp. nov	Genomic assembly: GCA_000742475.1	pe.nae'i. N.L. gen. n. *penaei* of the prawn genus *Penaeus*	The description is the same as given by Nunan et al. (2013)
*Hepatoplasma*	gen. nov	Species *Hepatoplasma crinochetorum* Wang et al. 2004	He.pa.to.plas'ma. Gr. neut. n. *hepar, hepatos* liver; Gr. neut. n. *plasma* anything formed or moulded, image, figure; N.L. neut. n. *Hepatoplasma* a form from the liver	The description is the same as given by Wang et al. (2004)
*Hepatoplasma crinochetorum*	sp. nov	Genomic assembly: GCA_000582535.1	cri.no.che.to'rum. N.L. gen. pl. n. *crinochetorum* of isopods (*Crinocheta*)	The description is the same as given by Wang et al. (2004)
*Hydrothermarchaeum*	gen. nov	Species *Hydrothermarchaeum profundi* Chuvochina et al. 2019	Hy.dro.therm.ar.chae'um. Gr. neut. n. *hydôr*, water; Gr. masc. adj. *thermos*, hot; N.L. neut. n. *archaeum*, archaeon; N.L. neut. n. *Hydrothermarchaeum*, an archaeon from a hydrothermal environment	The description is the same as given by Chuvochina et al. (2019)
*Hydrothermarchaeum profundi*	sp. nov	Genomic assembly: GCA_002011125.1	pro.fun'di. L. gen. neut. n. *profundi*, from the depth of the sea	The description is the same as given by Chuvochina et al. (2019)
*Hydrothermus*	gen. nov	Species *Hydrothermus pacificus* Chuvochina et al. 2019	Hy.dro.ther'mus. Gr. neut. *hydôr* or water; Gr. masc. adj. *thermos* hot; N.L. masc. n. *Hydrothermus* an organism living in hot water	The description is the same as given by Chuvochina et al. (2019)
*Hydrothermus pacificus*	sp. nov	Genomic assembly: GCA_002011615.1	pa.ci'fi.cus. L. masc. adj. *pacificus* peaceful, pertaining to Pacific Ocean	The description is the same as given by Chuvochina et al. (2019)
*Johnevansia*	gen. nov	Species *Johnevansia muelleri*^3^ corrig Kuechler et al. 2013	John.e.van'si.a. N.L. fem. n. *Johnevansia*, named after John William Evans for his pioneering work on bacteriomes in moss bugs	The description is the same as given by Kuechler et al. (2013)
*Johnevansia muelleri*	sp. nov	Genomic assembly: GCA_000953435.1	muel'le.ri. N.L. gen. masc. n. *muelleri*, referring to Mueller	The description is the same as given by Kuechler et al. (2013)
*Kapaibacterium*	gen. nov	Species *Kapaibacterium thiocyanatum*^3^*corrig*. Kantor et al. 2015	Ka.pa.i.bac.te'ri.um. *Kapa*, based on Motse Kapa, the name of Cape Town in the Sesotho language; N.L. neut. n. *bacterium*, a rod; N.L. neut. n. *Kapaibacterium*, a rod from Cape Town	The description is the same as given by Kantor et al. (2015)
*Kapaibacterium thiocyanatum*	sp. nov	Genomic assembly: GCA_001899175.1	thi.o.cy.a.na'tum. N.L. neut. adj. *thiocyanatum*, pertaining to thiocyanate	The description is the same as given by Kantor et al. (2015)
*Magnetobacterium*	gen. nov	Species *Magnetobacterium casense*^2^ corrig. Spring et al. 1993	Ma.gne.to.bac.te′ri.um. Gr. n. *magnes*, - *etos* a magnet; N.L. pref. *magneto*- pertaining to a magnet; N.L. neut. n. *bacterium* a rod; N.L. neut. n. *Magnetobacterium* a magnetic rod	The description is the same as given by Spring et al. (1993) with emendation by Lin et al. (2014)
*Magnetobacterium casense*	sp. nov	Genomic assembly: GCA_000714715.1	cas.en'se. N.L. neut. adj. *casense*, pertaining to CAS, acronym for the Chinese Academy of Sciences	The description is the same as given by Lin et al. (2014)
*Methylomirabilis*	gen. nov	Species *Methylomirabilis oxygeniifera*^2^*corrig*. Ettwig et al. 2010	Me.thy.lo.mi.ra'bi.lis. N.L. pref. *methylo*- pertaining to the methyl group; L. fem. adj. *mirabilis* wonderful; N.L. fem. n. *Methylomirabilis* a wonderful methyl (group oxidizing) organism	The description is the same as given by Ettwig et al. (2010)
*Methylomirabilis oxygeniifera*	sp. nov	Genomic assembly: GCA_000091165.1	o.xy.ge.ni.i'fe.ra. N.L. neut. n. *oxygenium*, oxygen; L. v. *fero*, to produce, to bear; N.L. fem. adj. *oxygeniifera*, carrying oxygen	The description is the same as given by Ettwig et al. (2010)
*Muiribacterium*	gen. nov	Species *Muiribacterium halophilum*^3^*corrig*. Barnum et al. 2018	Mui.ri.bac.te'ri.um. N.L. neut. n. *bacterium*, a rod; N.L. neut. n. *Muiribacterium*, a rod named after John Muir, the American conservationist for his contributions to the protection of natural areas in California	The description is the same as given by Barnum et al. (2018)
*Muiribacterium halophilum*	sp. nov	Genomic assembly: GCA_002869225.1	ha.lo'phi.lum. Gr. masc. n. *hals*, salt; N.L. neut. adj. suff. *-philum*, loving; from Gr. neut. adj. *philon*, friend; N.L. neut. adj. *halophilum*, salt loving	The description is the same as given by Barnum et al. (2018)
*Nucleicultrix*	gen. nov	Species *Azobacteroides pseudotrichonymphae* Schulz et al. 2014	Nu.cle.i.cul'trix. L. masc. n. *nucleus*, a little nut and in biology, a nucleus; L. fem. n. *cultrix*, inhabitant; N.L. fem. n. *Nucleicultrix*, inhabitant of the nucleus	The description is the same as given by Schulz et al. (2014)
*Nucleicultrix amoebiphila*	sp. nov	Genomic assembly: GCA_002117145.1	a.moe.bi'phi.la. N.L. fem. n. *amoeba*, an amoeba; N.L. fem. adj. suff. *-phila*, friend, loving; N.L. fem. adj. *amoebiphila*, amoeba-loving	The description is the same as given by Schulz et al. (2014)
*Obscuribacter*	gen. nov	Species *Obscuribacter phosphatis* Soo et al. 2014	Ob.scu.ri.bac'ter. L. masc. adj. *obscurus*, dark; N.L. masc. n. *bacter*, a rod; from Gr. neut. n. *baktron*, rod; N.L. masc. n. *Obscuribacter*, a bacterium found in the dark	The description is the same as given by Soo et al. (2014)
*Obscuribacter phosphatis*	sp. nov	Genomic assembly: GCA_001899315.1	phos.pha'tis. N.L. gen. n. *phosphatis*, of phosphate, accumulating phosphate	The description is the same as given by Soo et al. (2014)
*Promineifilum*	gen. nov	Species *Promineifilum breve*^3^*corrig*. Mcllroy et al. 2016	Pro.mi.ne.i.fi'lum. L. v. *promineo*, to project, to jut out; L. neut. n. *filum*, a thread; N.L. neut. n. *Promineifilum*, a protruding thread	The description is the same as given by Mcllroy et al. (2016)
*Promineifilum breve*	sp. nov	Genomic assembly: GCA_900066015.1	bre've. L. neut. adj. *breve*, short	The description is the same as given by Mcllroy et al. (2016)
*Pseudothioglobus*	gen. nov	Species *Pseudothioglobus singularis* Van Vliet et al. 2021	Pseu.do.thi.o.glo'bus. Gr. neut. adj. *pseudes*, false; Gr. neut. n. *theîon*, sulfur; L. masc. n. *globus*, ball, sphere; N.L. masc. n. *Pseudothioglobus*, false sulfur-oxidizing sphere	The description is the same as given by Van Vliet et al. (2021)
*Pseudothioglobus singularis*	sp. nov	Genomic assembly: GCA_001281385.1	sin.gu.la'ris. L. masc. adj. *singularis*, alone, singular	The description is the same as given by Van Vliet et al. (2021)
*Puniceispirillum*	gen. nov	Species *Puniceispirillum marinum* Oh et al. 2010	Pu.ni.ce.i.spi.ril'lum. L. masc. adj. *puniceus* reddish; N.L. neut. n. *spirillum* a little coil; N.L. neut. n. *Puniceispirillum* a little reddish coil	The description is the same as given by Oh et al. (2010)
*Puniceispirillum marinum*	sp. nov	Genomic assembly: GCA_000024465.1	ma.ri'num. L. neut. adj. *marinum*, marine	The description is the same as given by Oh et al. (2010)
*Saccharimonas*	gen. nov	Species *Saccharimonas aalborgensis* Albertsen et al. 2013	Sac.cha.ri.mo'nas. N.L. neut. n. *saccharum*, sugar; L. fem. n. *monas*, unit, monad; N.L. fem. n. *Saccharimonas*, a monad associated with sugar	The description is the same as given by Albertsen et al. (2013)
*Saccharimonas aalborgensis*	sp. nov	Genomic assembly: GCA_000392435.1	aal.borg.en'sis. N.L. fem. adj. *aalborgensis*, pertaining to the city of Aalborg, where the sample containing the organism was obtained	The description is the same as given by Albertsen et al. (2013)
*Tenderia*	gen. nov	Species *Tenderia electrophaga* Eddie et al. 2016	Ten.de'ri.a. N.L. fem. n. *Tenderia* named after Leonard M. Tender, the pioneering researcher in the development of microbial electrochemical technologies	The description is the same as given by Eddie et al. (2016)
*Tenderia electrophaga*	sp. nov	Genomic assembly: GCA_001447805.1	e.lec.tro'pha.ga. Gr. neut. n. *electron* amber; Gr. v. *phago* to eat; N.L. fem. adj. *electrophaga* eater of electricity	The description is the same as given by Eddie et al. (2016)
*Thermobaculum*	gen. nov	Species *Thermobaculum terrenum* Botero et al. 2004	Ther.mo.ba'cu.lum. Gr. masc. adj. *thermos*, hot; L. neut. n. *baculum*, small rod; N.L. neut. n. *Thermobaculum*, hot small rod	The description is the same as given by Botero et al. (2004)
*Thermobaculum terrenum*	sp. nov	Genomic assembly: GCA_000025005.1	ter.re'num. L. neut. adj. *terrenum*, belonging to earth/soil	The description is the same as given by Botero et al. (2004)

1 Type denotes nomenclature type as defined under the SeqCode.

2 Name correction as suggested by Oren et al. (2017).

3 Name correction as suggested by Oren et al. (2020).

**Table 3.** Description of GTDB-defined new higher taxa based on type genera proposed according to the SeqCode (see Table 2). Names deposited in the SeqCode Registry under the accessions seqco.de/r:3yxqlvua and seqco.de/r:7ewkque5.

Taxon name	Type^1^	Etymology	Properties and Membership^2^
**Rank of Phylum (all proposed as phyl. nov.)**			
*Hadarchaeota*	Genus *Hadarchaeum* Chuvochina et al, 2019	Had.ar.chae.o'ta. N.L. neut. n. *Hadarchaeum* type genus of the phylum; -*ota* ending to denote a phylum; N.L. neut. pl. n. *Hadarchaeota* the *Hadarchaeum* phylum	The phylum constitutes a monophyletic lineage as described and defined in Rinke et al. (2021). The phylum contains the class *Hadarchaeia*.^3^
*Methylomirabilota*	Genus *Methylomirabilis* Ettwig et al. 2010	Me.thy.lo.mi.ra.bi.lo'ta. N.L. fem. n. *Methylomirabilis* type genus of the phylum; -*ota* ending to denote a phylum; N.L. neut. pl. n. *Methylomirabilota* the *Methylomirabilis* phylum	The phylum constitutes a monophyletic lineage as described and defined in Parks et al. (2018). The phylum contains the class *Methylomirabilia*.^3^
**Rank of Class (all proposed as class. nov.)**			
*Binatia* (name appeared first in Chuvochina et al. (2019) but the taxon was not proposed)	Genus *Binatus* Chuvochina et al. 2019	Bi.na'ti.a. N.L. masc. n. *Binatus* type genus of the class; -*ia* ending to denote a class; N.L. neut. pl. n. *Binatia* the *Binatus* class	The class constitutes a monophyletic lineage as described and defined in Parks et al. (2018). The class contains the order *Binatales*.^3^
*Bipolaricaulia*	Genus *Bipolaricaulis* Hao et al. 2018	Bi.po.la.ri.cau'li.a. N.L. masc. n. *Bipolaricaulis* type genus of the class; -*ia* ending to denote a class; N.L. neut. pl. n. *Bipolaricaulia* the *Bipolaricaulis* class	The class constitutes a monophyletic lineage as described and defined in Parks et al. (2018). The class contains the order *Bipolaricaulales*.^3^
*Cloacimonadia*	Genus *Cloacimonas* Pelletier et al. 2008	Clo.a.ci.mo.na'di.a. N.L. fem. n. *Cloacimonas* type genus of the class; -*ia* ending to denote a class; N.L. neut. pl. n. *Cloacimonadia* the *Cloacimonas* class	The class constitutes a monophyletic lineage as described and defined in Parks et al. (2018). The class contains the order *Cloacimonadales*.^3^
*Hadarchaeia*	Genus *Hadarchaeum* Chuvochina et al. 2019	Had.ar.chae'i.a. N.L. neut. n. *Hadarchaeum* type genus of the class; -*ia* ending to denote a class; N.L. neut. pl. n. *Hadarchaeia* the *Hadarchaeum* class	The class constitutes a monophyletic lineage as described and defined in Rinke et al. (2021). The class contains the order *Hadarchaeales*.^3^
*Hydrothermarchaeia*	Genus *Hydrothermarchaeum* Chuvochina et al. 2019	Hy.dro.therm.ar.chae'i.a. N.L. neut. n. *Hydrothermarchaeum* type genus of the class; -*ia* ending to denote a class; N.L. neut. pl. n. *Hydrothermarchaeia* the *Hydrothermarchaeum* class	The class constitutes a monophyletic lineage as described and defined in Rinke et al. (2021). The class contains the order *Hydrothermarchaeales*.^3^
*Hydrothermia* (name appeared first in Chuvochina et al. (2019) but the taxon was not proposed)	Genus *Hydrothermus* Chuvochina et al. 2019	Hy.dro.ther'mi.a. N.L. masc. n. *Hydrothermus* type genus of the class; -*ia* ending to denote a class; N.L. neut. pl. n. *Hydrothermia* the *Hydrothermus* class	The class constitutes a monophyletic lineage as described and defined in Parks et al. (2018). The class contains the order *Hydrothermales*.^3^
*Kapaibacteriia* (corrig. of ‘*Kapabacteria*’)	Genus *Kapaibacterium*^4^ corrig. (Kapabacteria, sic) Kantor et al. 2015	Ka.pa.i.bac.te.ri'i.a. N.L. neut. n. *Kapaibacterium* type genus of the class; -*ia* ending to denote a class; N.L. fem. pl. n. *Kapaibacteria* the *Kapaibacterium* class	The class constitutes a monophyletic lineage as described and defined in Parks et al. (2018). The class contains the order *Kapaibacteriales*.^3^
*Methylomirabilia*	Genus *Methylomirabilis* Ettwig et al. 2010	Me.thy.lo.mi.ra.bi'li.a. N.L. fem. n. *Methylomirabilis* type genus of the class; -*ia* ending to denote a class; N.L. neut. pl. n. *Methylomirabilia* the *Methylomirabilis* class	The class constitutes a monophyletic lineage as described and defined in Parks et al. (2018). The class contains the following orders: *Methylomirabilales*,^3^*Candidatus* Rokubacteriales.^4^
*Muiribacteriia* (corrig. of ‘*Muirbacteriia*’)	Genus *Muiribacterium*^5^ corrig. Barnum et al. 2018	Mui.ri.bac.te.ri'i.a. N.L. neut. n. *Muiribacterium* type genus of the class; -*ia* ending to denote a class; N.L. neut. pl. n. *Muiribacteriia* the *Muiribacterium* class	The class constitutes a monophyletic lineage as described and defined in Parks et al. (2018). The class contains the order *Muiribacteriales*.^3^
**Rank of Order (all proposed as ord. nov.)**			
*Binatales* (name appeared first in Chuvochina et al. (2019) but the taxon was not proposed)	Genus *Binatus* Chuvochina et al. 2019	Bi.na.ta'les. N.L. masc. n. *Binatus* type genus of the order; -*ales* ending to denote an order; N.L. fem. pl. n. *Binatales* the *Binatus* order	The order constitutes a monophyletic lineage as described and defined in Parks et al. (2018). The order contains the family *Binataceae*.^3^
*Bipolaricaulales*	Genus *Bipolaricaulis* Hao et al. 2018	Bi.po.la.ri.cau.la'les. N.L. masc. n. *Bipolaricaulis* type genus of the order; -*ales* ending to denote an order; N.L. fem. pl. n. *Bipolaricaulales* the *Bipolaricaulis* order	The order constitutes a monophyletic lineage as described and defined in Parks et al. (2018). The order contains the family *Bipolaricaulaceae*.^3^
*Cloacimonadales*	Genus *Cloacimonas* Pelletier et al. 2008	Clo.a.ci.mo.na.da'les. N.L. fem. n. *Cloacimonas* type genus of the order; -*ales* ending to denote an order; N.L. fem. pl. n. *Cloacimonadales* the *Cloacimonas* order	The order constitutes a monophyletic lineage as described and defined in Parks et al. (2018). The order contains the family *Cloacimonadaceae*.^3^
*Hadarchaeales*	Genus *Hadarchaeum* Chuvochina et al. 2019	Had.ar.chae.a'les. N.L. neut. n. *Hadarchaeum* type genus of the order; -*ales* ending to denote an order; N.L. fem. pl. n. *Hadarchaeales* the *Hadarchaeum* order	The order constitutes a monophyletic lineage as described and defined in Rinke et al. (2021). The order contains the family *Hadarchaeaceae*.^3^
*Hydrothermarchaeales*	Genus *Hydrothermarchaeum* Chuvochina et al. 2019	Hy.dro.therm.ar.chae.a'les. N.L. neut. n. *Hydrothermarchaeum* type genus of the order; -*ales* ending to denote an order; N.L. fem. pl. n. *Hydrothermarchaeales* the *Hydrothermarchaeum* order	The order constitutes a monophyletic lineage as described and defined in Rinke et al. (2021). The order contains the family *Hydrothermarchaeaceae*.^3^
*Hydrothermales* (name appeared first in Chuvochina et al. (2019) but the taxon was not proposed)	Genus *Hydrothermus* Chuvochina et al. 2019	Hy.dro.ther.ma'les. N.L. masc. n. *Hydrothermus* type genus of the order; -*ales* ending to denote an order; N.L. fem. pl. n. *Hydrothermales* the *Hydrothermus* order	The order constitutes a monophyletic lineage as described and defined in Parks et al. (2018). The order contains the family *Hydrothermaceae*.^3^
*Kapaibacteriales* (corrig. of ‘*Kapabacteriales*’)	Genus *Kapaibacterium*^4^ corrig. (Kapabacteria, sic) Kantor et al. 2015	Ka.pa.i.bac.te.ri.a'les. N.L. neut. n. *Kapaibacterium* type genus of the order; -*ales* ending to denote an order; N.L. fem. pl. n. *Kapaibacteriales* the *Kapaibacterium* order	The order constitutes a monophyletic lineage as described and defined in Parks et al. (2018). The order contains the family *Kapaibacteriaceae*.^3^
*Johnevansiales* (corrig. of ‘*Evansiales*’)	Genus *Johnevansia^4^ corrig*. Kuechler et al. 2013	John.e.van.si.a'les. N.L. fem. n. *Johnevansia* type genus of the order; -*ales* ending to denote an order; N.L. fem. pl. n. *Johnevansiales* the *Johnevansia* order	The order constitutes a monophyletic lineage as described and defined in Parks et al. (2018). The order contains the family *Johnevansiaceae*.^3^
*Methylomirabilales*	Genus *Methylomirabilis* Ettwig et al. 2010	Me.thy.lo.mi.ra.bi.la'les. N.L. fem. n. *Methylomirabilis* type genus of the order; -*ales* ending to denote an order; N.L. fem. pl. n. *Methylomirabilales* the *Methylomirabilis* order	The order constitutes a monophyletic lineage as described and defined in Parks et al. (2018). The order contains the family *Methylomirabilaceae*^3^
*Muiribacteriales* (corrig. of ‘*Muirbacteriales*’)	Genus *Muiribacterium*^4^ corrig. Barnum et al. 2018	Mui.ri.bac.te.ri.a'les. N.L. neut. n. *Muiribacterium* type genus of the order; -*ales* ending to denote an order; N.L. fem. pl. n. *Muiribacteriales* the *Muiribacterium* order	The order constitutes a monophyletic lineage as described and defined in Parks et al. (2018). The order contains the family *Muiribacteriaceae*.^3^
*Promineifilales* (corrig. of ‘*Promineofilales*’)	Genus *Promineifilum*^4^ corrig. McIlroy et al. 2016	Pro.mi.ne.i.fi.la'les. N.L. neut. n. *Promineifilum* type genus of the order; -*ales* ending to denote an order; N.L. fem. pl. n. *Promineifilales* the *Promineifilum* order	The order constitutes a monophyletic lineage as described and defined in Parks et al. (2018). The order contains the family *Promineifilaceae*.^3^
*Puniceispirillales*	Genus *Puniceispirillum* Oh et al. 2010	Pu.ni.ce.i.spi.ril.la'les. N.L. neut. n. *Puniceispirillum* type genus of the order; -*ales* ending to denote an order; N.L. fem. pl. n. *Puniceispirillales* the *Puniceispirillum* order	The order constitutes a monophyletic lineage as described and defined in Parks et al. (2018). The order contains the family *Puniceispirillaceae*.^3^
*Saccharimonadales*	Genus *Saccharimonas* Albertsen et al. 2013	Sac.cha.ri.mo.na.da'les. N.L. fem. n. *Saccharimonas* type genus of the order; -*ales* ending to denote an order; N.L. fem. pl. n. *Saccharimonadales* the *Saccharimonas* order	The order constitutes a monophyletic lineage as described and defined in Parks et al. (2018). The order contains the family *Saccharimonadaceae*.^3^
*Tenderiales*	Genus *Tenderia* Eddie et al. 2016	Ten.de.ri.a'les. N.L. fem. n. *Tenderia* type genus of the order; -*ales* ending to denote an order; N.L. fem. pl. n. *Tenderiales* the *Tenderia* order	The order constitutes a monophyletic lineage as described and defined in Parks et al. (2018). The order contains the family *Tenderiaceae*.^3^
*Thermobaculales*	Genus *Thermobaculum* Botero et al. 2004	Ther.mo.ba.cu.la'les. N.L. neut. n. *Thermobaculum* type genus of the order; -*ales* ending to denote an order; N.L. fem. pl. n. *Thermobaculales* the *Thermobaculum* order	The order constitutes a monophyletic lineage as described and defined in Parks et al. (2018). The order contains the family *Thermobaculaceae*. ^3^
**Rank of Family (all proposed as fam. nov.)**			
*Azobacteroidaceae*	Genus *Azobacteroides* Hongoh et al. 2008	A.zo.bac.te.ro.i.da.ce'ae. N.L. masc. n. *Azobacteroides* type genus of the family; -*aceae* ending to denote a family; N.L. fem. pl. n. *Azobacteroidaceae* the *Azobacteroides* family	The family constitutes a monophyletic lineage as described and defined in Parks et al. (2018). The family contains the following genera: *Azobacteroides*, and *Candidatus* Symbiothrix.
*Binataceae* (name appeared first in Chuvochina et al. (2019) but the taxon was not proposed)	Genus *Binatus* Chuvochina et al. 2019	Bi.na.ta.ce'ae. N.L. masc. n. *Binatus* type genus of the family; -*aceae* ending to denote a family; N.L. fem. pl. n. *Binataceae* the *Binatus* family	The family constitutes a monophyletic lineage as described and defined in Parks et al. (2018). The family contains the genus *Binatus*.
*Bipolaricaulaceae*	Genus *Bipolaricaulis* Hao et al. 2018	Bi.po.la.ri.cau.la.ce'ae. N.L. masc. n. *Bipolaricaulis* type genus of the family; -*aceae* ending to denote a family; N.L. fem. pl. n. *Bipolaricaulaceae* the *Bipolaricaulis* family	The family constitutes a monophyletic lineage as described and defined in Parks et al. (2018). The family contains the genus *Bipolaricaulis*.
*Cloacimonadaceae*	Genus *Cloacimonas* Pelletier et al. 2008	Clo.a.ci.mo.na.da.ce'ae. N.L. fem. n. *Cloacimonas* type genus of the family; -*aceae* ending to denote a family; N.L. fem. pl. n. *Cloacimonadaceae* the *Cloacimonas* family	The family constitutes a monophyletic lineage as described and defined in Parks et al. (2018). The family contains the following genera: *Cloacimonas, Candidatus* Syntrophosphaera.
*Desulforudaceae*	Genus *Desulforudis* Chivian et al. 2008	De.sul.fo.ru.da.ce'ae N.L. masc. n. *Desulforudis* type genus of the family; -*aceae* ending to denote a family; N.L. fem. pl. n. *Desulforudaceae* the *Desulforudis* family	The family constitutes a monophyletic lineage as described and defined in Parks et al. (2018). The family contains the following genera: Desulforudis, *Candidatus* Desulfopertinax.
*Hadarchaeaceae*	Genus *Hadarchaeum* Chuvochina et al. 2019	Had.ar.chae.a.ce'ae. N.L. neut. n. *Hadarchaeum* type genus of the family; -*aceae* ending to denote a family; N.L. fem. pl. n. *Hadarchaeaceae* the *Hadarchaeum* family	The family constitutes a monophyletic lineage as described and defined in Rinke et al. (2021). The family contains the genus *Hadarchaeum*.
*Hydrothermarchaeaceae*	Genus *Hydrothermarchaeum* Chuvochina et al. 2019	Hy.dro.therm.ar.chae.a.ce'ae. N.L. neut. n. *Hydrothermarchaeum* type genus of the family; -*aceae* ending to denote a family; N.L. fem. pl. n. *Hydrothermarchaeaceae* the *Hydrothermarchaeum* family	The family constitutes a monophyletic lineage as described and defined in Rinke et al. (2021). The family contains the genus *Hydrothermarchaeaum*.
*Hepatobacteraceae*	Genus *Hepatobacter* Ryazanova et al. 2020	He.pa.to.bac.te.ra.ce'ae. N.L. masc. n. *Hepatobacter* type genus of the family; -*aceae* ending to denote a family; N.L. fem. pl. n. *Hepatobacteraceae* the *Hepatobacter* family	The family constitutes a monophyletic lineage as described and defined in Parks et al. (2018). The family contains the genus *Hepatobacter*.
*Hepatoplasmataceae*	Genus *Hepatoplasma* Wang et al. 2004	He.pa.to.plas.ma.ta.ce'ae. N.L. neut. n. *Hepatoplasma* type genus of the family; -*aceae* ending to denote a family; N.L. fem. pl. n. *Hepatoplasmataceae* the *Hepatoplasma* family	The family constitutes a monophyletic lineage as described and defined in Parks et al. (2018). The family contains the genus *Hepatoplasma*.
*Hydrothermaceae* (name appeared first in Chuvochina et al. (2019) but the taxon was not proposed)	Genus *Hydrothermus* Chuvochina et al. 2019	Hyd.ro.ther.ma.ce'ae. N.L. masc. n. *Hydrothermus* type genus of the family; -*aceae* ending to denote a family; N.L. fem. pl. n. *Hydrothermaceae* the *Hydrothermus* family	The family constitutes a monophyletic lineage as described and defined in Parks et al. (2018). The family contains the genus *Hydrothermus*.
*Johnevansiaceae* (corrig. of ‘*Evansiaceae’*)	Genus *Johnevansia*^5^*corrig*. Kuechler et al. 2013	John.e.van.si.a.ce'ae. N.L. fem. n. *Johnevansia* type genus of the family; -*aceae* ending to denote a family; N.L. fem. pl. n. *Johnevansiaceae* the *Johnevansia* family	The family constitutes a monophyletic lineage as described and defined in Parks et al. (2018). The family contains the following genera: *Johnevansia*,^5^*Candidatus* Portiera.
*Kapaibacteriaceae* (corrig. of ‘*Kapabacteriaceae’*)	Genus *Kapaibacterium*^5^ corrig. Kantor et al. 2015	Ka.pa.i.bac.te.ri.a.ce'ae. N.L. neut. n. *Kapaibacterium* type genus of the family; -*aceae* ending to denote a family; N.L. fem. pl. n. *Kapaibacteriaceae* the *Kapaibacterium* family	The family constitutes a monophyletic lineage as described and defined in Parks et al. (2018). The family contains the genus *Kapaibacterium*.^5^
*Magnetobacteriaceae*	Genus *Magnetobacterium* Lin et al. 2014	Mag.ne.to.bac.te.ri.a.ce'ae. N.L. neut. n. *Magnetobacterium* type genus of the family; -*aceae* ending to denote a family; N.L. fem. pl. n. *Magnetobacteriaceae* the *Magnetobacterium* family	The family constitutes a monophyletic lineage as described and defined in Parks et al. (2018). The family contains the genus *Magnetobacterium*.
*Methylomirabilaceae*	Genus *Methylomirabilis* Ettwig et al. 2010	Me.thy.lo.mi.ra.bi.la.ce'ae. N.L. fem. n. *Methylomirabilis* type genus of the family; -*aceae* ending to denote a family; N.L. fem. pl. n. *Methylomirabilaceae* the *Methylomirabilis* family	The family constitutes a monophyletic lineage as described and defined in Parks et al. (2018). The family contains the genus *Methylomirabilis*.
*Muiribacteriaceae* (corrig. of ‘*Muirbacteriaceae*’)	Genus *Muiribacterium*^5^ corrig. Barnum et al. 2018	Mui.ri.bac.te.ri.a.ce'ae. N.L. neut. n. *Muiribacterium* type genus of the family; -*aceae* ending to denote a family; N.L. fem. pl. n. *Muiribacteriaceae* the *Muiribacterium* family	The family constitutes a monophyletic lineage as described and defined in Parks et al. (2018). The family contains the genus *Muiribacterium.^5^*
*Nucleicultricaceae*	Genus *Nucleicultrix* Schulz et al. 2014	Nuc.le.i.cul.tri.ca.ce'ae. N.L. fem. n. *Nucleicultrix* type genus of the family; -*aceae* ending to denote a family; N.L. fem. pl. n. *Nucleicultricaceae* the *Nucleicultrix* family	The family constitutes a monophyletic lineage as described and defined in Parks et al. (2018). The family contains the genus *Nucleicultrix*.
*Obscuribacteraceae*	Genus *Obscuribacter* Soo et al. 2014	Ob.scu.ri.bac.te.ra.ce'ae. N.L. masc. n. *Obscuribacter* type genus of the family; -*aceae* ending to denote a family; N.L. fem. pl. n. *Obscuribacteraceae* the *Obscuribacter* family	The family constitutes a monophyletic lineage as described and defined in Parks et al. (2018). The family contains the genus *Obscuribacter*.
*Promineifilaceae* (corrig. of ‘*Promineofilaceae*’)	Genus *Promineifilum*^5^ corrig. McIlroy et al. 2016	Pro.mi.ne.i.fi.la.ce'ae. N.L. fem. n. *Promineifilum* type genus of the family; -*aceae* ending to denote a family; N.L. fem. pl. n. *Promineifilaceae* the *Promineifilum* family	The family constitutes a monophyletic lineage as described and defined in Parks et al. (2018). The family contains the genus *Promineifilum*.^5^
*Pseudothioglobaceae* (former ‘*Tioglobaceae*’)	Genus *Pseudothioglobus* Van Vliet et al. 2021	Pseu.do.thi.o.glo.ba.ce'ae. N.L. masc. n. *Pseudothioglobus* type genus of the family; -*aceae* ending to denote a family; N.L. fem. pl. n. *Pseudothioglobaceae* the *Pseudothioglobus* family	The family constitutes a monophyletic lineage as described and defined in Parks et al. (2018). The family contains the following genera: *Pseudothioglobus, Candidatus* Thioglobus, *Candidatus* Ruthia, *Candidatus* Thiodubiliella, *Candidatus* Vesicomyosocius.
*Puniceispirillaceae*	Genus *Puniceispirillum* Oh et al. 2010	Pu.ni.ce.i.spi.ril.la.ce'ae. N.L. neut. n. *Puniceispirillum* type genus of the family; -*aceae* ending to denote a family; N.L. fem. pl. n. *Puniceispirillaceae* the *Puniceispirillum* family	The family constitutes a monophyletic lineage as described and defined in Parks et al. (2018). The family contains the genus *Puniceispirillum*.
*Saccharimonadaceae*	Genus *Saccharimonas* Albertsen et al. 2013	Sac.cha.ri.mo.na.da.ce'ae. N.L. fem. n. *Saccharimonas* type genus of the family; -*aceae* ending to denote a family; N.L. fem. pl. n. *Saccharimonadaceae* the *Saccharimonas* family	The family constitutes a monophyletic lineage as described and defined in Parks et al. (2018). The family contains the genus *Saccharimonas*.
*Tenderiaceae*	Genus *Tenderia* Eddie et al. 2016	Ten.de.ri.a.ce'ae. N.L. fem. n. *Tenderia* type genus of the family; -*aceae* ending to denote a family; N.L. fem. pl. n. *Tenderiaceae* the *Tenderia* family	The family constitutes a monophyletic lineage as described and defined in Parks et al. (2018). The family contains the genus *Tenderia*.
*Thermobaculaceae*	Genus *Thermobaculum* Botero et al. 2004	Ther.mo.ba.cu.la.ce'ae. N.L. neut. n. *Thermobaculum* type genus of the order; -*aceae* ending to denote a family; N.L. fem. pl. n. *Thermobaculaceae* the *Thermobaculum* family	The family constitutes a monophyletic lineage as described and defined in Parks et al. (2018). The family contains the genus *Thermobaculum*.

1 Type denotes nomenclature type as defined under the SeqCode.

2 Membership is based on release RS07-R207 of GTDB.

3 Taxon proposed as part of this manuscript.

4 GTDB Latin placeholder name.

5 Name correction as suggested by Oren et al. (2020).

**Table 4.** Description of GTDB-defined new *Candidatus* higher taxa.

Taxon name	Type^1^	E Etymology	Properties and Membership^2^
**Rank of Class (all proposed as class. nov.)**			
*Aerophobia*	Order *Candidatus* Aerophobales^3^	A.e.ro.pho'bi.a. N.L. masc. n. *Aerophobus* a *Candidatus* genus name; -*ia* ending to denote a class; N.L. neut. pl. n. *Aerophobia* the class of the order *Aerophobales*	The class constitutes a monophyletic lineage as described and defined in Parks et al. (2018). The class contains the order *Candidatus* Aerophobales.^3^
*Altiarchaeia* (earlier listed as a placeholder in Rinke et al. 2021))	Order *Candidatus* Altiarchaeales Probst et al. 2014	Al.ti.ar.chae'i.a. N.L. neut. n. *Altiarchaeum* a *Candidatus* genus name; -*ia* ending to denote a class; N.L. neut. pl. n. *Altiarchaeia* the class of the order *Altiarchaeales*	The class constitutes a monophyletic lineage as described and defined in Parks et al. (2018). The class contains the order *Candidatus* Altiarchaeales.
*Aminicenantia*	Order *Candidatus* Aminicenantales^3^	A.mi.ni.ce.nan'ti.a. N.L. masc. n. *Aminicenans* a *Candidatus* genus name; -*ia* ending to denote a class; N.L. neut. pl. n. *Aminicenantales* the class of the order *Aminicenantales*	The class constitutes a monophyletic lineage as described and defined in Parks et al. (2018). The class contains the order *Candidatus* Aminicenantales.^3^
*Calescibacteriia*	Order *Candidatus* Calescibacteriale^3^	Ca.les.ci.bac.te.ri'i.a. N.L. neut. n. *Calescibacterium* a *Candidatus* genus name; -*ia* ending to denote a class; N.L. neut. pl. n. *Calescibacteriia* the class of the order *Calescibacteriales*	The class constitutes a monophyletic lineage as described and defined in Parks et al. (2018). The class contains the order *Candidatus* Calescibacteriales.^3^
*Entotheonellia*	Order *Candidatus* Entotheonellales^3^	En.to.the.o.nel'li.a. N.L. fem. n. *Entotheonella* a *Candidatus* genus name; -*ia* ending to denote a class; N.L. neut. pl. n. *Entotheonellia* the class of the order *Entotheonellales*	The class constitutes a monophyletic lineage as described and defined in Parks et al. (2018). The class contains the order *Candidatus* Entotheonellales^3^
*Hydrogenedentia*	Order *Candidatus* Hydrogenedentaes^3^	Hy.dro.gen.e.den'ti.a. N.L. masc. n. *Hydrogenedens* a *Candidatus* genus name; -*ia* ending to denote a class; N.L. neut. pl. n. *Hydrogenedentia* the class of the order *Hydrogenedentales*	The class constitutes a monophyletic lineage as described and defined in Parks et al. (2018). The class contains the order *Candidatus* Hydrogenedentales.^3^
*Latescibacteria* (not *Candidatus* Latescibacteria Rinke et al. 2013)	Order *Candidatus* Latescibacterales^3^	La.te.sci.bac.te'ri.a. N.L. masc. n. *Latescibacter* a *Candidatus* genus name; -*ia* ending to denote a class; N.L. neut. pl. n. *Latescibacteria* the class of the order *Latescibacterales*	The class constitutes a monophyletic lineage as described and defined in Parks et al. (2018). The class contains the order *Candidatus* Latescibacterales.^3^
*Paceibacteria*	Order *Candidatus* Paceibacterales^3^	Pa.ce.i.bac.te'ria. N.L. masc. n. *Paceibacter* a *Candidatus* genus name; -*ia* ending to denote a class; N.L. neut. pl. n. *Paceibacteria* the class of the order *Paceibacterales*	The class constitutes a monophyletic lineage as described and defined in Parks et al. (2018). The class contains the following orders: *Candidatus* Paceibacterales,^3^*Candidatus* Moranbacterales,^4^*Candidatus* Portnoybacterales,^4^*Candidatus* Ryanbacterales,^4^*Candidatus* Spechtbacterales,^4^*Candidatus* Sungbacterales,^4^*Candidatus* Terrybacterales.^4^
**Rank of Order (all proposed as ord. nov.)**			
*Aerophobales*	Genus *Candidatus* Aerophobus Rinke et al. 2013	A.e.ro.pho.ba'les. N.L. masc. n. *Aerophobus* a *Candidatus* genus name; -*ales* ending to denote an order; N.L. fem. pl. n. *Aerophobales* the *Aerophobus* order	The order constitutes a monophyletic lineage as described and defined in Parks et al. (2018). The order contains the family *Candidatus* Aerophobaceae.^3^
*Aminicenantales*	Genus *Candidatus* Aminicenans Rinke et al. 2013	A.mi.ni.ce.nan.ta'les. N.L. masc. n. *Aminicenans* a *Candidatus* genus name; -*ales* ending to denote an order; N.L. fem. pl. n. *Aminicenantales* the *Aminicenans* order	The order constitutes a monophyletic lineage as described and defined in Parks et al. (2018). The order contains the following families: *Candidatus* Aminicenantaceae,^3^*Candidatus* Saccharicenantaceae.
*Berkiellales*	Genus *Candidatus* Berkiella Mehari et al. 2016	Ber.ki.el.la'les. N.L. fem. n. *Berkiella* a *Candidatus* genus name; -*ales* ending to denote an order; N.L. fem. pl. n. *Berkiellales* the *Berkiella* order	The order constitutes a monophyletic lineage as described and defined in Parks et al. (2018). The order contains the family *Candidatus* Berkiellaceae.^3^
*Calescibacteriales*	Genus *Candidatus* Calescibacterium Rinke et al. 2013	Ca.les.ci.bac.te.ri.a'les. N.L. neut. n. *Calescibacterium* a *Candidatus* genus name; -*ales* ending to denote an order; N.L. fem. pl. n. *Calescibacteriales* the *Calescibacterium* order	The order constitutes a monophyletic lineage as described and defined in Parks et al. (2018). The order contains the family *Candidatus* Calescibacteriaceae.^3^
*Competibacterales*	Genus *Candidatus* Competibacter Crocetti et al. 2002	Com.pe.ti.bac.te.ra'les. N.L. masc. n. *Competibacter* a *Candidatus* genus name; -*ales* ending to denote an order; N.L. fem. pl. n. *Competibacterales* the *Competibacter* order	The order constitutes a monophyletic lineage as described and defined in Parks et al. (2018). The order contains the family *Candidatus* Competibacteraceae.
*Entotheonellales*	Genus *Candidatus* Entotheonella Schmidt et al. 2000	En.to.the.o.nel.la'les. N.L. fem. n. *Entotheonella* a *Candidatus* genus name; -*ales* ending to denote an order; N.L. fem. pl. n. *Entotheonellales* the *Entotheonella* order	The order constitutes a monophyletic lineage as described and defined in Parks et al. (2018). The order contains the family *Candidatus* Entotheonellaceae.^3^
*Hydrogenedentales* (corrig. of ‘*Hydrogenedentiales*’)	Genus *Candidatus* Hydrogenedens Rinke et al. 2013	Hy.dro.gen.e.den.ta'les. N.L. masc. n. *Hydrogenedens* a *Candidatus* genus name; -*ales* ending to denote an order; N.L. fem. pl. n. *Hydrogenedentales* the *Hydrogenedens* order	The order constitutes a monophyletic lineage as described and defined in Parks et al. (2018). The order contains the family *Candidatus* Hydrogenedentaceae.^3^
*Latescibacterales*	Genus *Candidatus* Latescibacter Rinke et al. 2013	La.tes.ci.bac.te.ra'les. N.L. masc. n. *Latescibacter* a *Candidatus* genus name; -*ales* ending to denote an order; N.L. fem. pl. n. *Latescibacterales* the *Latescibacter* order	The order constitutes a monophyletic lineage as described and defined in Parks et al. (2018). The order contains the family *Candidatus* Latescibacteraceae.^3^
*Paceibacterales*	Genus *Candidatus* Paceibacter Rinke et al. 2013	Pa.ce.i.bac.te.ra'les. N.L. masc. n. *Paceibacter* a *Candidatus* genus name; -*ales* ending to denote an order; N.L. fem. pl. n. *Paceibacterales* the *Paceibacter* order	The order constitutes a monophyletic lineage as described and defined in Parks et al. (2018). The order contains the following families: *Candidatus* Paceibacteraceae,^3^*Candidatus* Staskawiczbacteraceae.^4^
*Paracaedibacterales*	Genus *Candidatus* Paracaedibacter Horn et al. 1999	Pa.ra.cae.di.bac.te.ra'les. N.L. masc. n. *Paracaedibacter* a *Candidatus* genus name; -*ales* ending to denote an order; N.L. fem. pl. n. *Paracaedibacterales* the *Paracaedibacter* order	The order constitutes a monophyletic lineage as described and defined in Parks et al. (2018). The order contains the family *Candidatus* Paracaedibacteraceae.
*Poriferisulfidales* (corrig. of ‘*Porisulfidales*’)	Genus *Candidatus* Poriferisulfidus^5^ corrig. Lavy et al. 2018	Po.ri.fe.ri.sul.fi.da'les. N.L. masc. n. *Poriferisulfidus* a *Candidatus* genus name; -*ales* ending to denote an order; N.L. fem. pl. n. *Poriferisulfidales* the *Poriferisulfidus* order	The order constitutes a monophyletic lineage as described and defined in Parks et al. (2018). The order contains the family *Candidatus* Poriferisulfidaceae.^3^
**Rank of Family (all proposed as fam. nov.)**			
*Aerophobaceae*	Genus *Candidatus* Aerophobus (Rinke et al. 2013)	A.e.ro.pho.ba.ce'ae. N.L. masc. n. *Aerophobus* a *Candidatus* genus name; -*aceae* ending to denote a family; N.L. fem. pl. n. *Aerophobaceae* the *Aerophobus* family	The family constitutes a monophyletic lineage as described and defined in Parks et al. (2018). The family contains the genus *Candidatus* Aerophobus.
*Aminicenantaceae*	Genus *Candidatus* Aminicenans (Rinke et al. 2013)	A.mi.ni.ce.nan.ta.ce'ae. N.L. masc. n. *Aminicenans* a *Candidatus* genus name; -*aceae* ending to denote a family; N.L. fem. pl. n. *Aminicenantaceae* the *Aminicenans* family	The family constitutes a monophyletic lineage as described and defined in Parks et al. (2018). The family contains the genus *Candidatus* Aminicenans.
*Arcanibacteraceae* (corrig. of ‘*Arcanobacteraceae*’)	Genus *Candidatus* Arcanibacter^5^ corrig. Martijn et al. 2015	Ar.ca.ni.bac.te.ra.ce'ae. N.L. masc. n. *Arcanibacter* a *Candidatus* genus name; -*aceae* ending to denote a family; N.L. fem. pl. n. *Arcanibacteraceae* the *Arcanibacter* family	The family constitutes a monophyletic lineage as described and defined in Parks et al. (2018). The family contains the genus *Candidatus* Arcanibacter.^5^
*Berkiellaceae*	Genus *Candidatus* Berkiella Mehari et al. 2016	Ber.ki.el.la.ce'ae. N.L. fem. n. *Berkiella* a *Candidatus* genus name; -*aceae* ending to denote a family; N.L. fem. pl. n. *Berkiellaceae* the *Berkiella* family	The family constitutes a monophyletic lineage as described and defined in Parks et al. (2018). The family contains the genus *Candidatus* Berkiella.
*Caenarcanaceae*	Genus *Candidatus* Caenarcanum Soo et al. 2014	Caen.ar.ca.na.ce'ae. N.L. neut. n. *Caenarcanum* a *Candidatus* genus name; -*aceae* ending to denote a family; N.L. fem. pl. n. *Caenarcanaceae* the *Caenarcanum* family	The family constitutes a monophyletic lineage as described and defined in Parks et al. (2018). The family contains the genus *Candidatus* Caenarcanum.
*Calescibacteriaceae*	Genus *Candidatus* Calescibacterium Rinke et al. 2013	Ca.les.ci.bac.te.ri.a.ce'ae. N.L. neut. n. *Calescibacterium* a *Candidatus* genus name; -*aceae* ending to denote a family; N.L. fem. pl. n. *Calescibacteriaceae* the *Calescibacterium* family	The family constitutes a monophyletic lineage as described and defined in Parks et al. (2018). The family contains the genus *Candidatus* Calescibacterium.
*Entotheonellaceae*	Genus *Candidatus* Entotheonella Schmidt et al. 2000	En.to.the.o.nel.la.ce'ae. N.L. fem. n. *Entotheonella* a *Candidatus* genus name; -*aceae* ending to denote a family; N.L. fem. pl. n. *Entotheonellaceae* the *Entotheonella* family	The family constitutes a monophyletic lineage as described and defined in Parks et al. (2018). The family contains the genus *Candidatus* Entotheonella.
*Gastranaerophilaceae*	Genus *Candidatus* Gastranaerophils Soo et al. 2014	Gastr.an.a.e.ro.phi.la.ce'ae. N.L. masc. n. *Gastranaerophilus* a *Candidatus* genus name; -*aceae* ending to denote a family; N.L. fem. pl. n. *Gastranaerophilaceae* the *Gastranaerophilus* family	The family constitutes a monophyletic lineage as described and defined in Parks et al. (2018). The family contains the genus *Candidatus* Gastranaerophilus.
*Hydrogenedentaceae*	Genus *Candidatus* Hydrogenedens Rinke et al. 2013	Hy.dro.gen.e.den.ta.ce'ae. N.L. masc. n. *Hydrogenedens* a *Candidatus* genus name; -*aceae* ending to denote a family; N.L. fem. pl. n. *Hydrogenedentaceae* the *Hydrogenedens* family	The family constitutes a monophyletic lineage as described and defined in Parks et al. (2018). The family contains the genus *Candidatus* Hydrogenedens.
*Korobacteraceae* (corrig. of ‘*Koribacteraceae*’)	Genus *Candidatus* Korobacter^5^ corrig. Ward et al. 2009	Ko.ro.bac.te.ra.ce'ae. N.L. masc. n. *Korobacter* a *Candidatus* genus name; -*aceae* ending to denote a family; N.L. fem. pl. n. *Korobacteraceae* the *Korobacter* family	The family constitutes a monophyletic lineage as described and defined in Parks et al. (2018). The family contains the genus *Candidatus* Korobacter and *Candidatus* Sulfotelmatobacter.
*Latescibacteraceae*	Genus *Candidatus* Latescibacter Rinke et al. 2013	La.tes.ci.bac.te.ra.ce'ae. N.L. masc. n. *Latescibacter* a *Candidatus* genus name; -*aceae* ending to denote a family; N.L. fem. pl. n. *Latescibacteraceae* the *Latescibacter* family	The family constitutes a monophyletic lineage as described and defined in Parks et al. (2018). The family contains the genus *Candidatus* Latescibacter.
*Paceibacteraceae*	Genus *Candidatus* Paceibacter Rinke et al. 2013	Pa.ce.i.bac.te.ra.ce'ae. N.L. masc. n. *Paceibacter* a *Candidatus* genus name; -*aceae* ending to denote a family; N.L. fem. pl. n. *Paceibacteraceae* the *Paceibacter* family	The family constitutes a monophyletic lineage as described and defined in Parks et al. (2018). The family contains the genus *Candidatus* Paceibacter.
*Poriferisulfidaceae* (corrig. of ‘*Porisulfidaceae*’)	Genus *Candidatus* Poriferisulfidus^5^ corrig. Lavy et al. 2018	Po.ri.fe.ri.sul.fi.da.ce'ae. N.L. masc. n. *Poriferisulfidus* a *Candidatus* genus name; -*aceae* ending to denote a family; N.L. fem. pl. n. *Poriferisulfidaceae* the *Poriferisulfidus* family	The family constitutes a monophyletic lineage as described and defined in Parks et al. (2018). The family contains the genus *Candidatus* Poriferisulfidus.^5^

1 Type denotes provisional *Candidatus* nomenclature type according to the type defined under the ICNP.

2 Membership is based on release RS07-R207 of GTDB.

3 Taxon proposed as part of this manuscript.

4 GTDB Latin placeholder name.

5 Name correction as suggested by Oren et al. (2020).

The majority of new higher taxon names proposed in this study (excluding *Candidatus* taxa) are at the rank of family (54%) with only five new phylum names required (2%), reflecting the majority of GTDB reclassifications occurring at lower ranks (Parks et al. 2018). Collectively, these taxa belong to 26 of the 166 prokaryotic phyla recognized in GTDB release R07-RS207 with the majority belonging to the bacterial phyla *Bacillota/*‘*Firmicutes*’ (45%) and *Pseudomonadota/*‘*Proteobacteria*’ (23%). Names validly published under the International Code of Nomenclature for algae, fungi and plants (ICN) are now recognized by the ICNP (Oren et al. 2021a) and, therefore, we propose 10 new cyanobacterial higher taxa based on genera originally validly published under the ICN (Table 1). Another recent revision of the ICNP is the inclusion of the rank of phylum under its rules (Oren et al. 2021b), allowing us to propose four phyla based on genera with validly published names under the ICNP (Table 1). A number of existing phylum names occurring in an effective publication have already been orthographically corrected in GTDB (Table S2, Supporting Information), making them compliant with the recent emendation of the code (Oren et al. 2021b).

Finally, 44 bacterial and 20 archaeal (previously published in Rinke et al. 2021) GTDB higher taxon names were left as provisional either because (i) they lack type genera and species, or (ii) the original authors of the type species are expected to independently propose the high rank name(s) associated with that species (Table S3, Supporting Information). These include taxa belonging to the Patescibacteria (CPR) that were originally proposed as *Candidatus* phyla and whose names we subsequently modified to ranks below phylum in early GTDB releases. In order to validate names of such higher taxa, a type species with a corresponding genus stem will need to be designated and deposited according to the criteria of one of the nomenclature codes. Alternatively, these names can be replaced with new names based on newly designated types with validly published names.

In conclusion, the proposal of 329 higher taxon names will ensure that future releases of GTDB will provide a stable taxonomy to the research community largely in alignment with nomenclatural codes. It also illustrates how the ICNP and SeqCode can be used in a complementary fashion.

## Supplementary Material

fnad071_Supplemental_Files
