# Supplementary material for: Proposal of names for 329 higher rank taxa defined in the Genome Taxonomy Database under two prokaryotic codes
Source: FEMS Microbiol Lett. 2023 Jul 21;370:fnad071. doi: 10.1093/femsle/fnad071 (PMC10408702; doi:10.1093/femsle/fnad071)
Supplement: fnad071_Supplemental_Files [file fnad071_supplemental_files.zip › Table_S1_S2_S3_rev_1.docx]

**Supplementary Table 1.** Genome sequences proposed as types for *Candidatus* species that satisfy the SeqCode data quality recommendations.

| **Proposed species name** | **NCBI Identifier** | **Genome Type** | **Completeness** | **Contamination** | **Checkm2_compl** | **Checkm2_cont** | **Contig count** | **MIMAG_high** | **MIMAG_med** | **N50_contig_kb** | **Longest_contig_kb** | **tRNA_count** | **tRNA_aa_count** | **ssu_count** | **ssu_length** | **lsu_23s_count** | **lsu_23s_length** |
| --- | --- | --- | --- | --- | --- | --- | --- | --- | --- | --- | --- | --- | --- | --- | --- | --- | --- |
| *Cloacimonas acidaminivorans^1^* | GCA_000146065.1 | Isolate | 100 | 1.1 | 99.91 | 0.19 | 1 | TRUE | FALSE | 2246.82 | 2246.82 | 46 | 20 | 2 | 1598 | 2 | 3037 |
| *Puniceispirillum marinum* | GCA_000024465.1 | Isolate | 99.94 | 0.15 | 100 | 0.09 | 1 | TRUE | FALSE | 2753.527 | 2753.527 | 36 | 20 | 1 | 1484 | 1 | 2715 |
| *Tenderia electrophaga* | GCA_001447805.1 | derived from environmental sample | 99.63 | 2.05 | 100 | 0.53 | 2 | TRUE | FALSE | 3656.958 | 3656.958 | 41 | 20 | 1 | 1499 | 1 | 2890 |
| *Pseudothioglobus singularis* | GCA_001281385.1 | Isolate | 98.68 | 0 | 99.99 | 0.23 | 1 | TRUE | FALSE | 1714.148 | 1714.148 | 38 | 20 | 1 | 1536 | 1 | 2885 |
| *Desulforudis audaxviator* | GCA_000018425.1 | Isolate | 98.09 | 0.32 | 99.98 | 0.18 | 1 | TRUE | FALSE | 2349.476 | 2349.476 | 45 | 20 | 2 | 1697 | 3 | 3372 |
| *Obscuribacter phosphatis* | GCA_001899315.1 | derived from metagenome | 97.44 | 2.28 | 95.27 | 4.37 | 55 | TRUE | FALSE | 246.807 | 735.22 | 42 | 20 | 1 | 1488 | 1 | 2957 |
| *Kapaibacterium thiocyanatum^1^* | GCA_001899175.1 | derived from metagenome | 96.99 | 0 | 99.28 | 0.05 | 31 | TRUE | FALSE | 373.065 | 951.664 | 42 | 20 | 1 | 1483 | 1 | 2789 |
| *Muiribacterium halophilum^1^* | GCA_002869225.1 | derived from metagenome | 96.56 | 3.5 | 90.31 | 4.31 | 154 | TRUE | FALSE | 32.07 | 182.03 | 39 | 20 | 1 | 1556 | 1 | 3009 |
| *Methylomirabilis oxygeniifera^1^* | GCA_000091165.1 | derived from environmental sample | 96.26 | 2.59 | 100 | 0.45 | 1 | TRUE | FALSE | 2752.854 | 2752.854 | 47 | 20 | 1 | 1558 | 1 | 3023 |
| *Promineifilum breve^1^* | GCA_900066015.1 | Isolate | 93.64 | 3.64 | 99.93 | 1.58 | 3 | TRUE | FALSE | 3989.163 | 3989.163 | 48 | 20 | 1 | 1504 | 1 | 2927 |
| *Magnetobacterium casense^1^* | GCA_000714715.1 | Isolate | 92.42 | 0.91 | 92.54 | 0.23 | 70 | TRUE | FALSE | 90.253 | 252.526 | 41 | 20 | 1 | 1525 | 1 | 2981 |
| *Hepatobacter penaei* | GCA_000742475.1 | Isolate | 91.94 | 0 | 97.11 | 0 | 15 | TRUE | FALSE | 133.248 | 241.151 | 44 | 20 | 1 | 1612 | 1 | 2746 |
| *Hydrothermus pacificus^2^* | GCA_002011615.1 | derived from metagenome | 91.53 | 0 | 95.11 | 0.02 | 19 | TRUE | FALSE | 176.033 | 342.452 | 45 | 20 | 1 | 1563 | 1 | 3900 |
| *Bipolaricaulis anaerobius* | GCA_900465355.1 | Isolate | 91.53 | 0 | 98.02 | 0.04 | 1 | TRUE | FALSE | 1324.338 | 1324.338 | 46 | 20 | 1 | 1562 | 1 | 3023 |
| *Binatus soli^2^* | GCA_002479255.1 | derived from metagenome | 97.48 | 0.84 | 98.02 | 0.01 | 65 | FALSE | TRUE | 138.054 | 233.241 | 48 | 20 | 1 | 1563 | 2 | 1888 |
| *Hepatoplasma crinochetorum* | GCA_000582535.1 | Isolate | 92.17 | 0 | 97.05 | 0.05 | 1 | FALSE | TRUE | 657.101 | 657.101 | 27 | 20 | 1 | 1533 | 1 | 2933 |
| *Azobacteroides pseudotrichonymphae* | GCA_000010645.1 | derived from single cell | 89.51 | 1.09 | 99.95 | 0 | 5 | FALSE | TRUE | 1114.206 | 1114.206 | 38 | 20 | 2 | 1516 | 1 | 2887 |
| *Nucleicultrix amoebiphila* | GCA_002117145.1 | Isolate | 78.38 | 0 | 96.55 | 0.2 | 1 | FALSE | TRUE | 1838.212 | 1838.212 | 44 | 20 | 2 | 1483 | 2 | 2748 |
| *Saccharimonas aalborgensis* | GCA_000392435.1 | Isolate | 67.13 | 0.93 | 99.75 | 0.33 | 1 | FALSE | TRUE | 1013.781 | 1013.781 | 46 | 20 | 1 | 1500 | 1 | 3190 |
| *Johnevansia muelleri^1^* | GCA_000953435.1 | Isolate | 27.08 | 0 | 96.63 | 0.13 | 1 | FALSE | FALSE | 357.498 | 357.498 | 32 | 20 | 1 | 1547 | 1 | 2960 |
| *Thermobaculum terrenum* | GCA_000025005.1 | Isolate | 98.58 | 0 | 99.97 | 0.8 | 2 | FALSE | TRUE | 2026947 | 2026947 | 51 | 20 | 2 | 1502 | 2 | 2927 |
| *Hadarchaeum yellowstonense^2^* | GCA_001515205.2 | derived from metagenome | 89.72 | 1.87 | 95.13 | 1.22 | 64 | TRUE | FALSE | 23.895 | 74.826 | 32 | 20 | 2 | 1205 | 2 | 2599 |
| Hydrothermarchaeum profundi^2^ | GCA_002011125.1 | derived from metagenome | 98.13 | 1.87 | 97.98 | 1.41 | 22 | TRUE | FALSE | 149.032 | 364.381 | 47 | 19 | 1 | 1475 | 1 | 3000 |

^1^ Name corrected as suggested by Oren *et al.* (2020)

^2^ Name replicated from Chuvochina et al. (2019) to support the proposal of higher taxa names.

**Supplementary Table 2.** Corrections implemented in GTDB for effectively published names of phyla defined elsewhere.

| **Taxon name** | **Type^1^** | **Etymology** | **Properties and Membership^2^** |
| --- | --- | --- | --- |
| *Candidatus* Aenigmatarchaeota corrig.  (effectively published synonym: *Candidatus* Aenigmarchaeota Rinke *et al*., 2013) | Genus *Candidatus* Aenigmatarchaeum^4^ corrig. Rinke *et al*. 2013 | Ae.nig.mat.ar.chaeo’ta. N.L. neut. n. *Aenigmatarchaeum* a Candidatus genus name; -*ota* ending to denote a phylum; N.L. neut. pl. n. *Aenigmatarchaeota* the *Aenigmatarchaeum* phylum | The properties of the phylum are as given by Rinke *et al*., 2013. Membership: *Candidatus* Aenigmarchaeia (Rinke *et al*., 2021). |
| *Candidatus* Aerophobota corrig.  (effectively published synonym: *Candidatus* Aerophobetes Rinke *et al.* 2013) | Genus *Candidatus* Aerophobus Rinke *et al.* 2013 | A.e.ro.pho.bo’ta. N.L. masc. n. *Aerophobus* a Candidatus genus name; -*ota* ending to denote a phylum; N.L. pl. neut. n. *Aerophobota* the *Aerophobus* phylum | The properties of the phylum are as given by Rinke *et al.,* 2013. Membership: *Candidatus* Aerophobia^3^ |
| *Candidatus* Calescibacteriota corrig.  (effectively published synonym: *Candidatus* Calescamantes Rinke *et al*., 2013) | Genus *Candidatus* Calescibacterium Rinke *et al*., 2013 | Ca.les.ci.bac.te.ri.o’ta. N.L. neut. n. *Calescibacterium* a Candidatus genus name; -*ota* ending to denote a phylum; N.L. pl. neut. n. *Calescibacteriota* the *Calescibacterium* phylum | The properties of the phylum are as given by Rinke *et al*., 2013. Membership: *Candidatus* Calescibacteriia^3^. |
| *Candidatus* Cloacimonadota corrig.  (effectively published synonym: *Candidatus* Cloacimonetes Rinke *et al*., 2013) | Genus *Candidatus* Cloacimonas Pelletier *et al.* 2008 | Clo.a.ci.mo.na.do’ta. N.L. fem. n. *Cloacimonas* a Candidatus genus name; -*ota* ending to denote a phylum; N.L. pl. neut. n. *Cloacimonadota* the *Cloacimonas* phylum | The properties of the phylum are as given by Rinke *et al*., 2013. Membership: *Candidatus* Cloacimonadia^3^ |
| *Candidatus* Dormibacterota corrig.  (effectively published synonym: *Candidatus* Dormibacteraeota Ji *et al.* 2017) | Genus *Candidatus* Dormibacter Ji *et al.* 2017 | Dor.mi.bac.te.ro’ta. N.L. masc. n. *Dormibacter* a Candidatus genus name; -*ota* ending to denote a phylum; N.L. pl. neut. n. *Dormibacterota* the *Dormibacter* phylum | The properties of the phylum are as given by Ji *et al.,* 2017. Membership: *Candidatus* Dormibacteria (Montgomery *et al.*, 2021) |
| *Candidatus* Eremiobacterota  corrig.  (effectively published synonym: *Candidatus* Eremiobacteraeota Ji *et al.* 2017) | Genus *Candidatus* Eremiobacter Ji *et al.* 2017 | E.re.mi.o.bac.te.ro’ta. N.L. masc. n. *Eremiobacter* a Candidatus genus name; -*ota* ending to denote a phylum; N.L. pl. neut. n. *Eremiobacterota* the *Eremiobacter* phylum | The properties of the phylum are as given by Ji *et al.,* 2017. Membership: *Candidatus* Eremiobacteria (Ward *et al.* 2019), *Candidatus* Xenobia (Ji *et al*., 2021) |
| *Candidatus* Fermentibacterota corrig.  (effectively published synonym: *Candidatus* Fermentibacteria Kirkegaard *et al.* 2016) | Genus *Candidatus* Fermentibacter Kirkegaard *et al.* 2016 | Fer.men.ti.bac.te.ro’ta. N.L. masc. n. *Fermentibacter* a Candidatus genus name; -*ota* ending to denote a phylum; N.L. pl. neut. n. *Fermentibacterota* the *Fermentibacter* phylum | The properties of the phylum are as given by Kirkegaard *et al.,* 2016. Membership: *Candidatus* Fermentibacteria (Kirkegaard *et al.,* 2016) |
| *Candidatus* Hydrogenedentota corrig.  (effectively published synonym: *Candidatus* Hydrogenedentes Rinke et al., 2013) | Genus *Candidatus* Hydrogenedens Rinke et al., 2013 | Hyd.ro.ge.ne.den.to’ta. N.L. masc. n. *Hydrogenedens* a Candidatus genus name; -*ota* ending to denote a phylum; N.L. pl. neut. n. *Hydrogenedentota* the *Hydrogenedens* phylum | The properties of the phylum are as given by Rinke *et al*., 2013. Membership: *Candidatus* Hydrogenedentia^3^ |
| *Candidatus* Latescibacterota corrig.  (effectively published synonym: *Candidatus* Latescibacteria Rinke *et al*., 2013) | Genus *Candidatus* Latescibacter Rinke *et al*., 2013 | La.te.sci.bac.te.ro’ta. N.L. masc. n. *Latescibacter* a Candidatus genus name; -*ota* ending to denote a phylum; N.L. pl. neut. n. *Latescibacterota* the *Latescibacter* phylum | The properties of the phylum are as given by Rinke *et al*., 2013. Membership: *Candidatus* Latescibacteria^3^ |
| *Candidatus* Moduliflexota corrig.  (effectively published synonym: *Candidatus* Modulibacteria Sekiguchi *et al.,* 2015) | Genus *Candidatus* Moduliflexus Sekiguchi *et al.,* 2015 | Mo.du.li.fle.xo’ta. N.L. masc. n. *Moduliflexus* a Candidatus genus name; -*ota* ending to denote a phylum; N.L. pl. neut. n. *Moduliflexota* the *Moduliflexus* phylum | The properties of the phylum are as given by Sekiguchi *et al.,* 2015. Membership: *Candidatus* Moduliflexia (Sekiguchi *et al.,* 2015) |

^1^ Type denotes nomenclature type as defined under ICNP

^2^ Membership is based on release R06-RS207 of GTDB

^3^ Taxon proposed as part of this ms

^4^ GTDB Latin placeholder name

^5^ Name correction as suggested by Oren *et al.* (2020)

**Supplementary Table 3.** GTDB-defined higher taxa that retain their provisional names and reasons for their exclusion from the descriptions.

| **Taxon name** | **Rank** | **Original name and rank** | **Reference** | **Reason** |
| --- | --- | --- | --- | --- |
| *Cyanobacteriia* | class. nov. | N/A | This paper. | Alternative class name exists under the ICNP and validation under the SeqCode will not give priority to this name. |
| *Thorarchaeia* | class. nov. | N/A | This paper. | No type material and no designated nomenclature type. |
| *Dehalobacteriia* | class. nov. | N/A | This paper. | Proposed type is the effectively published genus ‘Dehalobacterium’ or order ‘Dehalobacteriales’; can be potentially proposed under ICNP. |
| *Nezhaarchaeales* | ord. nov. | *Candidatus* Nezhaarchaeota (phylum) | Wang *et al*., 2019 | No type material and no designated nomenclature type. |
| *Geothermarchaeales* | ord. nov. | *Candidatus* Geothermarchaeota (phylum) | Jungbluth *et al*., 2017 | No type material and no designated nomenclature type. |
| *Buchananbacterales* | ord. nov. | *Candidatus* Buchananbacteria (phylum) | Anantharaman *et al.* 2016 | No type material and no designated nomenclature type. |
| *Magasanikbacterales* | ord. nov. | *Candidatus* Magasanikbacteria (phylum) | Brown *et al.* 2015 | No type material and no designated nomenclature type. |
| *Veblenbacterales* | ord. nov. | *Candidatus* Veblenbacteria (phylum) | Anantharaman *et al.* 2016 | No type material and no designated nomenclature type. |
| *Absconditabacterales* | ord. nov. | *Candidatus* Veblenbacteria (phylum) | Hug *et al.* 2016 | No type material and no designated nomenclature type. |
| *Curtissbacterales* | ord. nov. | *Candidatus* Curtissbacteria (phylum) | Brown *et al.* 2015 | No type material and no designated nomenclature type. |
| *Daviesbacterales* | ord. nov. | *Candidatus* Daviesbacteria (phylum) | Brown *et al.* 2015 | No type material and no designated nomenclature type. |
| *Levybacterales* | ord. nov. | *Candidatus* Levybacteria (phylum) | Brown *et al.* 2015 | No type material and no designated nomenclature type. |
| *Woykebacterales* | ord. nov. | *Candidatus* Woykebacteria (phylum) | Anantharaman *et al.* 2016 | No type material and no designated nomenclature type. |
| *Portnoybacterales* | ord. nov. | *Candidatus* Portnoybacteria (phylum) | Anantharaman *et al.* 2016 | No type material and no designated nomenclature type. |
| *Ryanbacterales* | ord. nov. | *Candidatus* Ryanbacteria (phylum) | Anantharaman *et al.* 2016 | No type material and no designated nomenclature type. |
| *Spechtbacterales* | ord. nov. | *Candidatus* Spechtbacteria (phylum) | Anantharaman *et al.* 2016 | No type material and no designated nomenclature type. |
| *Sungbacterales* | ord. nov. | *Candidatus*  Sungbacteria (phylum) | Anantharaman *et al.* 2016 | No type material and no designated nomenclature type. |
| *Terrybacterales* | ord. nov. | *Candidatus*  Terrybacteria (phylum) | Anantharaman *et al.* 2016 | No type material and no designated nomenclature type. |
| *Moranbacterales* | ord. nov. | *Candidatus*  Moranbacteria (phylum) | Brown *et al.* 2015 | No type material and no designated nomenclature type. |
| *Rokubacteriales* | ord. nov. | *Candidatus*  Rokubacteria (phylum) | Hug *et al.* 2016 | No type material and no designated nomenclature type. |
| *Dehalobacteriales* | ord. nov. | N/A | This paper. | Proposed type is the effectively published genus ‘Dehalobacterium’; can be potentially proposed under ICNP |
| *Thermosynechococcales* | ord. nov. | N/A | This paper. | Proposed type is the effectively published genus ‘Thermosynechococcus’; unresolved nomenclature type |
| *Rubeoparvulales* | ord. nov. | N/A | This paper. | Proposed type is the effectively published genus ‘Rubeoparvulum’; Untrusted source |
| *Odinarchaeales* | ord. nov. | N/A | This paper. | No type material and no designated nomenclature type. |
| *Helarchaeales* | ord. nov. | N/A | This paper. | No type material and no designated nomenclature type. |
| *Pedosphaerales* | ord. nov. | N/A | This paper. | Proposed type is the effectively published genus ‘Pedosphaera’; does not satisfy SeqCode quality criteria |
| *Pedosphaeraceae* | fam. nov. | N/A | This paper. | Proposed type is the effectively published genus ‘Pedosphaera’; does not satisfy SeqCode quality criteria |
| *Rubeoparvulaceae* | fam. nov. | N/A | This paper. | Proposed type is the effectively published genus ‘Rubeoparvulum’; Untrusted source |
| *Amesbacteraceae* | fam. nov. | *Candidatus* Amesbacteria (phylum) | Brown *et al.* 2015 | No type material and no designated nomenclature type. |
| *Chisholmbacteraceae* | fam. nov. | *Candidatus* Chisholmbacteria (phylum) | Anantharaman *et al.* 2016 | No type material and no designated nomenclature type. |
| *Staskawiczbacteraceae* | fam. nov. | *Candidatus* Staskawiczbacteria (phylum) | Anantharaman *et al.* 2016 | No type material and no designated nomenclature type. |
| *Brennerbacteraceae* | fam. nov. | *Candidatus* Brennerbacteria (phylum) | Anantharaman *et al.* 2016 | No type material and no designated nomenclature type. |
| *Colwellbacteraceae* | fam. nov. | *Candidatus* Colwellbacteria (phylum) | Anantharaman *et al.* 2016 | No type material and no designated nomenclature type. |
| *Tagabacteraceae* | fam. nov. | *Candidatus* Tagabacteraceae (phylum) | Anantharaman *et al.* 2016 | No type material and no designated nomenclature type. |
| *Zambryskibacteraceae* | fam. nov. | *Candidatus* Zambryskibacteria (phylum) | Anantharaman *et al.* 2016 | No type material and no designated nomenclature type. |
| *Dehalobacteriaceae* | fam. nov. | N/A | This paper. | Proposed type is the effectively published genus ‘Dehalobacterium’; can be potentially proposed under ICNP |
| *Acidibacillaceae* | fam. nov. | N/A | This paper. | Proposed type is the effectively published genus ‘Acidibacillus’; does not satisfy SeqCode quality criteria |
| *Vermiphilaceae* | fam. nov. | N/A | This paper. | Proposed type is the effectively published genus ‘Vermiphilus’; the original authors of the type species are expected to independently propose the higher rank name* |
| *Massilibacteriaceae* | fam. nov. | N/A | This paper. | Proposed type is the effectively published genus ‘Massilibacterium’; Untrusted source |
| *Massilibacillaceae* | fam. nov. | N/A | This paper. | Proposed type is the effectively published genus ‘Massilibacillus’; Untrusted source |
| *Chloracidobacteriaceae* | fam. nov. | N/A | This paper. | Proposed type is the effectively published genus ‘Chloracidobacterium’; the original authors of the type species are expected to independently propose the higher rank name* |
| *Chloracidobacteriales* | ord. nov. | N/A | This paper. | Proposed type is the effectively published genus ‘Chloracidobacterium’; the original authors of the type species are expected to independently propose the higher rank name* |

*Personal communication
