# Supplementary material for: Proposal of names for 329 higher rank taxa defined in the Genome Taxonomy Database under two prokaryotic codes
Source: FEMS Microbiol Lett. 2023 Jul 21;370:fnad071. doi: 10.1093/femsle/fnad071 (PMC10408702; doi:10.1093/femsle/fnad071)
Supplement: fnad071_Supplemental_Files [file fnad071_supplemental_files.zip › Suppl_figures_GTDB_Latin_names_rev1.docx]

**Supplementary figures**

**
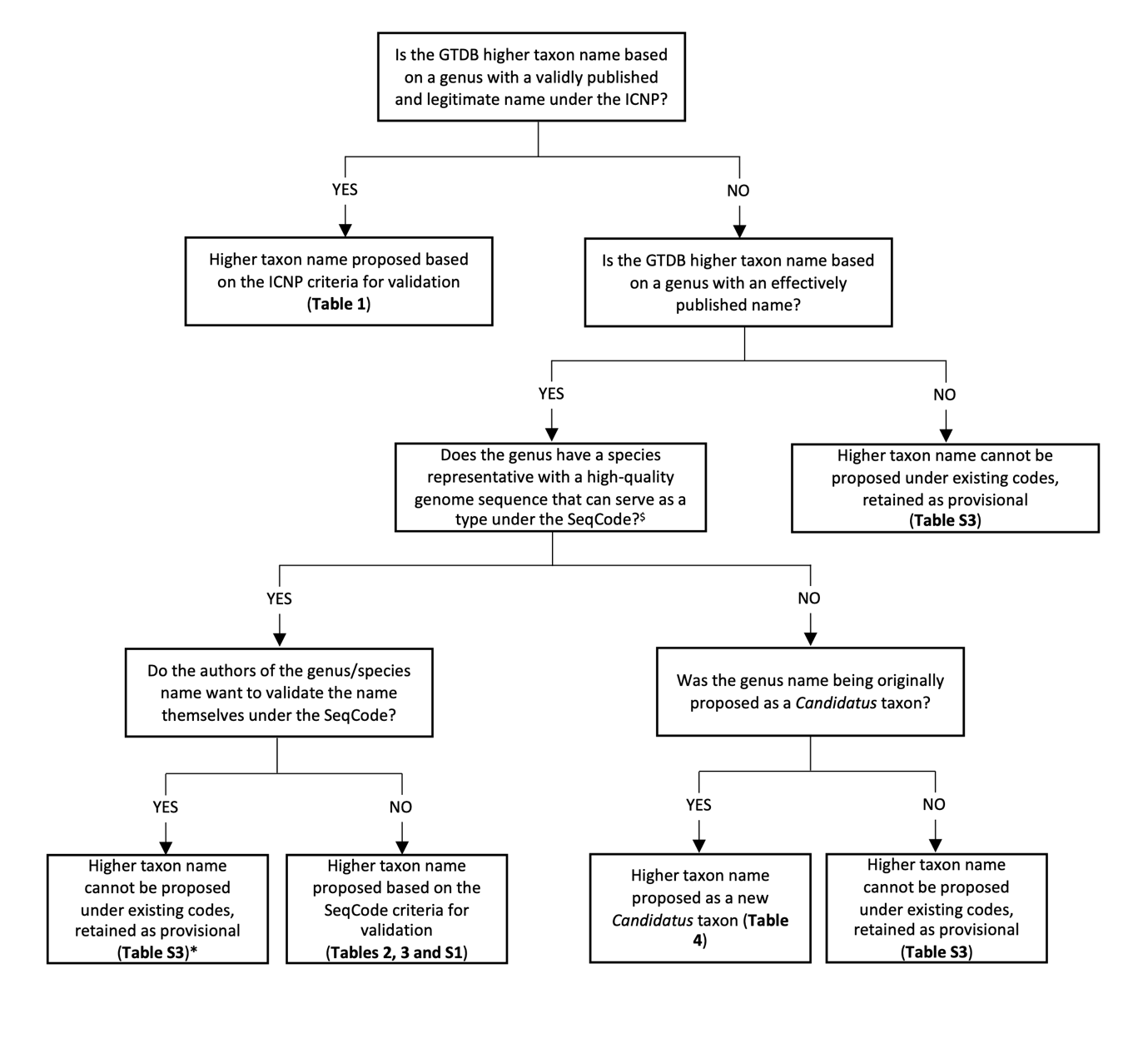
**

**Supplementary Figure 1.** Decision tree illustrating the reasoning behind the proposal of GTDB-defined higher taxon name as a new name under the ICNP, SeqCode, as a *Candidatus* or to be retained as a provisional name. ^$^ Note that some existing effectively published names of phyla have been corrected in Table S2; *the original authors of the type species may independently propose the higher taxon name.


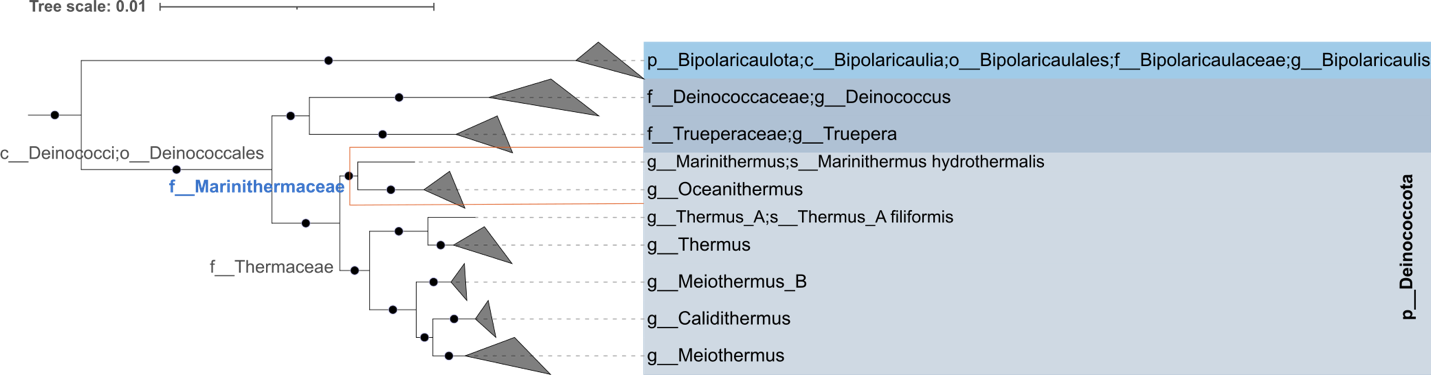


**Supplemental Figure 2.** Maximum likelihood phylogenetic inference of 120 concatenated protein markers using IQ-TREE under the LG matrix. Shading reflects phylum-level grouping, with black circles indicating >90% support. Names of taxa defined in GTDB are coloured in blue.


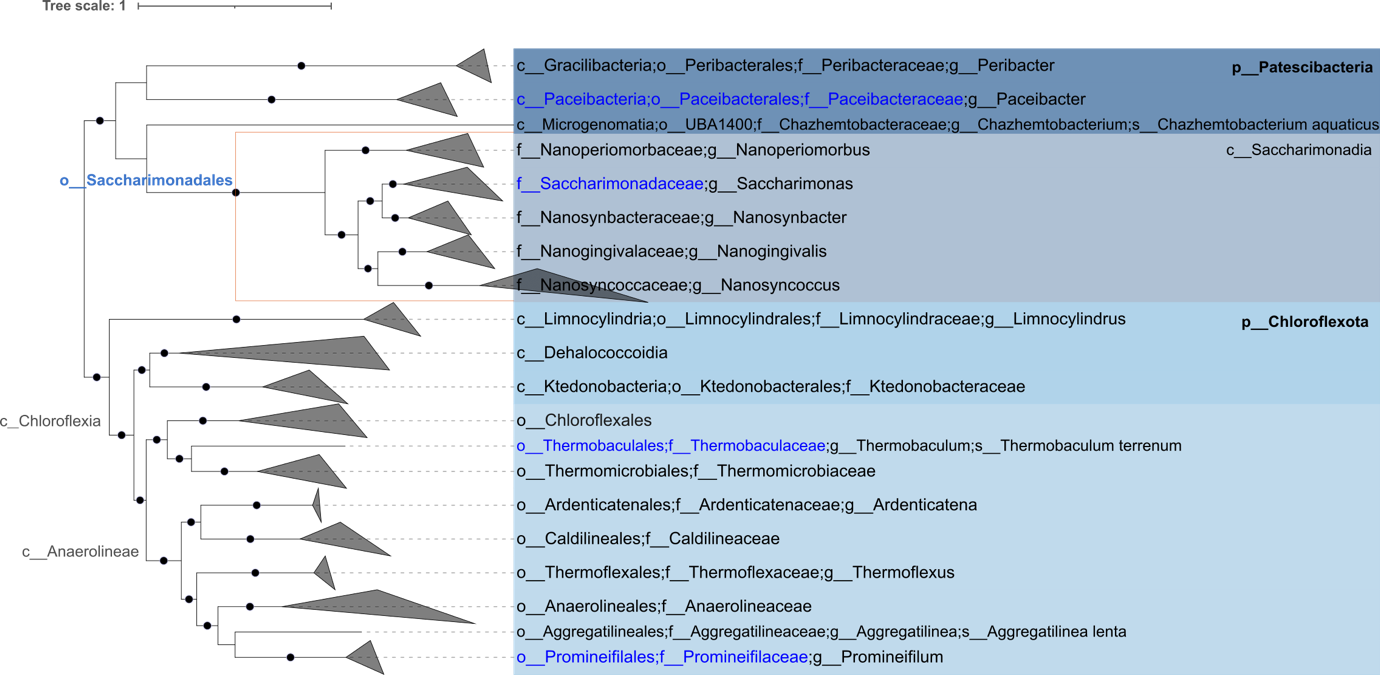


**Supplemental Figure 3.** Maximum likelihood phylogenetic inference of 120 concatenated protein markers using IQ-TREE under the LG matrix. Shading reflects phylum-level grouping, with black circles indicating >90% support. Names of taxa defined in GTDB are coloured in blue.


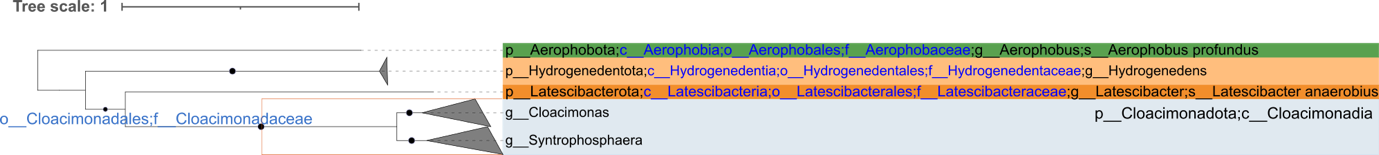


**Supplemental Figure 4.** Maximum likelihood phylogenetic inference of 120 concatenated protein markers using IQ-TREE under the LG matrix. Shading reflects phylum-level grouping, with black circles indicating >90% support. Names of taxa defined in GTDB are coloured in blue.


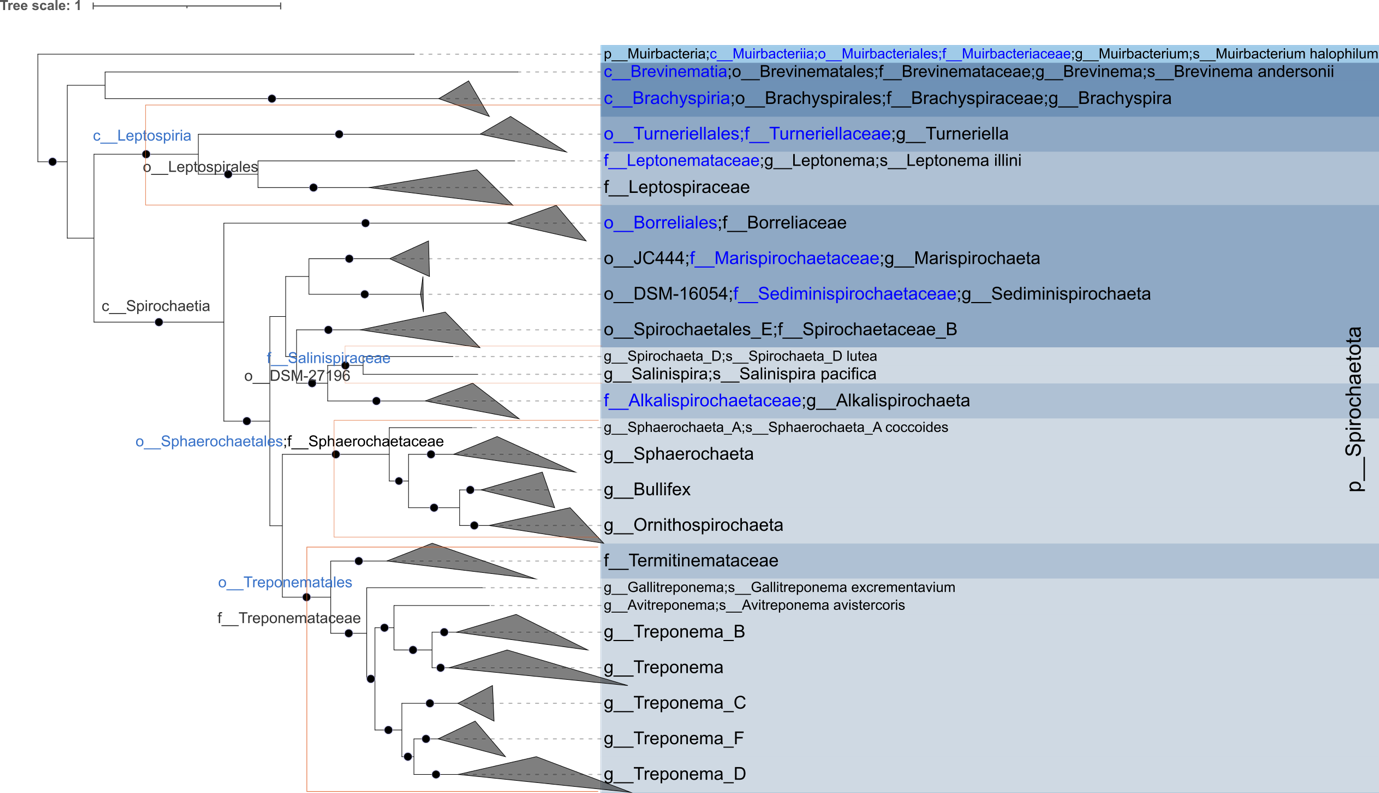


**Supplemental Figure 5.** Maximum likelihood phylogenetic inference of 120 concatenated protein markers using IQ-TREE under the LG matrix. Shading reflects phylum-level grouping, with black circles indicating >90% support. Names of taxa defined in GTDB are coloured in blue.


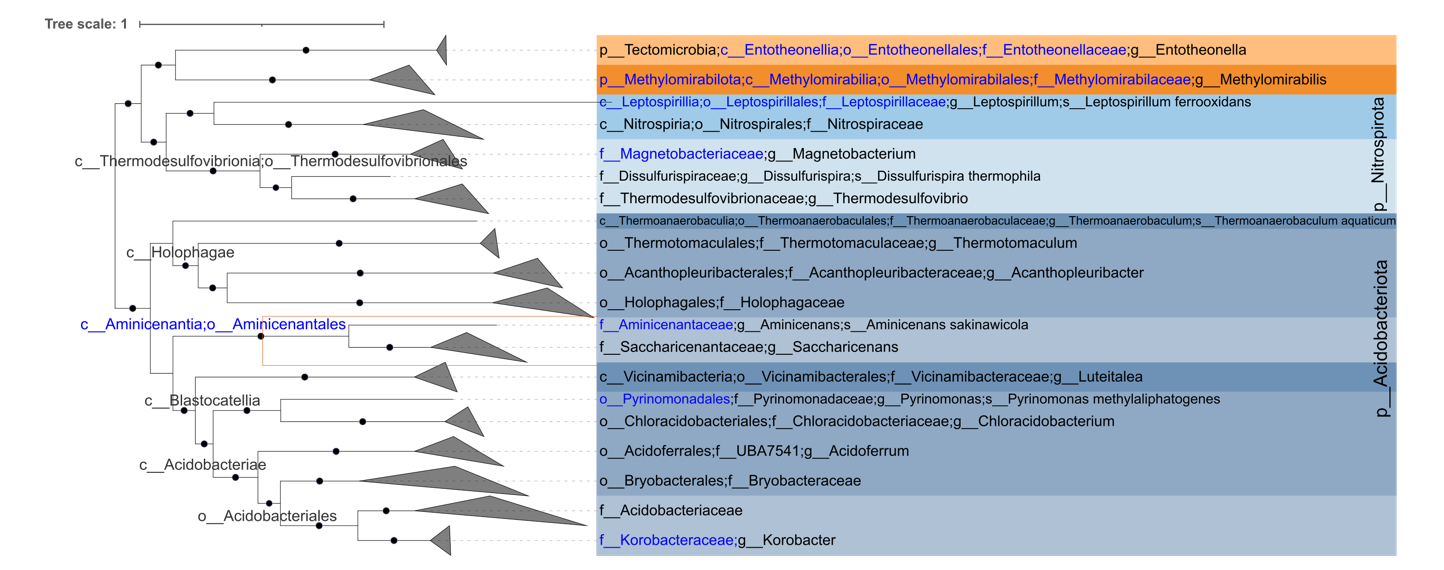


**Supplemental Figure 6.** Maximum likelihood phylogenetic inference of 120 concatenated protein markers using IQ-TREE under the LG matrix. Shading reflects phylum-level grouping, with black circles indicating >90% support. Names of taxa defined in GTDB are coloured in blue.


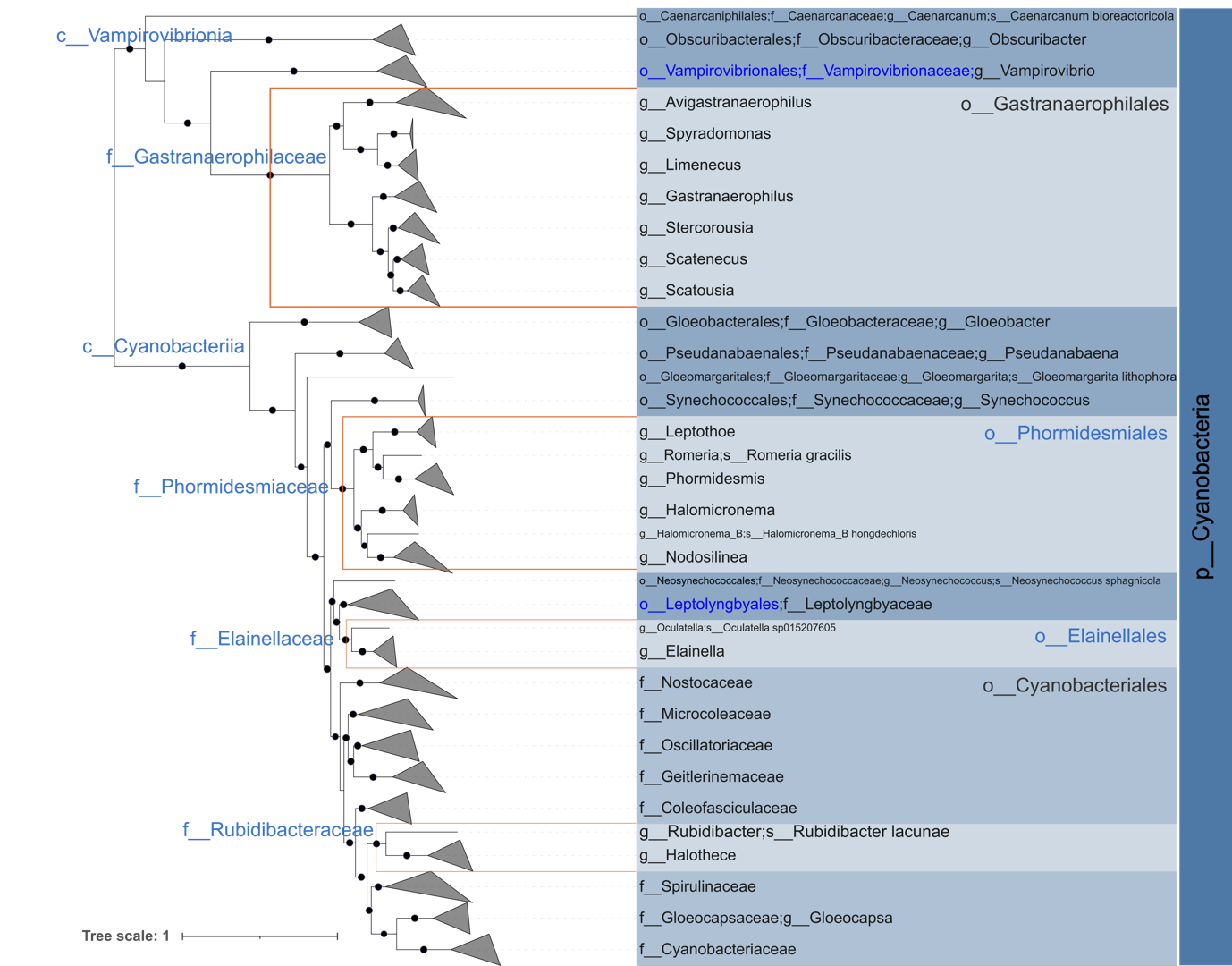


**Supplemental Figure 7.** Maximum likelihood phylogenetic inference of 120 concatenated protein markers using IQ-TREE under the LG matrix. Shading reflects phylum-level grouping, with black circles indicating >90% support. Names of taxa defined in GTDB are coloured in blue.


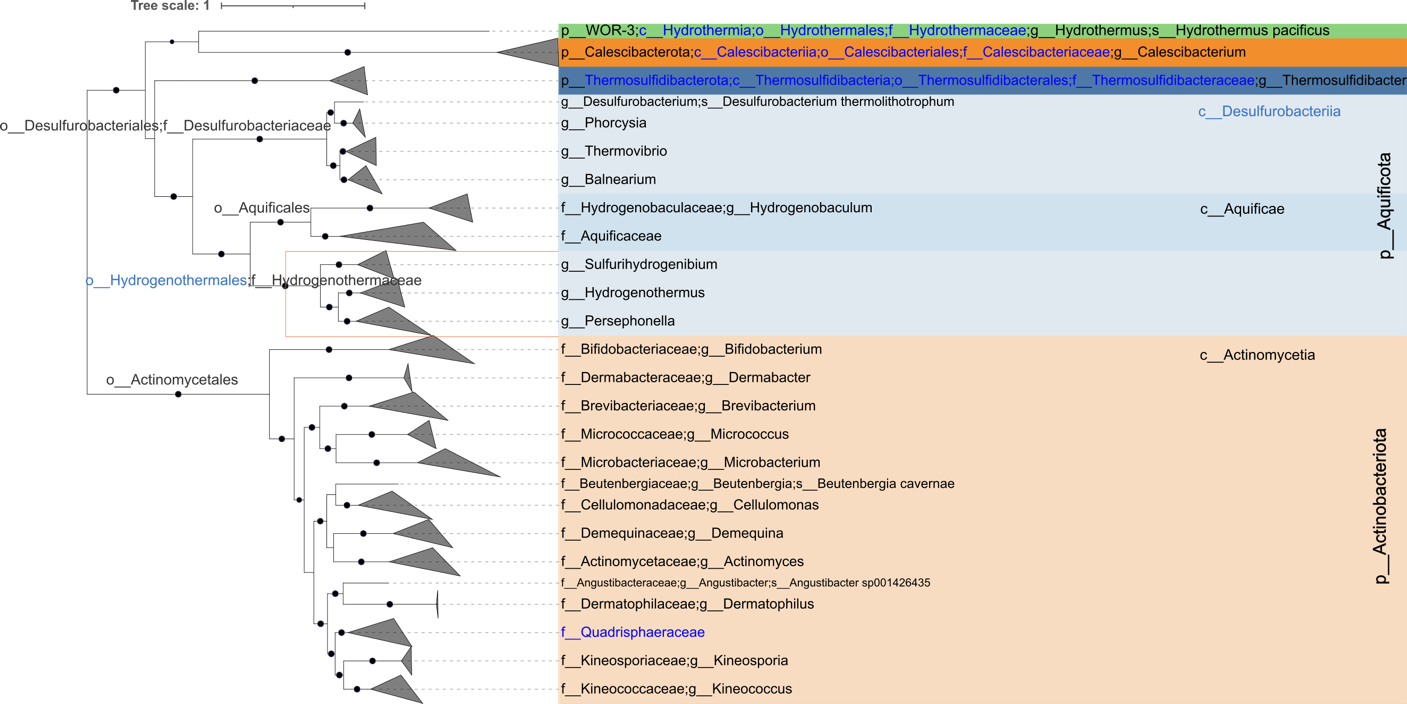


**Supplemental Figure 8.** Maximum likelihood phylogenetic inference of 120 concatenated protein markers using IQ-TREE under the LG matrix. Shading reflects phylum-level grouping, with black circles indicating >90% support. Names of taxa defined in GTDB are coloured in blue.


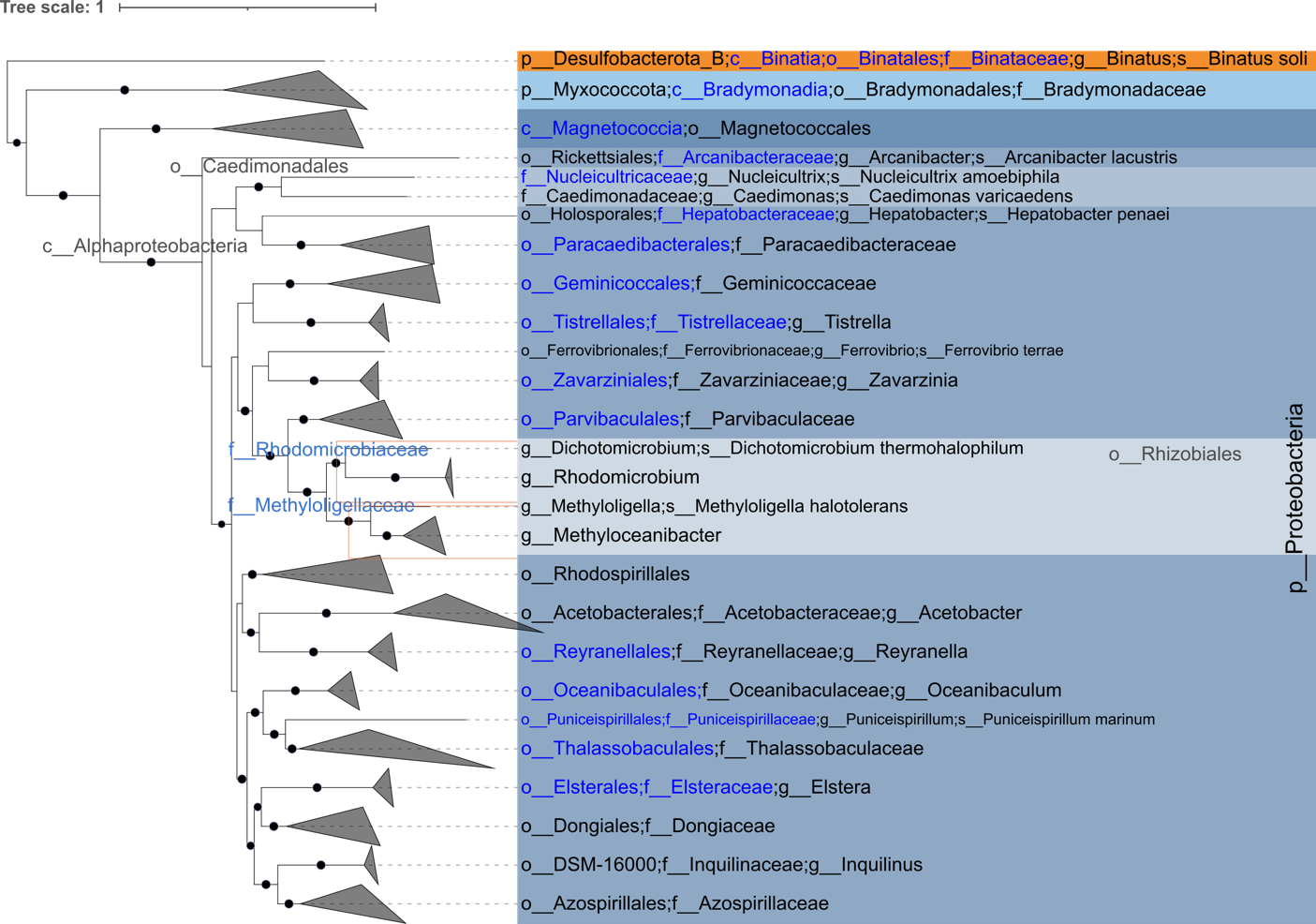


**Supplemental Figure 9.** Maximum likelihood phylogenetic inference of 120 concatenated protein markers using IQ-TREE under the LG matrix. Shading reflects phylum-level grouping, with black circles indicating >90% support. Names of taxa defined in GTDB are coloured in blue.


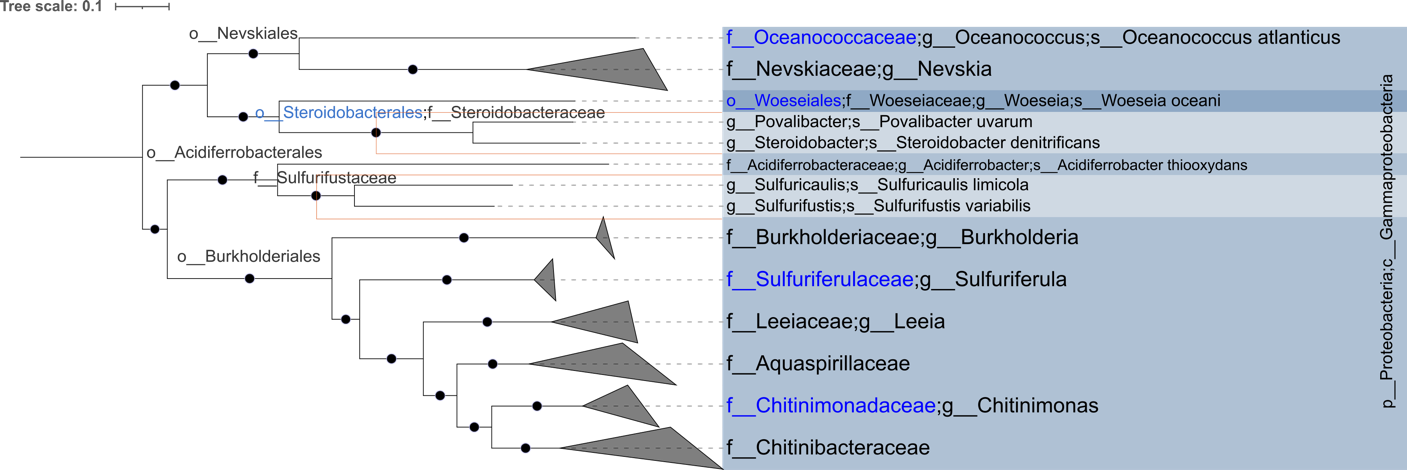


**Supplemental Figure 10.** Maximum likelihood phylogenetic inference of 120 concatenated protein markers using IQ-TREE under the LG matrix. Shading reflects phylum-level grouping, with black circles indicating >90% support. Names of taxa defined in GTDB are coloured in blue.


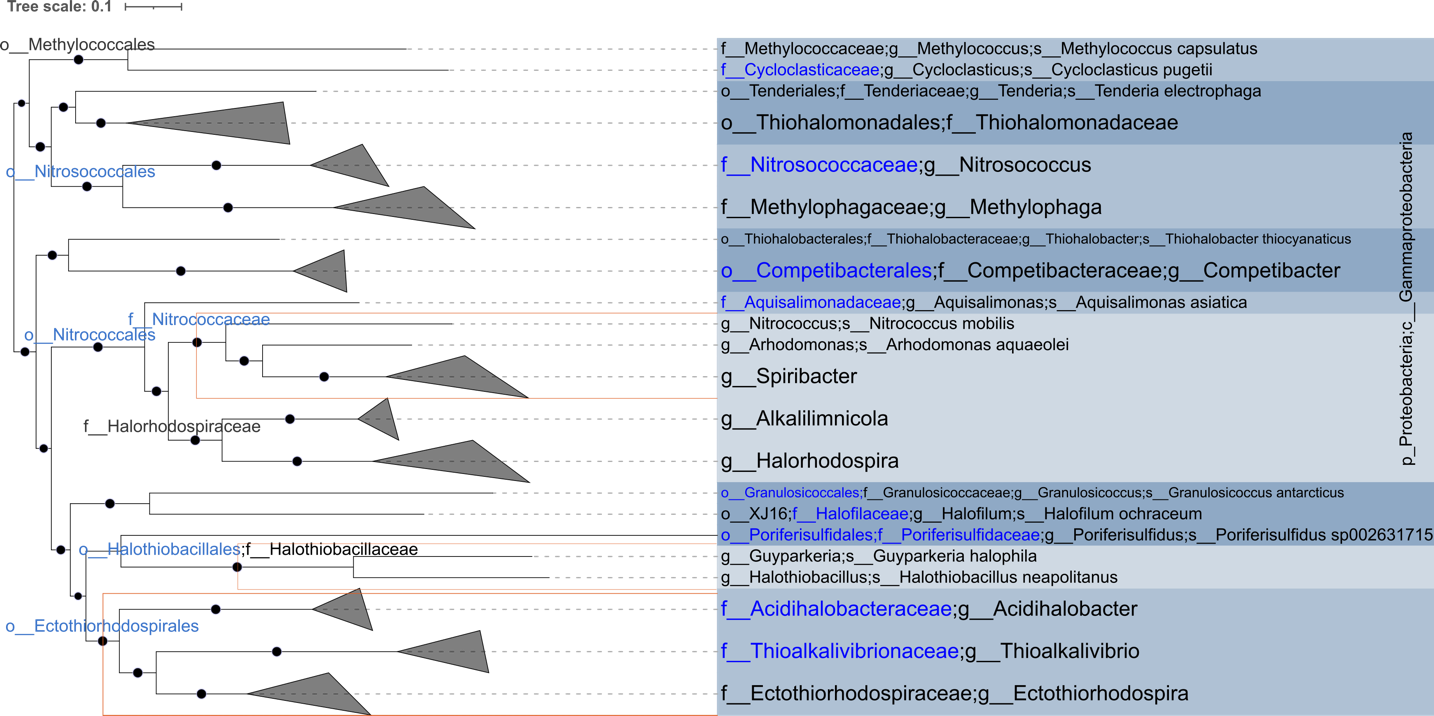


**Supplemental Figure 11.** Maximum likelihood phylogenetic inference of 120 concatenated protein markers using IQ-TREE under the LG matrix. Shading reflects phylum-level grouping, with black circles indicating >90% support. Names of taxa defined in GTDB are coloured in blue.

**
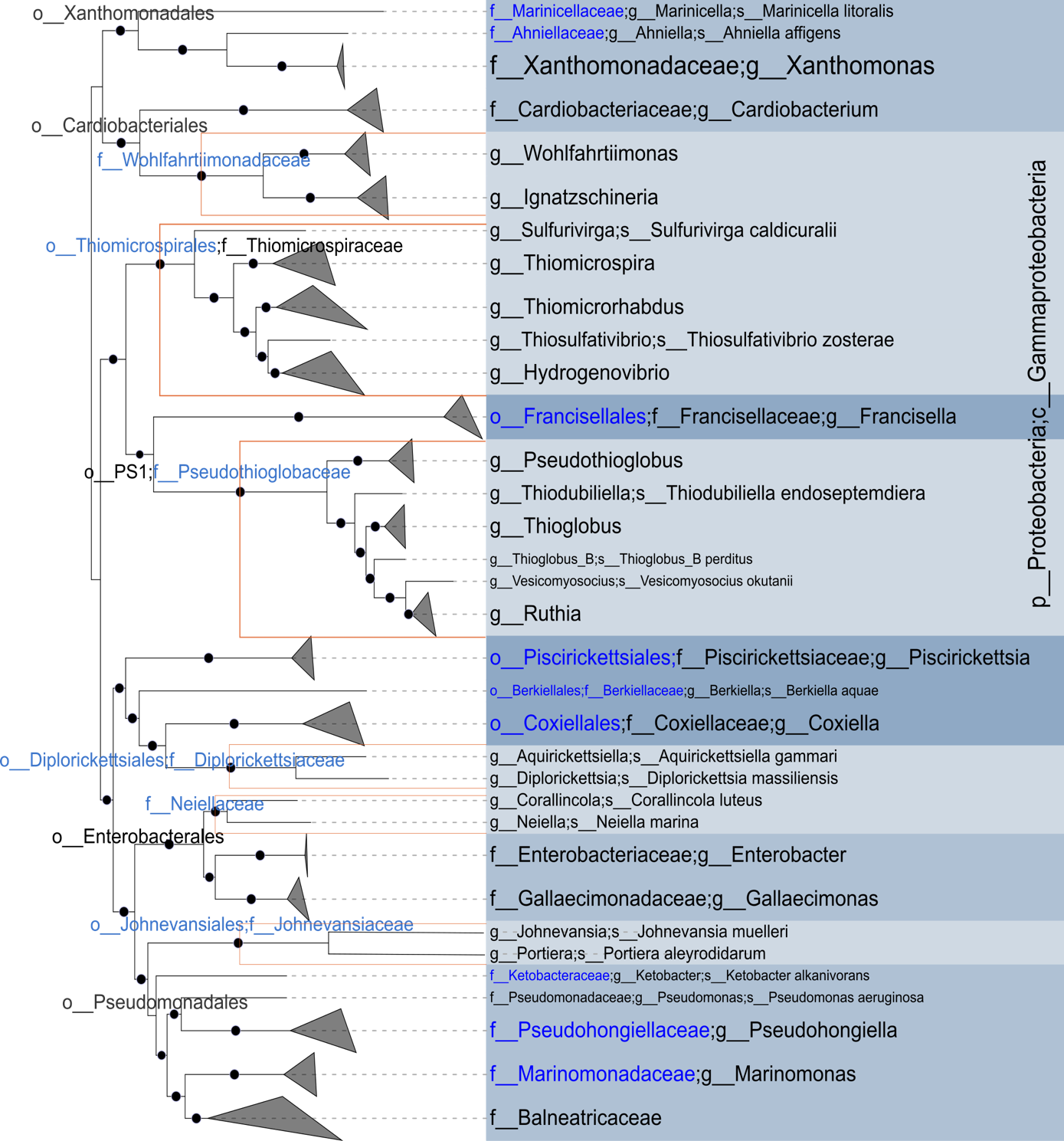
**

**Supplemental Figure 12.** Maximum likelihood phylogenetic inference of 120 concatenated protein markers using IQ-TREE under the LG matrix. Shading reflects phylum-level grouping, with black circles indicating >90% support. Names of taxa defined in GTDB are coloured in blue.

**
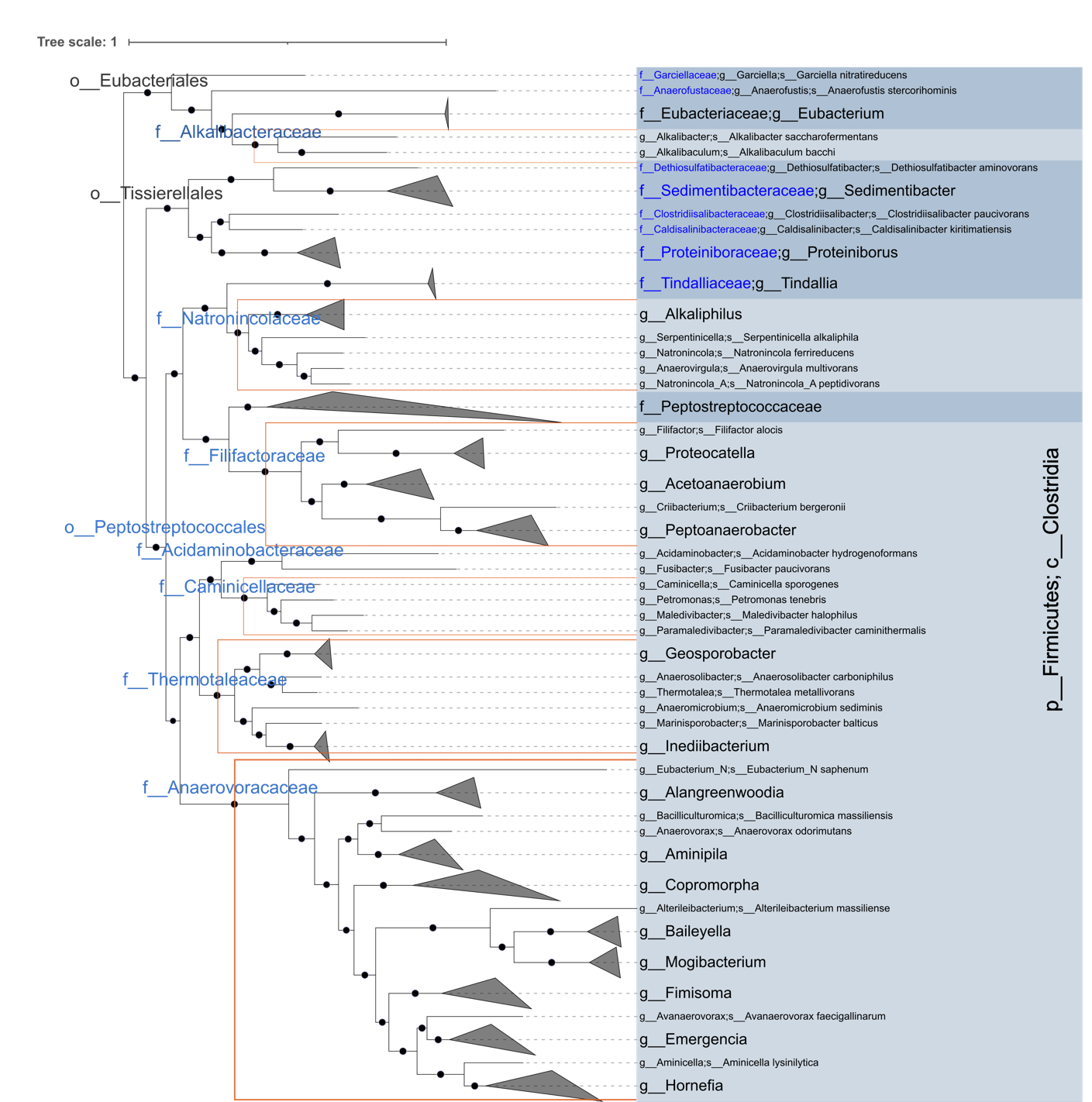
**

**Supplemental Figure 13.** Maximum likelihood phylogenetic inference of 120 concatenated protein markers using IQ-TREE under the LG matrix. Shading reflects phylum-level grouping, with black circles indicating >90% support. Names of taxa defined in GTDB are coloured in blue.

**
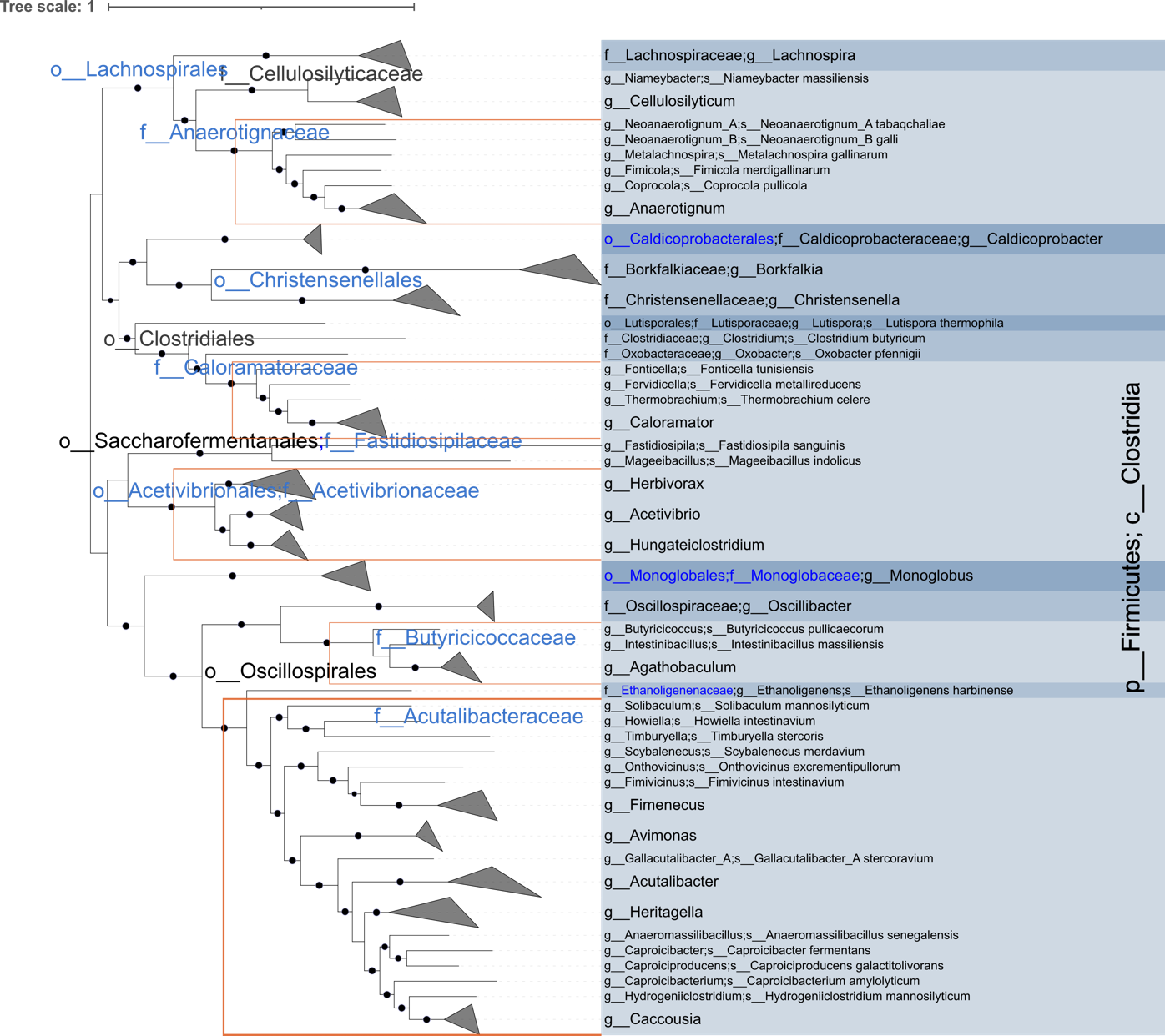
**

**Supplemental Figure 14.** Maximum likelihood phylogenetic inference of 120 concatenated protein markers using IQ-TREE under the LG matrix. Shading reflects phylum-level grouping, with black circles indicating >90% support. Names of taxa defined in GTDB are coloured in blue.

**
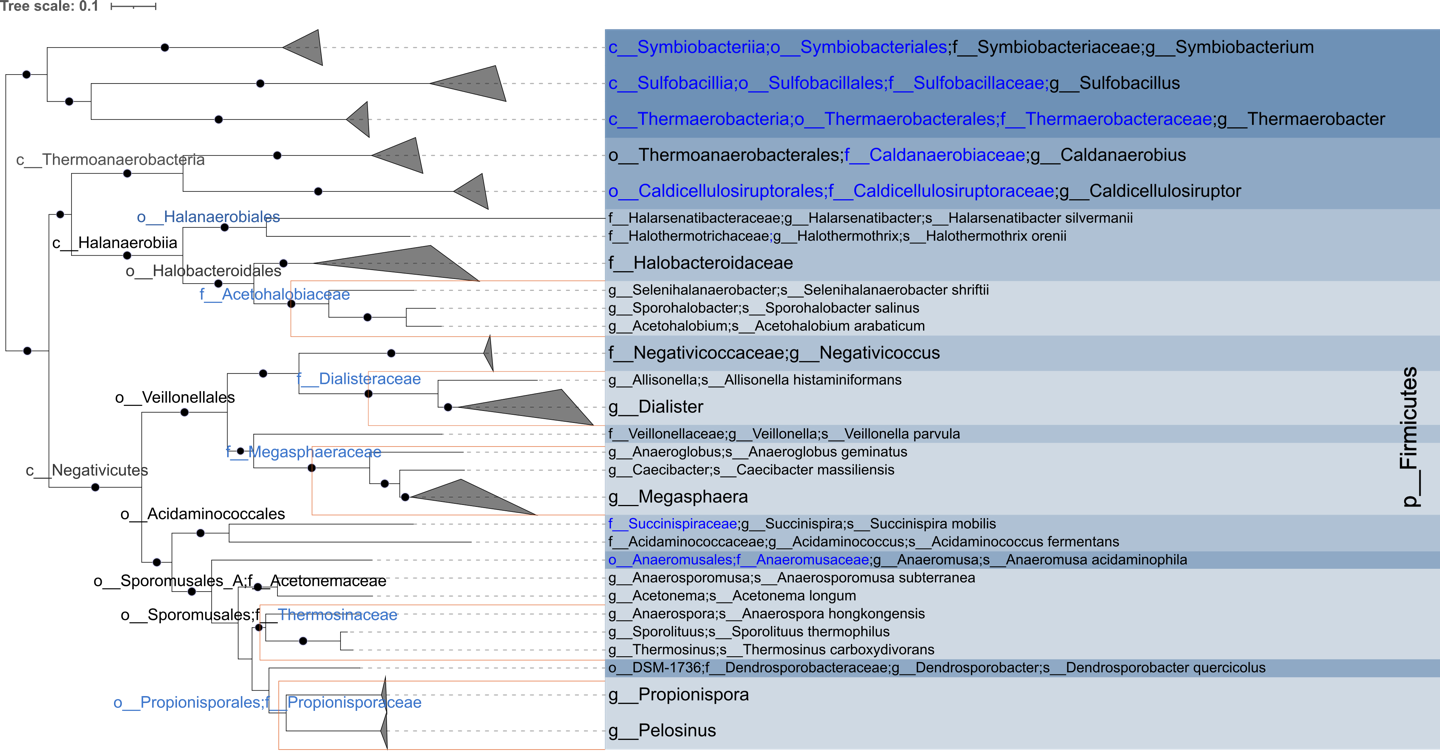
**

**Supplemental Figure 15.** Maximum likelihood phylogenetic inference of 120 concatenated protein markers using IQ-TREE under the LG matrix. Shading reflects phylum-level grouping, with black circles indicating >90% support. Names of taxa defined in GTDB are coloured in blue.

**
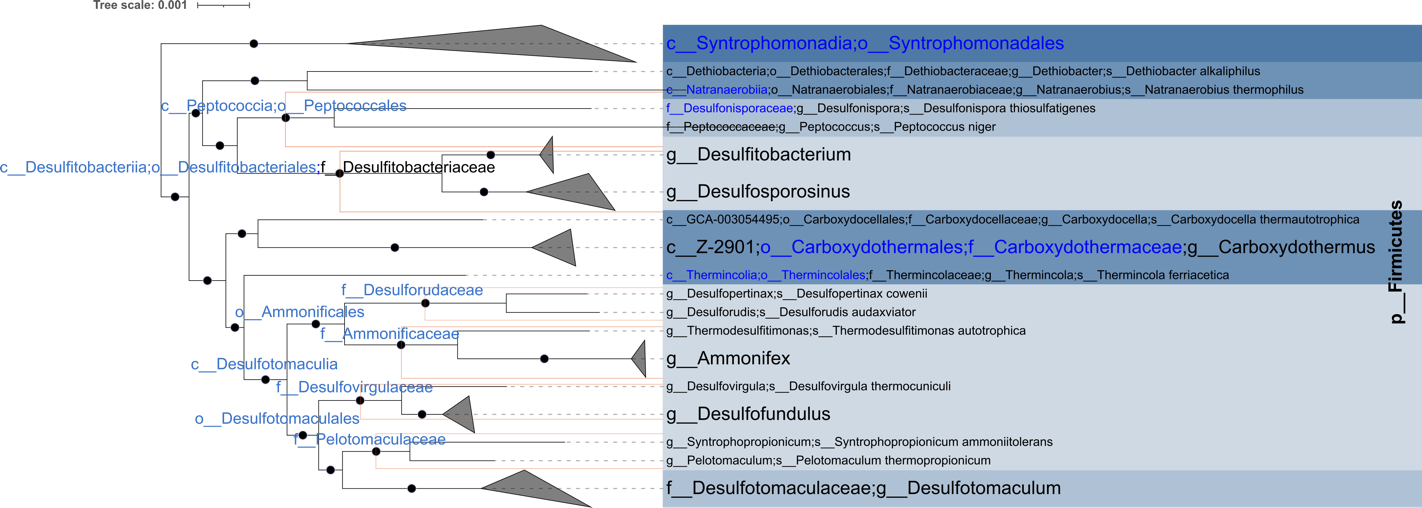
**

**Supplemental Figure 16.** Maximum likelihood phylogenetic inference of 120 concatenated protein markers using IQ-TREE under the LG matrix. Shading reflects phylum-level grouping, with black circles indicating >90% support. Names of taxa defined in GTDB are coloured in blue.

**
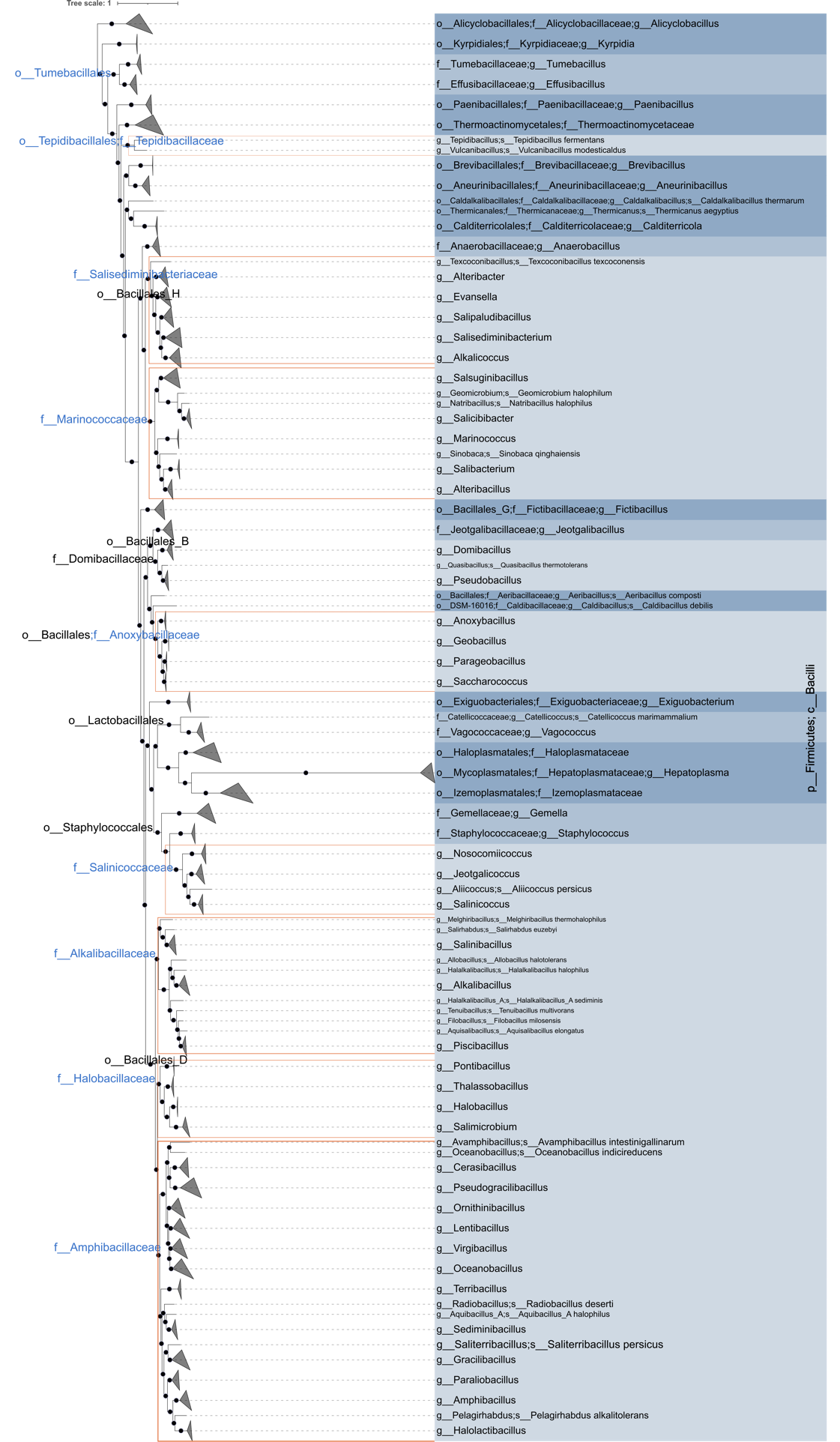
**

**Supplemental Figure 17.** Maximum likelihood phylogenetic inference of 120 concatenated protein markers using IQ-TREE under the LG matrix. Shading reflects phylum-level grouping, with black circles indicating >90% support. Names of taxa defined in GTDB are coloured in blue.

**
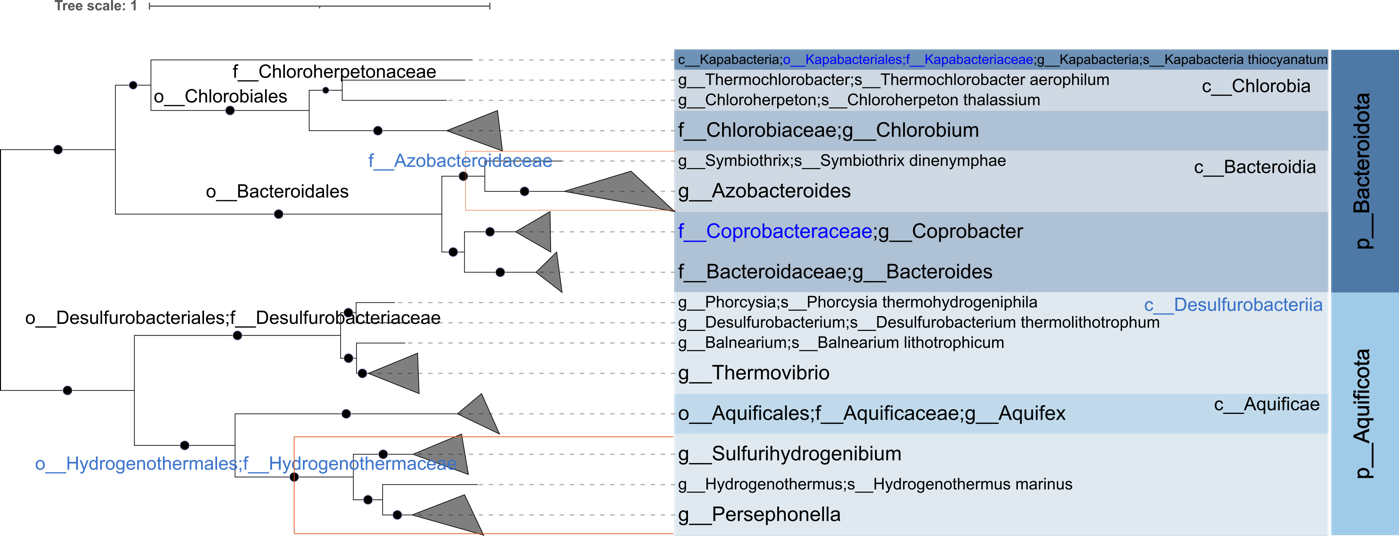
**

**Supplemental Figure 18.** Maximum likelihood phylogenetic inference of 120 concatenated protein markers using IQ-TREE under the LG matrix. Shading reflects phylum-level grouping, with black circles indicating >90% support. Names of taxa defined in GTDB are coloured in blue.


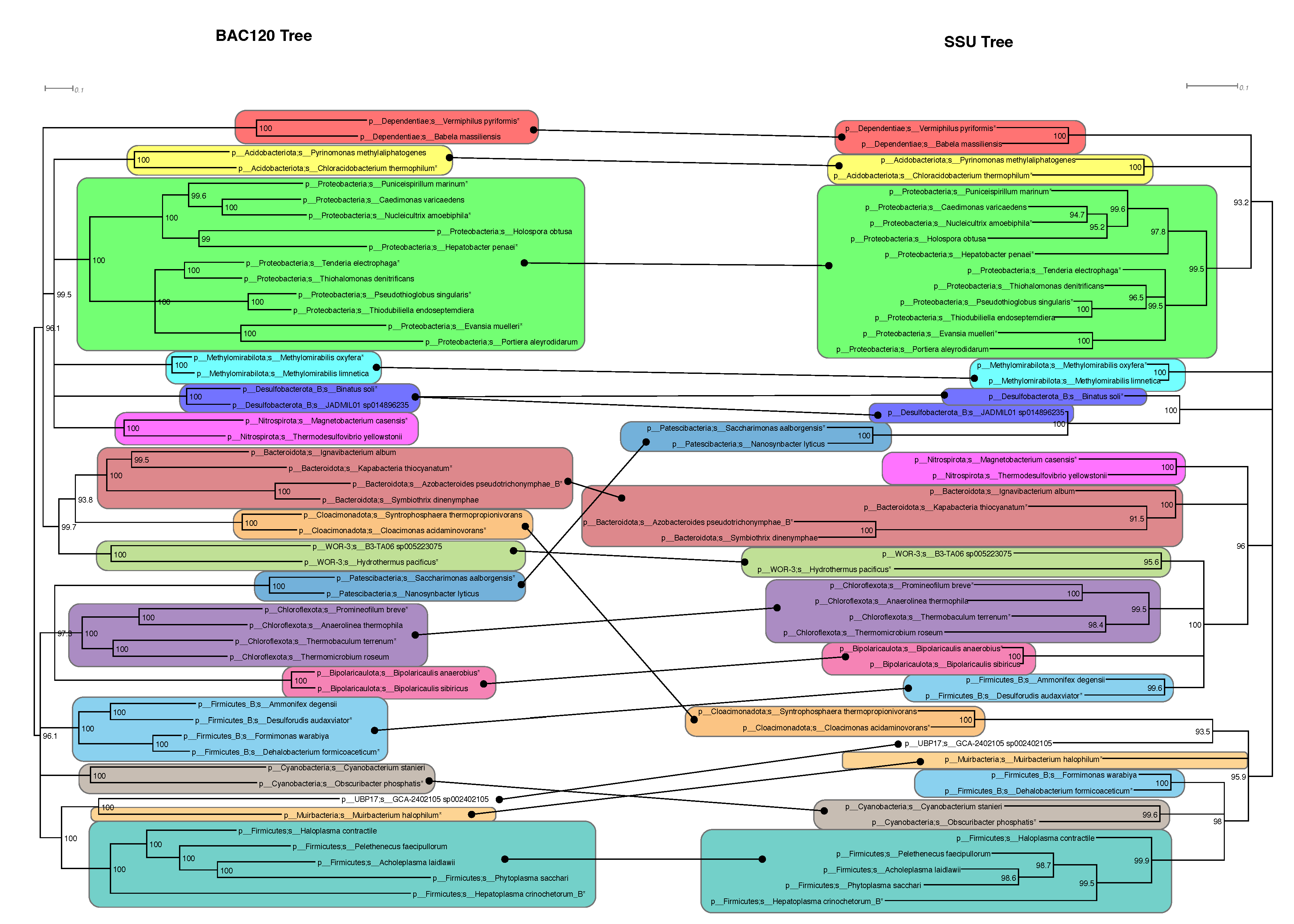


**Supplemental Figure 19.** Species-level phylogenies illustrating congruences between 16S rRNA gene and bac120 marker set taxonomies. Coloured boxes reflect phylum-level grouping and names applied to type species proposed under the SeqCode indicated with asterisk.
